# The active roles of *Rhodotorula mucilaginosa* ZTHY2 in regulating antioxidant capacity and immune function of Leizhou black ducks

**DOI:** 10.3389/fvets.2025.1494892

**Published:** 2025-01-30

**Authors:** Jiang Wu, Yingxin Hu, Namula Zhao, Wei Yang, Zhibao Chen

**Affiliations:** ^1^Department of Veterinary Medicine, College of Coastal Agricultural Sciences, Guangdong Ocean University, Zhanjiang, China; ^2^College of Veterinary Medicine, South China Agricultural University, Guangzhou, China; ^3^South China Branch of National Saline-Alkali Tolerant Rice Technology Innovation Center Zhanjiang, Zhanjiang, China

**Keywords:** *Rhodotorula mucilaginosa* ZTHY2, Leizhou black duck, antioxidant capacity, immune function, growth performance

## Abstract

Previous studies in mice have demonstrated that *Rhodotorula mucilaginosa* ZTHY2 can promote animal growth, enhance antioxidant and immune functions, and regulate intestinal flora in our laboratory. This study focuses on the Leizhou black duck, a local breed in Zhanjiang, to evaluate the effects of *Rhodotorula mucilaginosa* ZTHY2 on its growth, antioxidant capacity, and immune function. A total of 150 1-day-old male Leizhou black ducks, of similar size and healthy, were selected for this study and randomly assigned to five treatment groups. Each group contained three replicates with ten birds each. The control group (Control) was given a standard basal diet, while the RM group received a diet supplemented with ZTHY2 at concentrations of 2 × 10^7^ (RM1), 2 × 10^8^(RM2), or 2 × 10^9^(RM3) CFU/kg, respectively. The LA group was supplemented with 2 × 10^9^ CFU/kg of *Lactobacillus acidophilus* in addition to the basal diet. The feeding trial lasted 42 days. The analysis revealed significant improvements in the average body weight for the RM2 and RM3 groups, which were significantly higher than that of the control group (*p <* 0.05 and *p <* 0.01). Treatment with ZTHY2 induced a dose-dependent elevation in superoxide dismutase (SOD), catalase (CAT), glutathione peroxidase (GSH-Px), and SOD activities, and a reduction in malondialdehyde (MDA) content in the serum at 42 days. The serum levels of complement components C3 and C4, immunoglobulin IgG, and cytokines IFN-*γ*, IL-2, IL-4, IL-6, and TNF-*α* were significantly increased in Leizhou black ducks treated with ZTHY2 at 42-days post-treatment, with the therapeutic effect becoming more pronounced as the duration of the experiment prolonged. The greatest impact was observed at a dosage of 2 × 10^9^ CFU/kg of ZTHY2. Moreover, ZTHY2 modulated the mRNA expression profiles of these cytokines in the thymus, spleen, and bursa, thereby sustaining the balance of immune dynamics. In summary, the supplementation of *Rhodotorula mucilaginosa* ZTHY2 at a dosage of 2 × 10^9^ CFU/kg had been found to most effectively enhance the growth performance of Leizhou black ducks by optimizing their immune function and antioxidant capacity.

## 1 Introduction

Oxidative stress is a subject of much attention in the field of immunology because it is closely linked to the decline of immune organ and immune cell function (1, 2). Under physiological conditions, a moderate increase in the level of oxidative stress can act as a signal to activate immune cells, promote their proliferation and differentiation, and thus enhance the body’s immune response to pathogens. The activation effect of moderate oxidative stress on the immune system reflects the “double-edged sword” characteristic of redox balance in organisms, that is, it is beneficial to the body within a certain range, but may turn into harmful factors beyond a certain limit (3). However, when the level of oxidative stress exceeds the body’s physiological regulatory capacity, that is, when it exceeds a certain threshold, it will begin to have a negative impact on the immune system and weaken immune defenses (4). Under physiological conditions, a moderate increase in the level of oxidative stress can act as a signal to activate immune cells, promote their proliferation and differentiation, and thus enhance the body’s immune response to pathogens. The activation effect of moderate oxidative stress on the immune system reflects the “double-edged sword” characteristic of redox balance in organisms, that is, it is beneficial to the body within a certain range, but may turn into harmful factors beyond a certain limit. However, when the level of oxidative stress exceeds the body’s physiological regulatory capacity, that is, when it exceeds a certain threshold, it will begin to have a negative impact on the immune system and weaken immune defenses (3, 5). Therefore, maintaining a delicate balance between oxidative stress and antioxidant mechanisms is essential to maintaining the integrity and effectiveness of the immune system. This requires the body’s antioxidant system, including enzymatic (such as superoxide dismutase, glutathione peroxidase, etc.) and non-enzymatic (such as vitamin C, glutathione, etc.) antioxidants, to effectively remove excess ROS and protect immune cells from oxidative damage (6, 7). Proper supplementation of antioxidants can help maintain this balance, thereby maintaining and enhancing the body’s immune function.

*Lactobacillus acidophilus* and other probiotics such as *Bacillus subtilis* and Marine red yeast show great potential in the field of animal nutrition and health. They can not only enhance the immune response of animals, but also improve intestinal health and provide a solid foundation for the growth and development of animals (8, 9). With further research into the function of these probiotics, we are expected to discover more new applications that will benefit animal health and production. Currently, *Lactobacillus acidophilus* was identified as a probiotic in the Catalog of Feed Additives (2013) published by the Ministry of Agriculture and Rural Affairs of China. Extensive experimental data had shown that the incorporation of a *Lactobacillus acidophilus* bacterial preparation into the diet significantly enhances the integrity of the intestinal epithelial barrier, maintains the balance of the immune system, and increases the diversity of the intestinal microbiota in laying hens (9). A recent investigation demonstrated that the incorporation of 0.5g/kg of *Bacillus subtilis* into the dietary regimen of laying hens effectively suppressed intestinal inflammation, enhanced antioxidant status, and maintained duodenal barrier integrity (10). Marine red yeast is a kind of microorganism that exists naturally in the marine environment and has been widely used in animal production in recent years due to its rich nutritional value and immune-enhancing effect. Marine red yeast is rich in protein, amino acid, unsaturated fatty acid, astaxanthin, beta-carotene, polysaccharide and other nutrients (11, 12). These ingredients not only provide animals with essential nutrients, but also have the potential to enhance the body’s immune function. Adding marine red yeast to laying hens’ feed can improve laying performance by regulating small intestine homeostasis (8). This suggests that Marine *rhodoyeast* may also play an important role in poultry production.

*Rhodotorula mucilaginosa* belongs to the genus *Rhodotorula*. It has strong stress resistance, can survive in a variety of environments, and has chemoheterotrophic, thermophilic, acidophilic, facultative anaerobic characteristics (11, 13, 14). The metabolites, such as astaxanthin and polysaccharides, produced during the growth of *Rhodotorula mucilaginosa*, have the ability to regulate the expression of antioxidant enzymes and remove oxygen free radicals, showing remarkable antioxidant properties (13–15). Our early research has confirmed that *Rhodotorula mucilaginosa* ZTHY2 is safe, residual-free, and capable of producing a variety of nutritionally beneficial metabolites. These metabolites are able to promote mice growth, enhance antioxidant and immune system function, and reshape the gut microbiota, showing their potential applications in the field of animal nutrition and health; and gavage with *R. mucilaginosa* ZTHY2 at the concentration of 2 × 10^8^ CFU/mL or 2 × 10^9^ CFU/mL could effectively increase the content of GSH, SOD, and CAT in immunosuppressed mice, remove excess ROS and reduce MDA (11). When co-cultured with other yeasts, the antioxidant properties of *Dendrobium kumungensis* could be significantly enhanced, and the immunomodulatory effects of macrophages could be enhanced by increasing the contents of phenols, flavonoids and B vitamins (16). Given the above properties of *Rhodotorula mucilaginosa*, it has the potential to replace antibiotics for feeding purposes. As a non-toxic, residual-free and drug-resistant microbe, it can be used as a natural feed additive to improve digestibility and gut health of animals while regulating the gut microbiota. With the further research and development of the characteristics of this microorganism, its application in the future will be more extensive.

In the field of probiotic research, *Rhodotorula mucilaginosa*, as one of them, has shown remarkable effects in regulating the body’s antioxidant and immune function. Studies have shown that the bacteria can protect the body from oxidative stress by enhancing the activity of antioxidant enzymes and reducing the production of free radicals. Polysaccharide EPS-1, which was extracted from the fermentation broth of *Bifidobacterium breva*, is a kind of polysaccharide rich in mannose, and its unique structural characteristics give it immunomodulatory activity. Studies have shown that EPS-1 can activate macrophages and enhance their ability to engulf and kill pathogens, thus playing a key role in the immune response (17). In aquaculture, *Rhodosaccharomyces vulgaris* as a feed additive has been shown to significantly enhance the immune response of tilapia. By adding 0.5 to 1% of *Rhodiomyces vulgaris* in the feed, it can improve the resistance of fish to disease and reduce the occurrence of disease, thereby improving the efficiency and economic benefits of breeding (18). Feeding experiments with *saccharomyces* extract showed that serum levels of key immune indicators such as immunoglobulin, IL-2 and IFN-*γ* were significantly increased in experimental animals. These results show that *saccharomyces* extract can effectively enhance the humoral and cellular immune response of experimental animals and improve their defense against pathogens (19). The study by Liu et al. further confirms the potential of marine *rhodosaccharomyces rubra* for use in animal nutrition and health. By adding 0.1–0.5% marine red yeast to lamb milk replacer, the intestinal immunity of lambs can be significantly enhanced, the incidence of diarrhea in early weaned lambs can be reduced, and the antioxidant capacity of serum can be increased (12). These results provide a strong scientific basis for the use of Marine *rhodotorula* as a natural and safe feed additive. In conclusion, the study of probiotics not only reveals their important role in enhancing the host immune response, but also provides new ideas for the development of novel feed additives and alternative antibiotics.

The magnitude of China’s meat duck industry was pivotal within the international market landscape (20). The Leizhou Black Duck possesses attributes such as succulent meat, high egg-laying capacity, and resilience to coarse feed, among others, and it represented a breed of duck with distinct local characteristics that was suitable for dual-purpose egg and meat production (21, 22). In the current context of increasing concern over antibiotic resistance (23), the development of natural substances with antioxidant properties as alternatives to antibiotics had become a global research hotspot. These natural antioxidants not only inhibit oxidation reactions and reduce the generation of free radicals but also enhance animal immune systems through various mechanisms, thereby promoting animal health and growth without the use of antibiotics. *Rhodotorula mucilaginosa* ZTHY2 possesses probiotic attributes, as a natural probiotic, it exhibited considerable potential in replacing conventional antibiotic additives within animal feed formulations (11). By maintaining the dynamic balance of immune function, ZTHY2 can improve animal immunity and enhance antioxidant function, which suggests that ZTHY2 has a good application potential in livestock production.

In this study, Leizhou black duck with local characteristics in Zhanjiang city was selected as the research object, the effects of *Rhodotorula mucilaginosa* ZTHY2 were explored on the growth performance, antioxidant capacity and immune function of the ducks. The results showed that ZTHY2 promoted the regulation of immune function and antioxidant capacity of Leizhou black ducks, and improved the growth performance of the ducks, with the best effect when the supplemental level was 2 × 10^9^ CFU/kg. The objective was to illuminate the potential roles of ZTHY2 as a substitute feed additive and to explore additional strategies for bolstering animal immunity and enhancing breeding efficiency.

## 2 Materials and methods

### 2.1 Experimental strains of bacteria

The bacterium *Rhodotorula mucilaginosa* ZTHY2 was isolated and purified from the marine environment of the Leizhou Peninsula in China. It was currently preserved at the Chinese Typical Culture Preservation Center, with the accession number M2015296. *Lactobacillus acidophilus* (BNCC185342) was acquired from Beina Biotechnology Co., LTD. China.

### 2.2 Experimental animals and group design

A total of 150 healthy, similarly sized, 1-day-old male Leizhou black ducks were selected and randomly assigned to five treatment groups, each with three replicates and 10 birds per replicate. The hens in the control group were provided with a standard basal diet, whereas the RM groups received the basal diet supplemented with 2 × 10^7^ CFU/kg, 2 × 10^8^ CFU/kg, or 2 × 10^9^ CFU/kg of *Rhodotorula mucilaginosa* ZTHY2, respectively, named as Control (Control group), RM1 (2 × 10^7^ CFU/kg RM group), RM2 (2 × 10^8^ CFU/kg RM group), and RM3 (2 × 10^9^ CFU/kg RM group). Add dosage and quality control, refer to our previous research report in mice (The Rl, Rm, and Rh groups were treated with 0.5 mL of *R. mucilaginosa* ZTHY2 with concentrations of 2 × 10^7^ CFU/mL, 2 × 10^8^ CFU/mL, and 2 × 10^9^ CFU/mL, respectively) (11). Additionally, the LA group was fed a diet supplemented with 2 × 10^9^ CFU/kg of *Lactobacillus acidophilus*, named LA (2 × 10^9^ CFU/kg LA group). The feeding trial lasted for a duration of 42 days. All experimental protocols were approved by the Animal Ethics Committee of Guangdong Ocean University, China (IACUC No. GDOU-LAE-2020-007). The experimental feeding method was indoor flat feeding with mixed materials. Clean the house regularly and keep ventilation to observe the health status of Leizhou black ducks.

### 2.3 Feeding management and basic feed

Prior to the trial, the duck housing and utensils underwent a complete cleaning and disinfection process. After proper ventilation and purification, the experiment began. It employed an indoor, horizontal feeding system with a varied diet. The Leizhou black ducks had continuous access to feed and water, while the facility’s temperature, humidity, and lighting were carefully controlled. Regular cleaning maintained good ventilation. The ducks’ health was continually monitored, with a focus on the drinking water quality. Timely feed and water supply were ensured, with detailed records kept. The feeding and vaccination protocols followed standard industry practices. The basal feed met the requirements of the Meat Duck Feeding Standard (NY/T 2122–2012), and its composition and nutritional level were detailed in Table 1.

**Table 1:** Dietary composition and nutrient concentrations of basal diets (air-dry basis) in per-centages.

Items	1–21 days	22–42 days
Ingredients		
Corn	61.85	64.60
Soybean meal	28.50	25.30
Wheat bran	3.25	3.14
Fish meal	2.50	2.80
NaCl	0.30	0.30
Limestone	1.30	1.29
CaHPO_4_	1.28	1.26
DL-Met	0.19	0.21
Premix*	1.00	1.10
Total	100.00	100.00
Nutrition levels		
ME/(MJ/kg)	12.24	12.35
CP	20.70	18.60
Ca	0.98	0.93
AP	0.45	0.40
Lys	1.10	0.86
Met	0.50	0.45

*The dietary premix supplies the following nutritional components per kilogram of starter: Vitamin A, 8,000 International Units (IU); Vitamin D, 2,000 IU; Vitamin E, 20 IU; Vitamin B12, 12 milligrams (mg); Vitamin K3, 2 mg; nicotinic acid, 40 mg; D-pantothenic acid, 15 mg; folic acid, 1 mg; iodine, 0.40 mg; iron, 60 mg; copper, 8 mg; zinc, 60 mg; manganese, 100 mg; and selenium, 0.35 mg.

### 2.4 Sample collection

On days 21 and 42, two Leizhou black ducks with similar body weights from each group were randomly chosen. After a 12-h fast, blood was drawn from the subclavian vein. Then, 10 mL of blood was allowed to clot at room temperature and centrifuged at 4°C, 3000 rpm for 15 min to separate the serum. This serum was stored at −20°C for later analysis. Following this, the ducks were euthanized, and their thymus, spleen, and bursa were removed and washed with sterile PBS. They were then flash-frozen in liquid nitrogen and stored at −80°C until examination. Each intestinal segment was carefully dissected, and the tissue was prepared for analysis.

### 2.5 Serum antioxidant index

The serum activities of catalase (CAT), superoxide dismutase (SOD), glutathione peroxidase (GSH-Px), and malondialdehyde (MDA) were quantitatively determined in Lezhou black ducks at 21 and 42 days using commercial enzyme-linked immunosorbent assay (ELISA) kits as our previous research report (11) (CAT Cat.BC0200, SOD Cat.BC0170, Solarbio Life Sciences, Beijing, China; GSH-Px Cat.A005-1-1, Nanjing Jiancheng Bioengineering Institute, Nanjing, China; MDA Cat.S0131S, Beyotime, Shanghai, China), in accordance with the manufacturer’s protocols.

### 2.6 Complement and immunoglobulin content determination

The levels of complement component 3 (C3), complement component 4 (C4), and immunoglobulin G (IgG) in serum were quantitatively determined at 21- and 42-days post-immunization using ELISA (Duck C3 Cat.QS48326, C4 Cat.QS48328, IgG Cat.QS48162, Beijing Qisong Biotechnology Co., LTD, Beijing, China), with all procedures conducted in accordance with the manufacturer’s protocols.

### 2.7 Serum cytokine content was determined

The serum concentrations of tumor necrosis factor *α* (TNF-α), interferon-*γ* (IFN-γ), interleukin-2 (IL-2), interleukin-4 (IL-4), and interleukin-6 (IL-6) were quantitatively determined using ELISA (Duck TNF-α Cat.QS49067, IFN-γ Cat.QS42217, IL-2 Cat.QS42220, IL-4 Cat.QS42219, IL-6 Cat.QS42215, Beijing Qisong Biotechnology Co., LTD, Beijing, China).

### 2.8 Analysis of gene expression in immune organs

The HiScript III RT SuperMix for qPCR (Vazyme Biotech Co.,Ltd. Nanjing, China), including gDNA wiper, was used for cDNA synthesis from total RNA. The resulting cDNA served as the template for real-time PCR using the ChamQ SYBR qPCR Master Mix (Vazyme Biotech Co.,Ltd. Nanjing, China) as our previous research report (11). *β*-actin was employed as the housekeeping gene for relative quantification of TNF-*α*, IFN-*γ*, IL-2, IL-4, IL-6, SOD, GSH-Px, and CAT. Primers were designed and synthesized by Shanghai Shenggong based on GenBank sequences; their sequences were listed in Table 2.

**Table 2:** Primers utilized in relative real-time polymerase chain reaction analysis.

Genes	Prime sequence(5′ → 3′)	Gene No.
*β*-actin	F:CTTCACCACCACAGCCGAG R:AATCCAGGGCGACATAGCAC	NM_001310421.1
TNF-α	F:GCCACAGAGAAGAAGCAAACC R:CCAGCAGAGAGTTGTCAGGA	XM_027471963.2
IFN-γ	F:AGAGACCTCGTGGAACTGTC R:ACAGCTCACTCACAGCCTT	NM_001310417.1
IL-2	F:AAACCTGGGAACAAGCATGAA R:AGCTGCACTCCTTTGTGTCATT	NM_001310373.1
IL-4	F:ATGAGACAGACACCGACATGG R:TGTCACGATGTGCAGCAAGT	XM_032196612.1
IL-6	F:CAGGAGGAGATGTGCGAGAAGT R:CGAAGCCAGCCAGGAGACA	XM_027450925.2
SOD	F:AGCGACGACCTGGGCAAAG R:GCACTTGGCTATTCCGATGAC	XM_005019265.5
CAT	F:GTGCGTGACTGACAACCAAGG R:CTGAAATACACATGCGGCTCT	XM_027458335.2
GSH-Px	F:CACCAGGAGAATGCCACCAAC R:TCCCGTTCACCTCGCACTTC	XM_027467953.2

The optimized PCR reaction mixture contains 20 μL of reaction solution, composed of 10 μL 2× ChamQ Universal SYBR qPCR Master Mix, 0.4 μL of each forward and reverse primers (10 μM), 1.0 μL cDNA template, and the rest filled with ddH_2_O to reach a total volume of 20 μL. The PCR amplification process starts with a denaturation step at 95°C for 30 s, followed by 40 cycles of denaturation at 95°C for 10 s and annealing at 60°C for 30 s. After amplification, a melting curve analysis was performed from 95°C to 60°C over 60 s, with a final hold at 95°C for 15 s to collect data. The relative expression level of mRNA was calculated using the 2-^ΔΔCt^ method.

### 2.9 Serum barrier factor determination

The serum levels of DAO and D-LA in 42-day-old Leizhou black ducks were quantitatively assayed using a commercial ELISA from Beijing Solarbio Science and Technology Co., Ltd. China, and the results were interpreted in accordance with the manufacturer’s instructions.

### 2.10 Data analysis

SPSS 26.0 was used for one-way ANOVA, followed by Tukey’s HSD *post hoc* test. Statistical significance was set at *p <* 0.05, indicating a significant difference, and *p* < 0.01 denoted an extremely significant difference. Results were expressed as mean ± SD.

To explore the relationship between blood antioxidant, immune indexes and immune organ antioxidant immune indexes, intestinal health indexes of Leizhou black ducks under the influence of *Rhodotorula mucilaginosa* ZTHY2. Spearman’s rank correlation analysis was performed utilizing the OmicStudio tools accessible at https://www.omicstudio.cn/tool. A *p*-value below 0.05 was considered indicative of statistical significance, whereas a trend toward significance was defined as 0.05 ≤ *p <* 0.10.

## 3 Results

### 3.1 The impact of *Rhodotorula mucilaginosa* ZTHY2 on the growth performance of Leizhou black ducks

**Figure 1:**
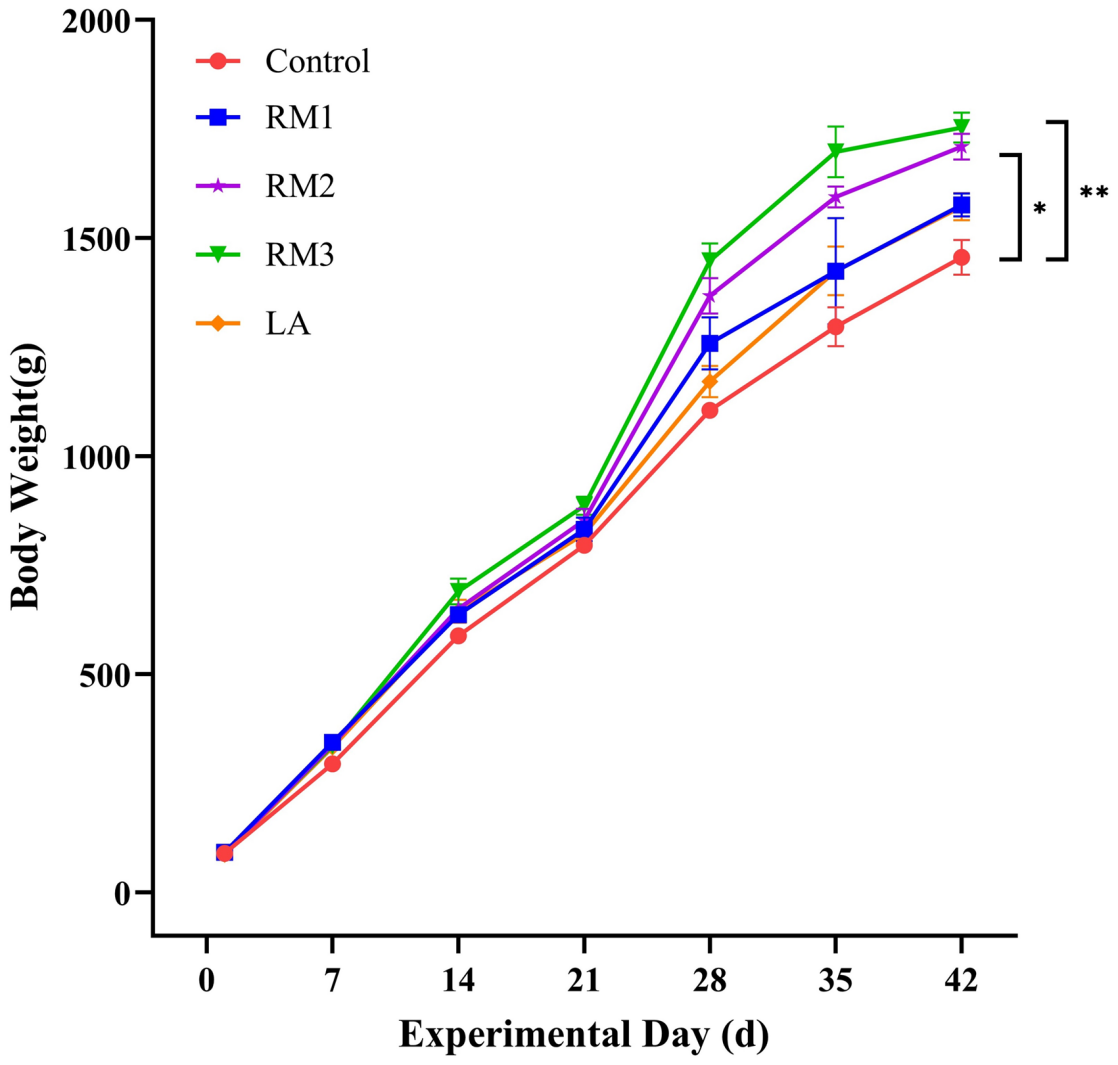
Illustrates the effect of *R. mucilaginosa* ZTHY2 on the average body weight of Leizhou black ducks. Control (Control group), RM1 (2×10^7^ CFU/kg RM group), RM2 (2×10^8^ CFU/kg RM group), RM3 (2 × 10^9^ CFU/kg RM group), LA (2 × 10^9^ CFU/kg LA group).* *p <* 0.05; ** *p <* 0.01.

The growth performance of Leizhou black ducks was assessed following the administration of ZTHY2. At day 42 of the trial, a significant increase in body weight was observed in the Leizhou black ducks supplemented with 2 × 10^8^ CFU/kg and 2 × 10^9^ CFU/kg RM, when compared to the control group (*p <* 0.05 and *p <* 0.01), as depicted in Figure 1.

### 3.2 The modulatory effect of *Rhodotorula mucilaginosa* ZTHY2 on the antioxidant capacity in Leizhou black ducks

**Figure 2:**
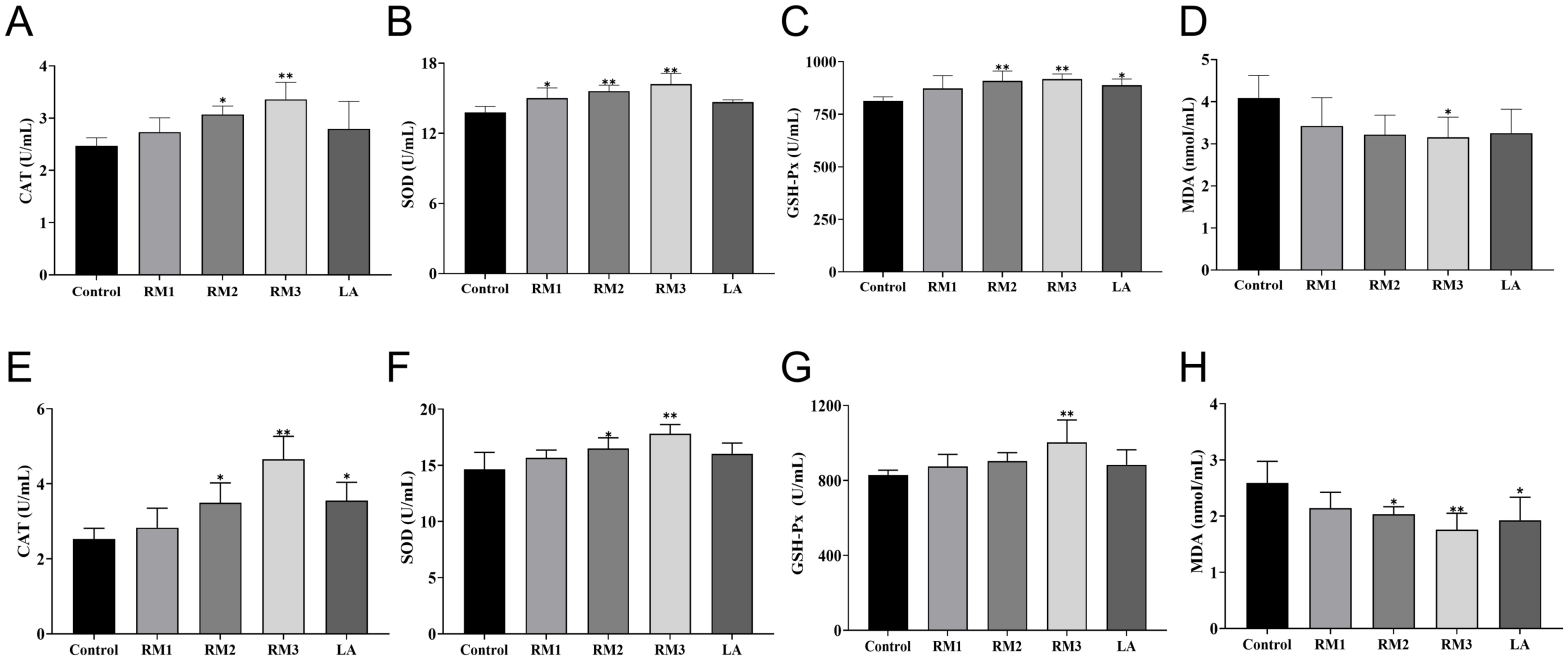
Effect of *R. mucilaginosa* ZTHY2 on the antioxidant function of Leizhou black ducks. The CAT, SOD, GSH-Px and MDA in the serum were detected by the commercial kit. **(A–D)** CAT, SOD, GSH-P and MDA in the serum of Leizhou black ducks at 21 days old. **(E–H)** CAT, SOD, GSH-Px and MDA in the serum of Leizhou black ducks at 42 days old. Compared with control group, *p < 0.05, **p < 0.01.

The study meticulously evaluated the impact of ZTHY2 on the antioxidant capacity of 21 and 42-day-old Leizhou black ducks. Figure 2 outlines the effects of *Rhodotorula mucilaginosa* ZTHY2 on the antioxidant functions of these ducks. By the 21st day (Figures 2A–D), the group supplemented with 2 × 10^7^ CFU/kg of RM showed a significant increase in serum Superoxide Dwas mutase (SOD) activity (*p <* 0.05), while the groups treated with 2 × 10^8^ CFU/kg and 2 × 10^9^ CFU/kg of RM demonstrated enhanced serum Catalase (CAT) activity (*p <* 0.05 or *p <* 0.01), respectively. Moreover, the groups treated with 2 × 10^8^ CFU/kg and 2 × 10^9^ CFU/kg of RM significantly elevated both Glutathione Peroxidase (GSH-Px) and SOD activities (*p <* 0.01) compared to the control group. Additionally, at 21 days, the group was supplemented with 2 × 10^9^ CFU/kg of RM showed a significant reduction in serum Malondialdehyde (MDA) content (*p <* 0.05), whereas the group treated with 2 × 10^9^ CFU/kg of LA showed a significant increase in serum GSH-Px activity (*p <* 0.05) compared to the controls.

At the 42-day (Figures 2E–H), treatment with a dosage of 2 × 10^9^ CFU/kg RM markedly enhanced the serum activities of catalase (CAT), superoxide dis-mutase (SOD), and glutathione peroxidase (GSH-Px), while significantly reducing malondialdehyde (MDA) levels (*p <* 0.01) when compared to the control group. Moreover, the groups administered with 2 × 10^8^ CFU/kg RM and 2 × 10^9^ CFU/kg LA exhibited significant elevations in CAT activity and reductions in MDA levels (*p <* 0.05). Additionally, SOD activity was significantly increased (*p <* 0.05) in the group treated with 2 × 10^8^ CFU/kg RM.

### 3.3 The modulatory impact of *Rhodotorula mucilaginosa* ZTHY2 on the complement system and immunoglobulin levels in Leizhou black ducks

**Figure 3:**
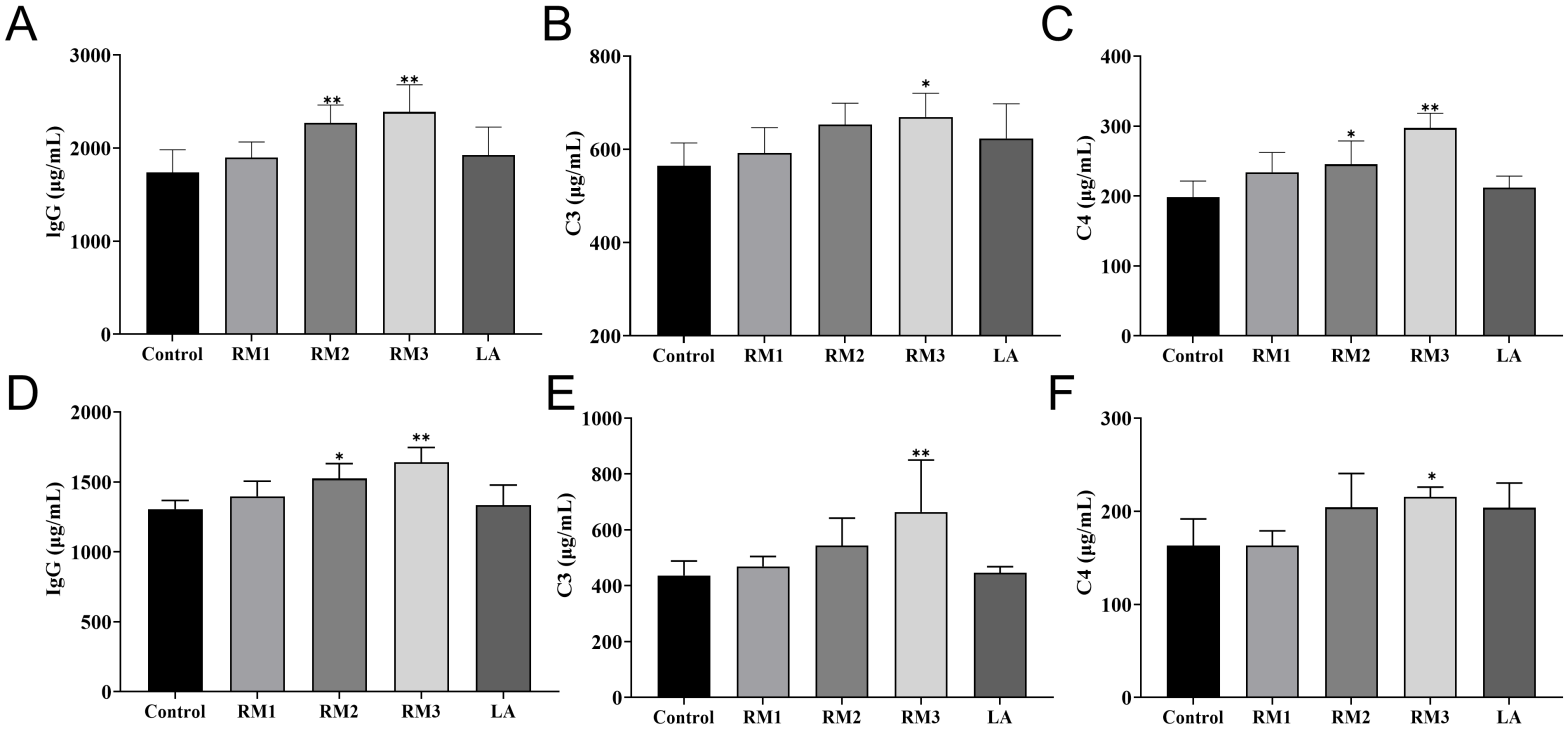
Effect of *R.mucilaginosa* ZTHY2 on complement and immunoglobulin levels of Leizhou black ducks. The IgG, C3 and C4 contents in the serum of Leizhou black ducks were detected by ELISA. **(A–C)** The IgG, C3 and C4 contents in the serum of Leizhou black ducks at 21 days old. **(D–F)** The IgG, C3 and C4 contents in the serum of Leizhou black ducks at 42 days old. Compared with control group, *p < 0.05, **p < 0.01.

The study evaluated the impact of ZTHY2 on complement and immunoglobulin levels in Leizhou black ducks. Figure 3 shows that supplementing with 2 × 10^8^ CFU/kg of the yeast significantly increased C4 levels compared to the control (*p <* 0.05). At day 21(Figures 3A–C), the 2 × 10^9^ CFU/kg dose elevated C3 and C4 serum levels (*p <* 0.05 or *p <* 0.01), and both doses increased IgG levels at day 21 (*p <* 0.01). Furthermore, at day 42(Figures 3D–F), the 2 × 10^9^ CFU/kg yeast raised C3 and C4 levels compared to the control (*p <* 0.05 or *p <* 0.01), while IgG levels were upregulated in both RM groups at day 42 (*p <* 0.05 or *p <* 0.01).

### 3.4 Effect of *Rhodotorula mucilaginosa* ZTHY2 on serum immune function of Leizhou black ducks

**Figure 4:**
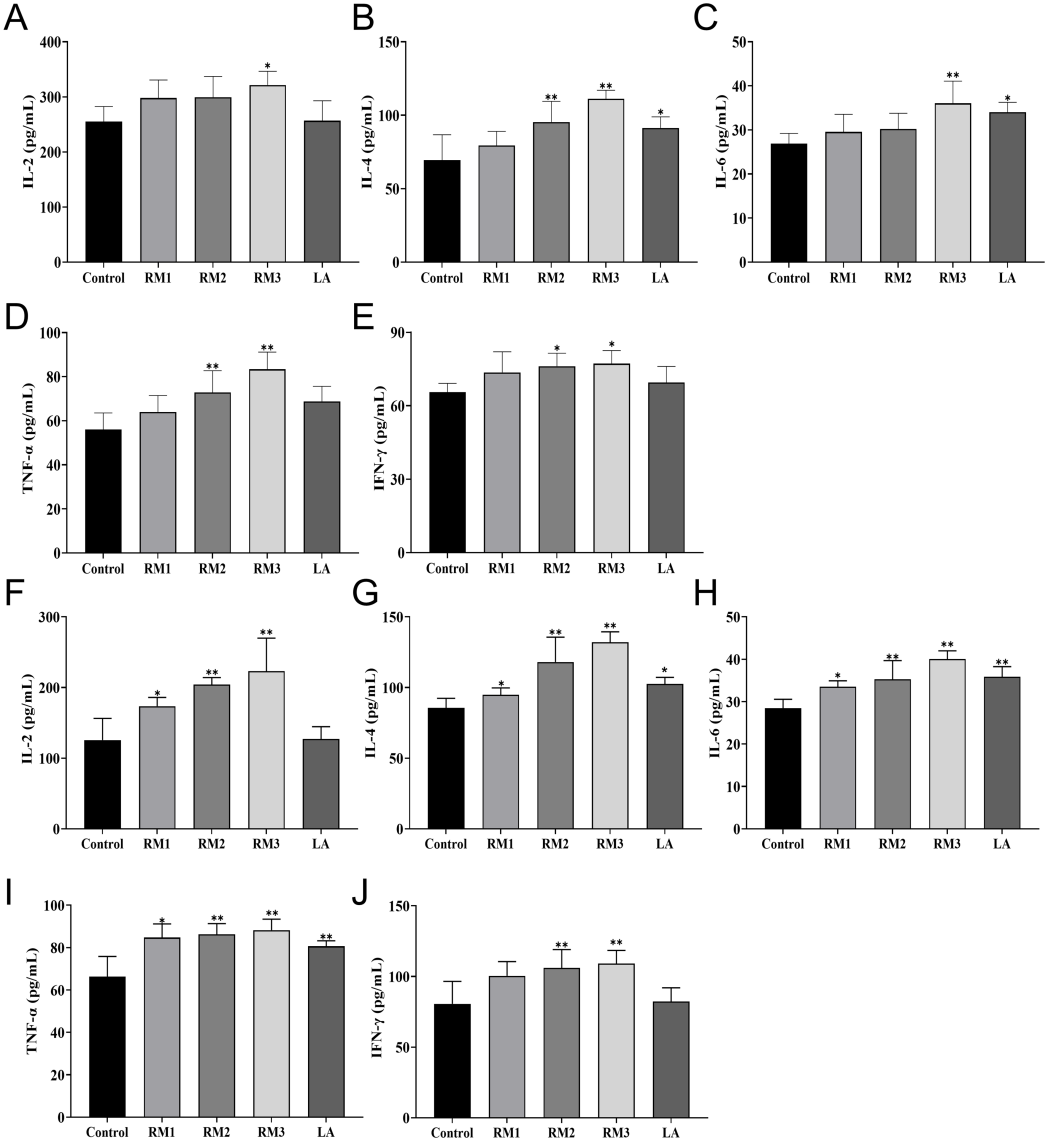
Effect of *R.mucilaginosa* ZTHY2 on serum immune function of Leizhou black ducks. The IL-2, IL-4, IL-6, TNF-*α* and IFN-*γ* contents in the serum of Leizhou black ducks were detected by ELISA. **(A–E)** The IL-2, IL-4, IL-6, TNF-α and IFN-γ contents in the serum of Leizhou black ducks at 21 days old. **(F–J)** The IL-2, IL-4, IL-6, TNF-α and IFN-γ contents in the serum of Leizhou black ducks at 42 days old. Compared with control group, *p < 0.05, **p < 0.01.

Figure 4 first illustrated the impact of ZTHY2 on the serum immune function in 21-day-old Leizhou black ducks (Figures 4A–E). ZTHY2 exhibits incremental effects on the serum levels of IL-2, IL-4, IL-6, TNF-*α*, and IFN-*γ*. Notably, a dosage of 2 × 10^8^ CFU/kg significantly increases (*p <* 0.05) the serum IFN-γ level in the RM group compared to the control, with highly significant (*p <* 0.01) boosts in IL-4 and TNF-*α* levels. In the RM group treated with 2 × 10^9^ CFU/kg, both IL-2 and IFN-*γ* levels were significantly elevated (*p <* 0.05) alongside highly significant (*p <* 0.01) increases in IL-4, IL-6, and TNF-*α* levels. Additionally, the LA group treated with 2 × 10^9^ CFU/kg shows a significant (*p <* 0.05) rise in serum IL-6 levels compared to the control.

The study evaluated the influence of ZTHY2 on the immune system of 42-day-old Leizhou black ducks (Figures 4F–J). Figure 4 second showed that compared to the control, 2 × 10^7^ CFU/kg of the yeast significantly raised serum levels of IL-2, IL-4, IL-6, and TNF-*α* at day 42 (*p <* 0.05). Moreover, 2 × 10^8^ CFU/kg and 2 × 10^9^ CFU/kg of ZTHY2 markedly increased IL-2, IL-4, IL-6, TNF-α, and IFN-*γ* (*p <* 0.01), with the 2 × 10^9^ CFU/kg group showing significantly higher IL-4, IL-6, and TNF-α concentrations than the control (*p <* 0.05 or *p <* 0.01).

### 3.5 The modulatory effect of *Rhodotorula mucilaginosa* ZTHY2 on the expression levels of cytokines in the immune organs of Leizhou black ducks

**Figure 5:**
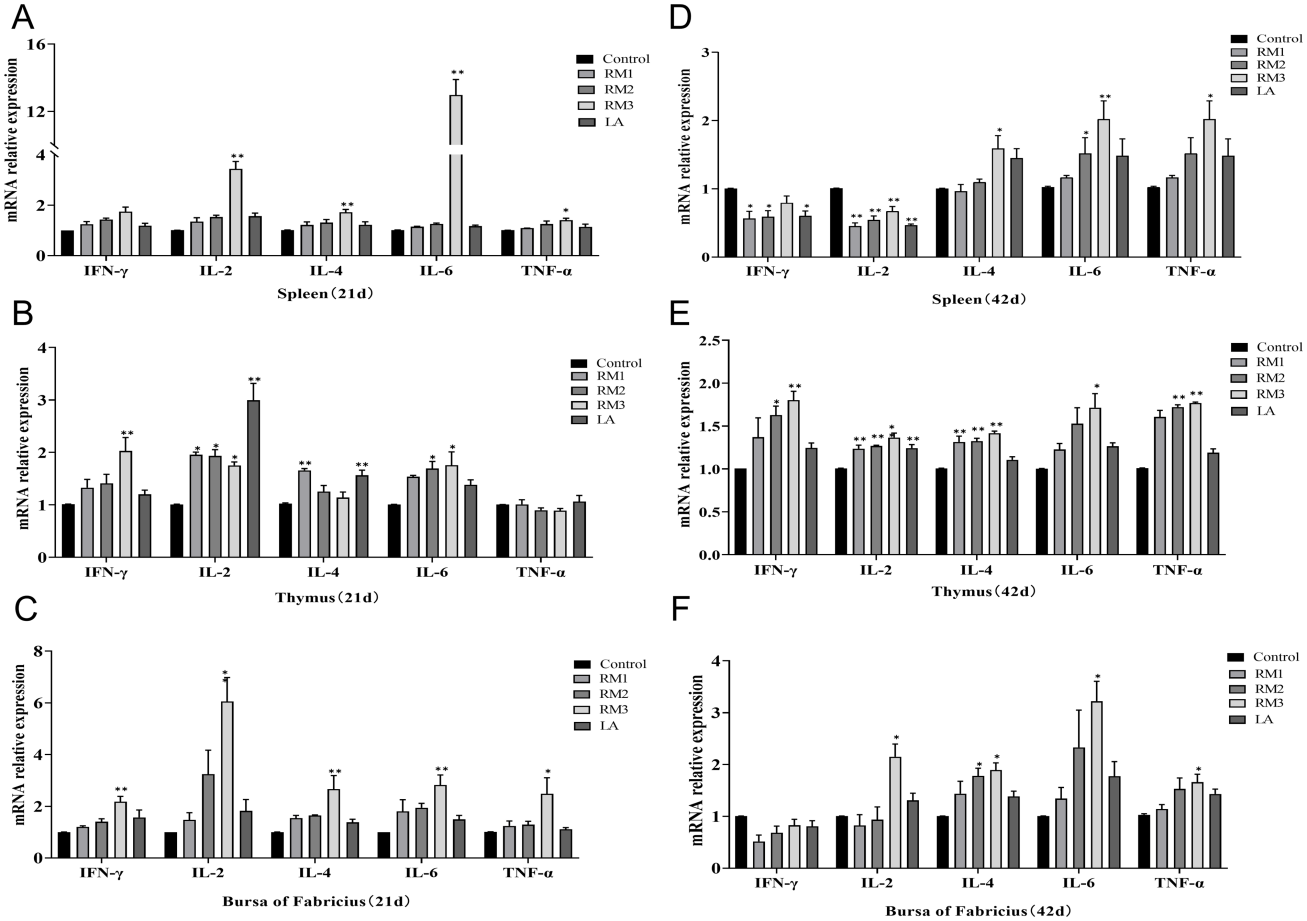
Effect of *R. mucilaginosa* ZTHY2 on expression levels of cytokines in the immune organs of Leizhou black ducks. The IL-2, IL-4, IL-6, TNF-α and IFN-γ contents in the spleen, thymus and bursa were detected by ELISA. **(A–C)** The IL-2, IL-4, IL-6, TNF-α and IFN-γ contents in the spleen, thymus and bursa of Leizhou black ducks at 21 days old. **(D–F)** The IL-2, IL-4, IL-6, TNF-α and IFN-γ contents in the spleen, thymus and bursa of Leizhou black ducks at 42 days old. Compared with control group, *p < 0.05, **p < 0.01.

The modulatory effects of ZTHY2 on the cytokine expression profiles in the immune organs of 21-day-old Leizhou black ducks were detailed in Figures 5A–C. Compared to the control group, the 2 × 10^9^ CFU/kg RM group exhibited a significant increase in TNF-*α* mRNA expression (*p <* 0.05) and remarkably higher levels of IFN-*γ*, IL-2, IL-4, and IL-6 mRNA expression (*p <* 0.01) in spleen (Figure 5A). The thymus showed significantly increased expression levels of IL-2 and IL-4 mRNA in the 2 × 10^7 CFU/kg RM group at day 21 (*p <* 0.05 or *p <* 0.01), while the 2 × 10^8^ CFU/kg RM group demonstrated significantly elevated expression levels of IL-2 and IL-6 mRNA at day 21 (*p <* 0.05). In addition, the expressions of IFN-*γ*, IL-2, and IL-6 mRNA were significantly upregulated in the thymus of the 2 × 10^9CFU/kg RM group (*p <* 0.01 or *p <* 0.05), as well as in the thymus of the 2 × 10^9^ CFU/kg LA group for both IL-2 and IL-6 mRNA (*p <* 0.01; Figure 5B). Furthermore, there was an upregulation observed in bursa with regards to IFN-γ, IL-2, IL-4, IL-6 and TNF-*α* mRNAs expressions in 2 × 10^9^ CFU/kg RM group at 21 days (*p <* 0.05 or *p <* 0.01; Figure 5C).

The modulatory effects of ZTHY2 on the cytokine expression profiles in the immune organs of 42-day-old Leizhou black ducks were detailed in Figures 5D–F. As shown in Figure 5, compared with the control group, the expression levels of IFN-*γ* and IL-2 mRNA in spleen of 2 × 10^8^ CFU/kg and 2 × 10^7^ CFU/kg RM groups were down-regulated for 42 days (*p <* 0.05 or *p <* 0.01). The 2 × 10^8 CFU/kg RM group significantly increased the expression of IL-6 mRNA on 42 days (*p <* 0.05), while the 2 × 10^9^ CFU/kg RM group and 2 × 10^9^ CFU/kg LA group significantly decreased the expression of IL-6 mRNA on 42 days (*p <* 0.05; Figure 5D). At the same time, the mRNA expression levels of IL-4, IL-6 and TNF-*α* were up-regulated in the 2 × 10^9^ CFU/kg RM group (*p <* 0.05 or *p <* 0.01). The expression of IL-2 and IL-4 mRNA in thymus of 2 × 10^7^ CFU/kg RM group was significantly increased on day 42 (*p <* 0.01). The expression levels of IFN-γ, IL-2, IL-4 and TNF-α mRNA in thymus of 2 × 10^8^ CFU/kg RM group were up-regulated for 42 days (*p <* 0.05 or *p <* 0.01). The expression of IFN-γ, IL-2, IL-4, IL-6 and TNF-α mRNA in thymus of 2 × 10^9^ CFU/kg RM group increased for 42 days (*p <* 0.05 or *p <* 0.01; Figure 5E). The 2 × 10^8^ CFU/kg RM group significantly increased the mRNA expression of IL-4 in the bursa for 42 days (*p <* 0.05), and the 2 × 10^9^ CFU/kg RM group significantly increased the mRNA expression of IL-2, IL-4, IL-6, and TNF-α (*p <* 0.05 or *p <* 0.01; Figure 5F).

### 3.6 The influence of *Rhodotorula mucilaginosa* ZTHY2 on the mRNA expression levels of CAT, SOD, and GSH-Px in the immune organs of Leizhou black ducks

**Figure 6:**
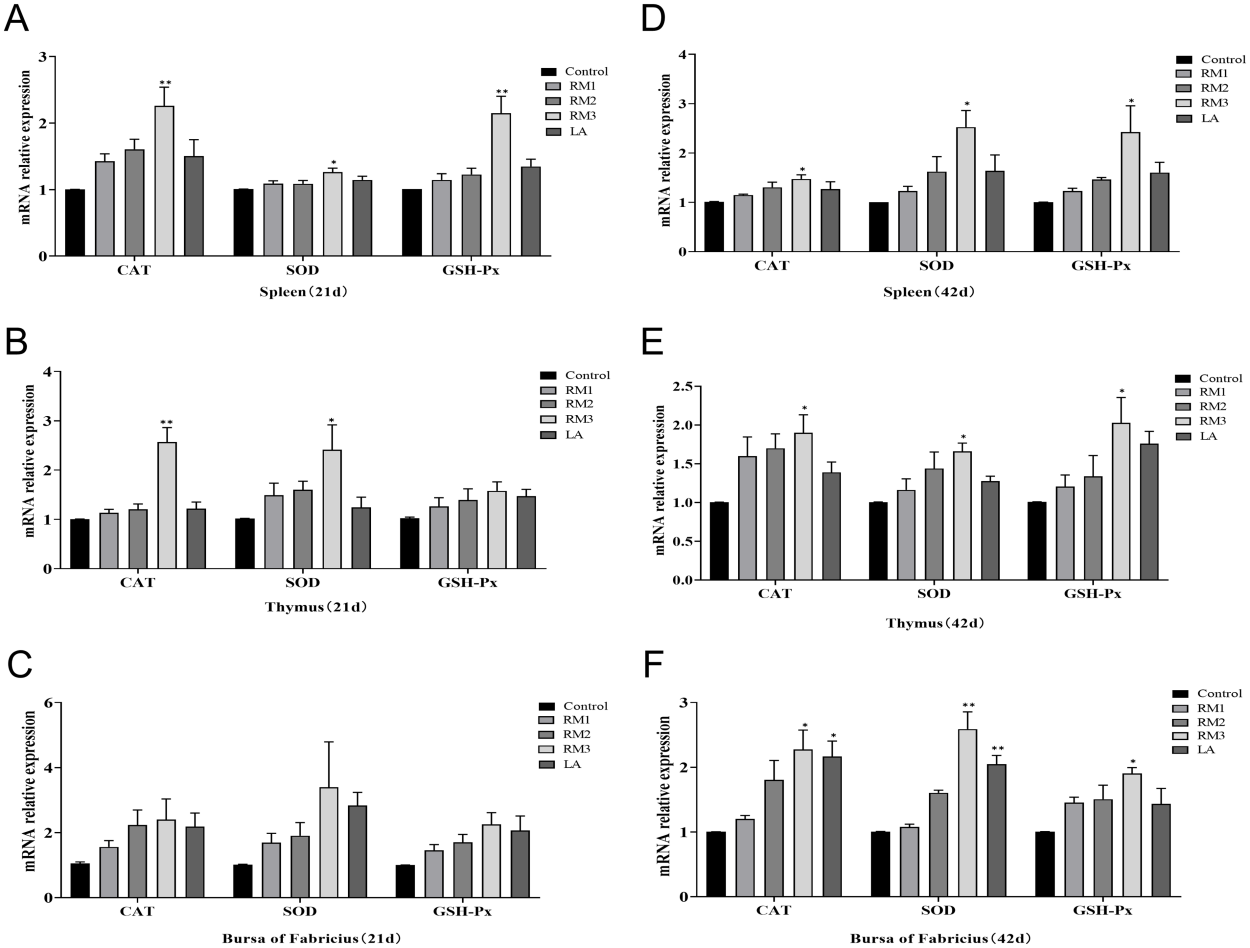
Effect of *R. mucilaginosa* ZTHY2 on CAT, SOD, GSH-Px mRNA expression levels in immune organs of Leizhou black ducks. The gene expressions of CAT, SOD and GSH-Px in the spleen, thymus and bursa were detected by qPCR. **(A–C)** The gene expressions of CAT, SOD and GSH-Px in the spleen, thymus and bursa were detected of Leizhou black ducks at 21 days old. **(D–F)** The gene expressions of CAT, SOD and GSH-Px in the spleen, thymus and bursa were detected of Leizhou black ducks at 42 days old. Compared with control group, *p < 0.05, **p < 0.01.

Figure 6 was playing the impact of ZTHY2 on the mRNA expression of antioxidant enzymes CAT, SOD, and GSH-Px in the immune organs of 21-day-old Leizhou black ducks (Figures 6A–C). The 2 × 10^9^ CFU/kg RM group exhibited a significant upregulation of CAT, SOD, and GSH-Px mRNA in the spleen at 21 days (Figure 6A), and also showed increased expression of CAT and SOD in the thymus at the same timepoint (*p <* 0.05 or *p <* 0.01; Figure 6B). Additionally, a trend of increased mRNA expression for CAT, SOD, and GSH-Px was observed in the Bursa after ZTHY2 treatment at 21 days, though these differences were not significant (*p* > 0.05; Figure 6C).

It was illustrated the significant modulatory effects of 2 × 10^9^ CFU/kg of Gum Red Yeast ZTHY2 on the mRNA expression of antioxidant enzymes Catalase (CAT), Super-oxide Dismutase (SOD), and Glutathione Peroxidase (GSH-Px) in the immune organs of 42-day-old Leizhou black ducks in Figures 6D–F. The yeast significantly elevated mRNA levels of these enzymes in the spleen, thymus, and bursa at day 42, reaching statistical significance at *p <* 0.05 or *p <* 0.01 (Figures 6D–F). Notably, a specific upregulation of CAT and SOD mRNA expression in the bursa was observed in the group supplemented with 2 × 10^9^ CFU/kg of ZTHY2 at day 42 (*p <* 0.05 or *p <* 0.01; Figure 6F).

### 3.7 The influence of *Rhodotorula mucilaginosa* ZTHY2 on the serum DAO and D-LA concentrations in Leizhou black ducks

**Figure 7:**
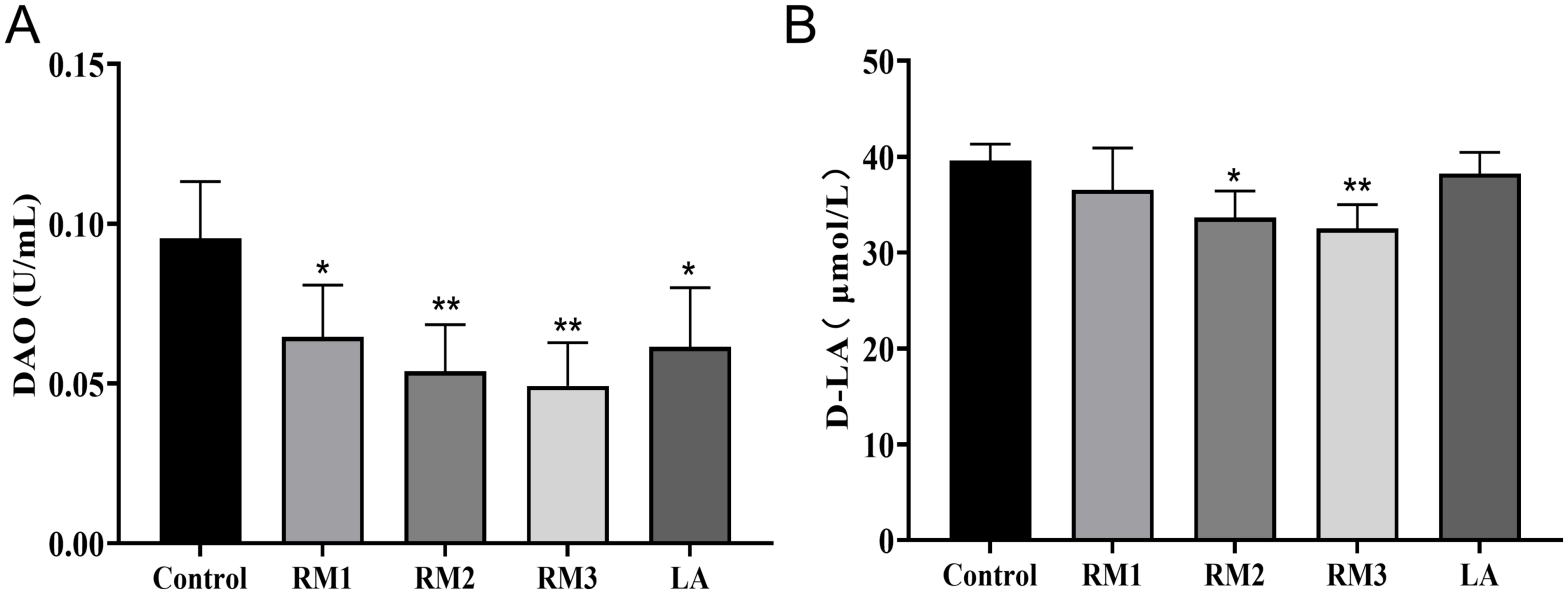
The influence of *Rhodotorula mucilaginosa* ZTHY2 on the serum DAO and D-LA concentrations in Leizhou black ducks. The content of DAO and D-LA in serum of Leizhou black ducks were detected by commercial kit. **(A)** The content of DAO in serum of Leizhou black ducks at 42 days old. **(B)** The content of D-LA in serum of Leizhou black ducks at 42 days old. Compared with control group, *p < 0.05, **p < 0.01.

D-lactic acid (D-LA) and diamine oxidase (DAO) as sensitive biomarkers reflecting alterations in intestinal mucosal permeability and epithelial barrier function. Figure 7 illustrated the impact of ZTHY2 on Leizhou black duck serum diamine oxidase (DAO) and D-lactic acid (D-LA) levels. Dietary inclusion of ZTHY2 significantly decreased DAO and D-LA concentrations compared to the control (*p <* 0.05 or *p <* 0.01), with the most beneficial effects observed at a dosage of 2 × 10^9^ CFU/kg.

### 3.8 Correlation analysis of blood antioxidant, immune indexes and immune organ antioxidant immune indexes, intestinal health indexes of Leizhou black ducks under the influence of *Rhodotorula mucilaginosa* ZTHY2

**Figure 8:**
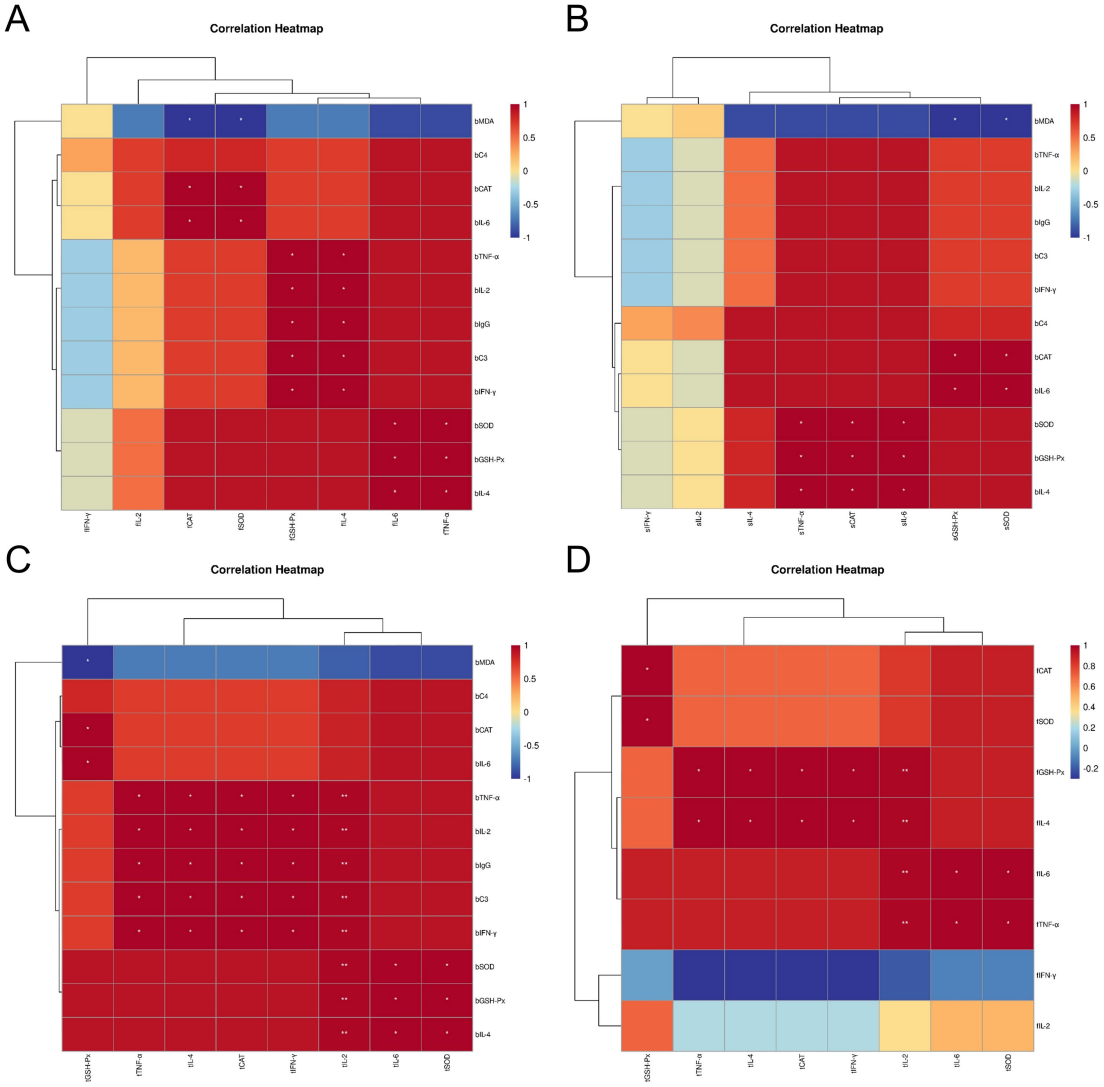
Spearman correlation analysis between the blood antioxidant and immune indexes and immune organ antioxidant and immune indexes of Leizhou black ducks under the action of ZTHY2. **(A)** Blood and bursa; **(B)** Blood and spleen; **(C)** Blood and thymus; **(D)** thymus and bursa.

After subjecting Leizhou black ducks to ZTHY2, we conducted a Spearman correlation analysis on their blood antioxidant and immune indices, as well as the antioxidant and immune indices of their immune organs. The results showed positive correlations between blood antioxidant enzymes (CAT, GSH-Px, SOD) and bursal antioxidant enzymes (CAT, SOD, IL-6, TNF-*α*) in the bursa (*p* < 0.05), while MDA was inversely associated with these enzymes (*p* < 0.05). Beyond C4, various blood immune indices were positively associated with bursal antioxidant enzymes (CAT, SOD, GSH-Px) and cytokines (IL-4, IL-6, TNF-*α*) (*p* < 0.05), as shown in Figure 8A. In the spleen, blood antioxidant enzymes were positively correlated with spleen enzymes and cytokines (IL-6, TNF-α) (p < 0.05), and MDA was negatively correlated with GSH-Px and SOD (*p* < 0.05), as shown in Figure 8B. In the thymus, blood antioxidant indices were positively correlated with thymic indices (GSH-Px, SOD, IL-6, IL-2) (*p* < 0.05, IL-2 significantly at *p* < 0.01), while MDA was negatively correlated with GSH-Px (*p* < 0.05), as illustrated in Figure 8C. A Spearman correlation analysis on the antioxidant and immune indices of the thymus and bursa revealed that all other indices in the bursa were positively correlated with those in the thymus, except for IL-2 and IFN-*γ* (*p* < 0.05, IL-2 significantly at *p* < 0.01), as depicted in Figure 8D.

**Figure 9:**
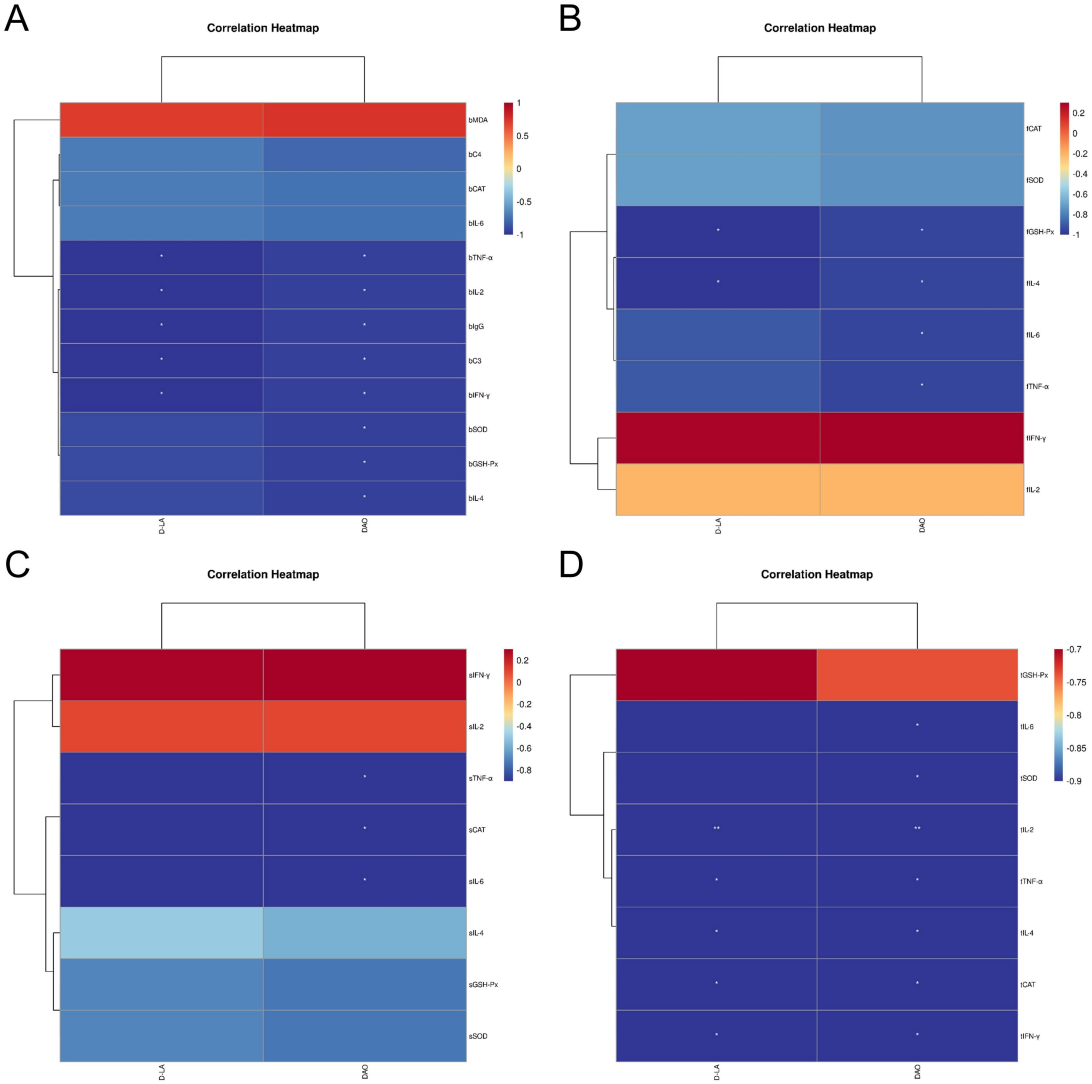
Spearman correlation analysis between the blood, immune organ antioxidant, immune indexes and intestinal health indexes (DAO and D-LA, as D.D.) of Leizhou black ducks under the action of ZTHY2. **(A)** Blood and D.D.; **(B)** Bursa and D.D.; **(C)** spleen and D.D.; **(D)** thymus and D.D.

Spearman correlation analysis revealed negative associations between Leizhou black duck blood parameters, antioxidant and immune indices, and intestinal health markers after treatment with ZTHY2. DAO and D-LA negatively correlated with GSH-Px, SOD, TNF-α, IL-2, IgG, C3, IFN-γ, and IL-4 in blood (*p <* 0.05; Figure 9A). In the bursa, DAO and D-LA were inversely related to GSH-Px, IL-4, IL-6, and TNF-α (*p <* 0.05; Figure 9B). In the spleen, only DAO was associated with TNF-α and IL-6 negatively (*p <* 0.05; Figure 9C). Within the thymus, DAO and D-LA generally exhibited negative correlations with intestinal health markers, except for GSH-Px (*p <* 0.05), particularly with IL-2 (*p <* 0.01; Figure 9D).

## 4 Discussion

### 4.1 The impact of *Rhodotorula mucilaginosa* ZTHY2 on the growth performance of Leizhou black ducks

*Rhodosaccharomyces colloides*, which is rich in cellular proteins, small molecular peptides, vitamins, and unsaturated fatty acids, is easily absorbed and utilized by animals, directly participating in their metabolic processes (14). One of the key benefits is the natural flavor it brings. It contains nucleotides that release aromatic compounds and amino acids that contribute to a rich umami taste. These elements work together harmoniously to significantly enhance the feed’s flavor, thus stimulating the animals’ appetite (12, 24). This natural flavor enhancement not only makes the feed more appealing to the animals but also encourages them to eat more actively, which is vital for their overall health and growth efficiency.

In our study, we specifically examined the effects of *Rhodotorula mucilaginosa* ZTHY2 on the growth performance of Leizhou black ducks. During the experiment, we added 2 × 10^8^ CFU/kg and 2 × 10^9^ CFU/kg of ZTHY2 to the feed, respectively. The results showed that, compared to the control group, the Leizhou black ducks supplemented with ZTHY2 experienced a notable increase in body weight. This weight gain can be attributed to the fact that the ducklings’ thermoregulatory and digestive systems are not fully developed at the beginning of their growth, making additional nutritional support especially important (25). Based on these findings, we can conclude that adding ZTHY2 to the feed significantly improves the growth performance of Leizhou black ducks. This additive not only provides essential nutrients but also enhances the feed’s palatability with its unique flavor, thereby boosting the animals’ appetite and growth. These results provide a strong scientific basis for the use of *Rhodotorula mucilaginosa* ZTHY2 in animal nutrition and offer valuable insights for future feeding practices.

### 4.2 *Rhodotorula mucilaginosa* ZTHY2 strain could enhance the antioxidant capacity of Leizhou black ducks

In healthy animals, a delicate balance was maintained between antioxidant enzymes and reactive oxygen species (ROS). However, during livestock and poultry breeding, the utilization of inappropriate feed additives or the repercussions of environmental stress could unsettle the equilibrium of the oxidation/antioxidant systems, culminating in the excessive aggregation of ROS and the induction of oxidative stress (26). An increased concentration of ROS could lead to the degradation of cellular membrane structure and function, triggering lipid peroxidation, protein oxidation, and DNA damage (27). Antioxidant enzymes, such as superoxide dismutase (SOD), glutathione peroxidase (GSH-Px), and catalase (CAT), were capable of neutralizing excessive ROS, reducing the production of malondialdehyde (MDA), a byproduct of membrane lipid peroxidation, thereby alleviating oxidative stress and maintaining the homeostasis of the oxidation/antioxidant systems *in vivo* (28).

Throughout growth, *Rhodoyeast* could produce carotenoids like astaxanthin, exopolysaccharides, and other metabolites, which participate in the regulation of various oxidoreductase-related metabolic pathways (13). These natural antioxidants potentiate their antioxidant efficacy by promoting the nuclear translocation of nuclear factor erythroid 2-related factor 2 (Nrf2), modulating the expression of downstream antioxidant enzyme genes, and scavenging oxygen free radicals within cells (14, 15, 29). Nrf2, a member of the basic leucine zipper transcription factor family, plays a key role in regulation of cellular redox homeostasis by inducing various detoxifying and antioxidant enzymes, such as the oxidative stress marker malondialdehyde (MDA), and the antioxidant enzyme superoxide dismutase (SOD), catalase (CAT), glutathione peroxidase (GPX), and glutathione reductase (GR) (30). Hu et al. reported that weaning piglets fed daily with 3 × 10^10^ CFU of red yeast exhibited a significant enhancement in the activities of serum antioxidant enzymes, including total antioxidant capacity (T-AOC) and GSH-Px, along with a reduction in MDA content (24).The study showed that incorporating ZTHY2 into the diet of Leizhou Black ducks enhanced their serum antioxidant activities, including catalase (CAT), glutathione peroxidase (GSH-Px), and superoxide dismutase (SOD) on days 21 and 42. This was accompanied by a decrease in malondialdehyde (MDA) content in a dose-dependent manner.

Quantitative polymerase chain reaction (qPCR) confirmed that yeast supplementation upregulated the mRNA expression of antioxidant enzymes in the spleen, thymus, and bursa at both 21 and 42 days, with the strongest effects at a supplemental level of 2 × 10^9^ CFU/kg. These findings indicate that ZTHY2 inhibits lipid peroxide production by improving serum antioxidant activities and increasing the expression of antioxidant-related genes, thereby strengthening antioxidant capacity and maintaining redox balance. Furthermore, increased antioxidant capacity within the body reduces oxidative damage to proteins and lipids in muscle tissue, while improved antioxidant enzyme activity had a positive impact on meat quality (31). This evidence reinforces the idea that ZTHY2 could improve the muscle quality and nutritional value of Leizhou Black ducks.

### 4.3 *Rhodotorula mucilaginosa* ZTHY2 strain had been shown to enhance the humoral immune response of Leizhou black ducks

IgG, C3 and C4 were the key parameters for assessing humoral immunity, working in concert to bolster the avian immune response and enhance disease resistance. Research indicates that the inclusion of 0.10% yeast nucleotides or yeast *β*-glucan in the diet could elevate the levels of C3, C4, and IgG in serum, thereby enhancing both cellular and humoral immune functions (32). Additionally, doses of 2 × 10^8^ CFU/kg and 2 × 10^9^ CFU/kg of ZTHY2 elevated the serum IgG concentrations at 42-day intervals. IgG constitutes the predominant immunoglobulin present in avian serum, being produced through the stimulation and differentiation of B cells. It possesses a specific binding capacity for pathogenic microorganisms, thereby offering a protective barrier against infections and was crucial for the augmentation of the humoral immune response in birds (33).

The complement system serves as an integral linkage between the adaptive and innate immune responses, with complement components C3 and C4, which were also integral parts of immunoglobulins, aiding antibodies and phagocytes in the host’s defense mechanisms against pathogens. These components were pivotal in immune regulation (34). C3 was the most copious component within the complement system and was pivotal in both the classical and alternative activation pathways, whereas C4 participates in the early stages of the immune response, bolstering subsequent reactions and exhibiting various non-specific immune functions, such as the prevention of immune complex deposition, enhancing phagocytosis, and toxin neutralization (35, 36).

Our experiment found that supplementing with 2 × 10^9^ CFU/kg of ZTHY2 markedly increased the serum levels of C3 and C4 in 42-day Leizhou black ducks. These results indicate that ZTHY2 could stimulate the production of immunoglobulins and complement in the serum of Leizhou black ducks, thereby enhancing humoral immunity and disease resistance in livestock and poultry. The most effective dose was found to be 2 × 10^9^ CFU/kg ZTHY2 added to the feed.

### 4.4 *Rhodotorula mucilaginosa* ZTHY2 strain had been shown to enhance the organ immune function of Leizhou black ducks

Our prior research had demonstrated that ZTHY2 could significantly augment the proliferation of T and B lymphocytes in immunosuppressed mice, concomitant with an increase in the serum secretion of IL-2, IL-4, IL-6, TNF-*α*, IFN-*γ*, and immunoglobulins, thereby significantly enhancing the immune function in mice (11). The immune organs of avian species predominantly encompass the spleen, thymus, and bursa, which serve as the structural underpinnings of the avian immune response and were critical venues for the differentiation and maturation of immune cells (37). The bursa, an exclusive immune organ in birds, was pivotal in the formative stages of B cells, facilitating their early development, proliferation, and the expression of immunoglobulins. The thymus was the anatomical site where T cells originate, proliferate, differentiate, and achieve maturity, functioning as a pivotal barrier and regulatory entity in both cellular and innate immunity. The spleen was capable of manufacturing a spectrum of immune factors that modulate the body’s immune homeostasis, and it executes specific immune functions through the mediation of both T-cell and B-cell-driven humoral and cellular immunity, thereby providing a defense mechanism against microbial incursions and offering direct resistance to extrinsic infections (38–40).

Th2 cell-derived interleukins, including IL-4 and IL-6, significantly contribute to humoral immunity by fostering B cell proliferation and differentiation, aiding in the secretion of immunoglobulins, and exerting multifaceted regulation over immune activity. The intricate interplay and collaboration between Th1 and Th2 cytokines establish a complex cytokine network that mutually maintains the foundations of immune homeostasis (41, 42). Interleukin-2 (IL-2), tumor necrosis factor-alpha (TNF-*α*), and interferon-gamma (IFN-*γ*) represent pivotal Th1 cytokines instrumental in macrophage activation and the orchestration of cellular immune responses. IL-2 and IFN-γ particularly emerge as the principal elements within the IL-2-IFN-γ-NKC immune regulatory network, collaboratively activating cellular immunity and promoting tumor cell apoptosis (43). TNF-α, a critical mediator in the inflammatory response, effectively activates neutrophils and lymphocytes while stimulating the secretion of interleukin-6 (IL-6), thereby enhancing the immune system’s defense mechanisms against pathogenic infections (44).

By day 42, ZTHY2 modulated mRNA expression of these cytokines in the spleen, decreasing IFN-γ and IL-2 expression and increasing TNF-α and IL-4 expression. Conversely, in the thymus and bursa of Farsi, ZTHY2 induced a variable up-regulation of cytokine expression. These results indicate that ZTHY2 regulates cytokine expression within immune organs, maintaining the balance of Th1/Th2 cell populations. This promotes antagonistic and synergistic interactions, enhancing the body’s overall immune function. The mechanism may involve the enrichment of ZTHY2 with metabolically active components such as mannooligosaccharides, *β*-glucan, and nucleotides. These stimulate the complement cascade, activate and proliferate B lymphocytes, and facilitate antibody production, including immunoglobulins (45). Conversely, the augmentation of T-cell proliferation and macrophage activation instigates these immune cells to recruit and secrete a diverse array of cytokines, thereby exerting regulatory functions within the immune response (46). Other yeast studies have also found that they could activate the TLR4-NF-κB signaling pathway (16) or TLR4-NF-κB signaling pathway and enhance macrophage function (17). Liver is an important metabolic organ and immune organ. Mycotoxins in various feeds and feed materials can induce liver inflammation and affect body homeostasis and health (47). Transcriptional co-activator with PDZ-binding motif (TAZ), one of core modules of the Hippo pathway, involves inflammatory cell infiltration in the liver, and might ameliorate the inflammatory response through the Nrf2-reactive oxygen species (ROS)-nuclear factor κB (NF-κB) pathway (30). The above pathways and their key regulatory genes can be used as potential research directions, in the future to explore the regulation of oxidation-inflammation balance by *Rhodotorula mucilaginosa* ZTHY2 through the “gut-liver” axis.

In summary, ZTHY2 enhances immune function by promoting the growth and maturation of T and B cells, orchestrating both cell-mediated and humoral responses, and regulating the release of immune regulators along with the upregulation of immune-related genes, thus maintaining immune balance and improving immune efficacy.

### 4.5 *Rhodotorula mucilaginosa* ZTHY2 had the potential to enhance the intestinal permeability in Leizhou black ducks

D-lactic acid (D-LA), an organic acid derived from the metabolism of intestinal microbiota, and diamine oxidase (DAO), an intracellular enzyme expressed in epithelial cells of the intestinal tract, function as sensitive biomarkers reflecting alterations in intestinal mucosal permeability and epithelial barrier function (48). Under normal physiological conditions, serum levels of D-LA and DAO were typically low. However, disruptions in the equilibrium of gut microbiota or intestinal mucosal injury, which result in increased permeability, frequently lead to elevated serum concentrations of DAO and D-LA. In the current study, the administration of ZTHY2 was found to significantly decrease serum levels of DAO and D-LA in Leizhou black ducks, thereby exerting a protective effect on the intestinal mucosa and preserving its integrity.

## 5 Conclusion

*Rhodotorula mucilaginosa* ZTHY2 could enhance the body’s antioxidant capacity, reduce lipid oxidation damage, resist the occurrence of stress reactions, thereby maintaining immune homeostasis and improving immune function. It also had potential to enhance intestinal barrier function and improve digestive absorption in the intestines. The most efficacious concentration of ZTHY2 had been determined to be 2 × 10^9^ CFU/kg. This study aligns with the forward-thinking trend of “feed without resistance,” encompassing significant implications for the healthful and sustainable advancement of the aquaculture industry.

## Data Availability

The original contributions presented in the study are included in the article/Supplementary material, further inquiries can be directed to the corresponding author/s.
