# Supplementary figures and images for: The active roles of Rhodotorula mucilaginosa ZTHY2 in regulating antioxidant capacity and immune function of Leizhou black ducks
Source: Front Vet Sci. 2025 Jan 30;12:1494892. doi: 10.3389/fvets.2025.1494892 (PMC11821949; doi:10.3389/fvets.2025.1494892)

## Slide 1
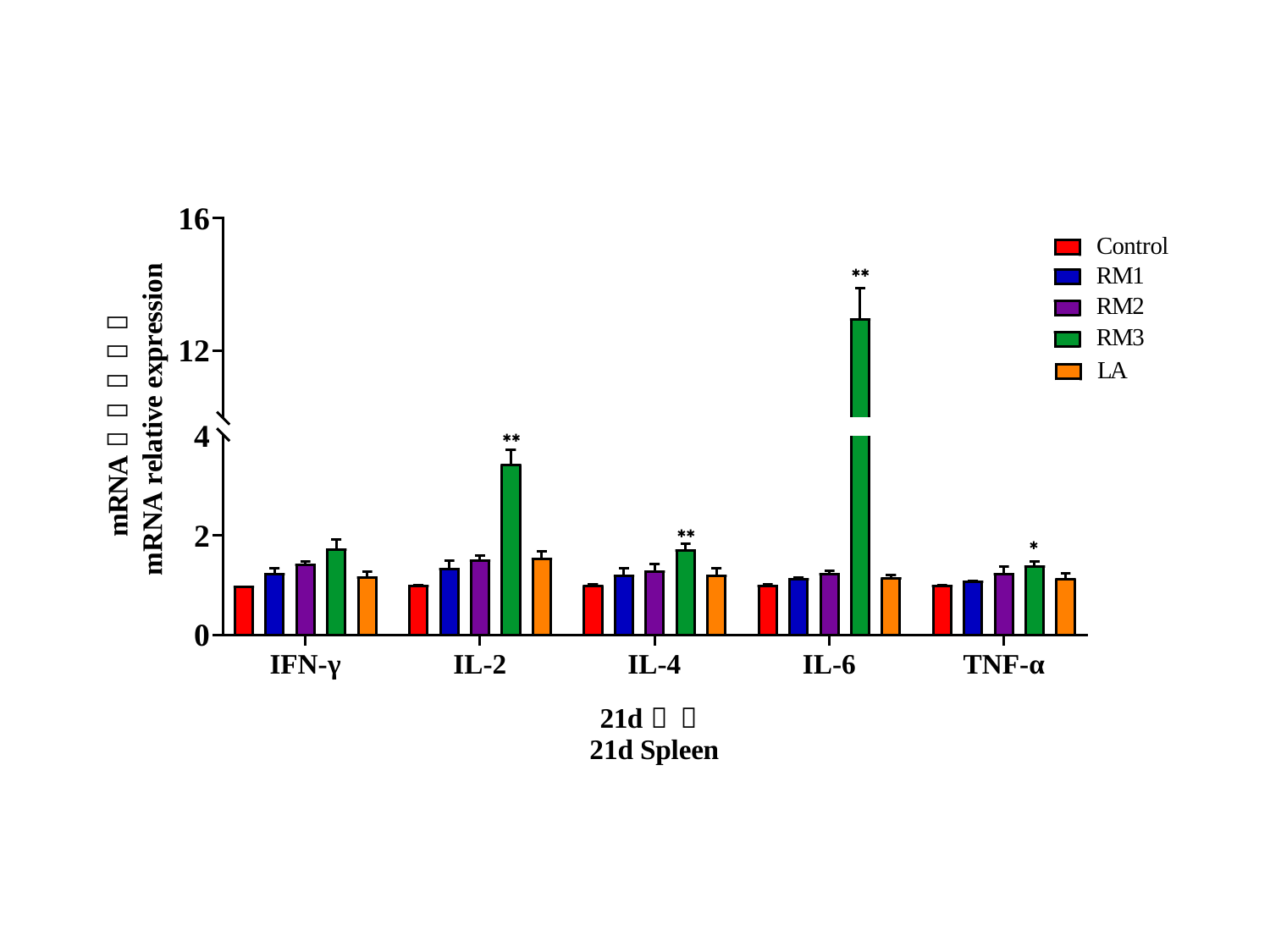

## Slide 2
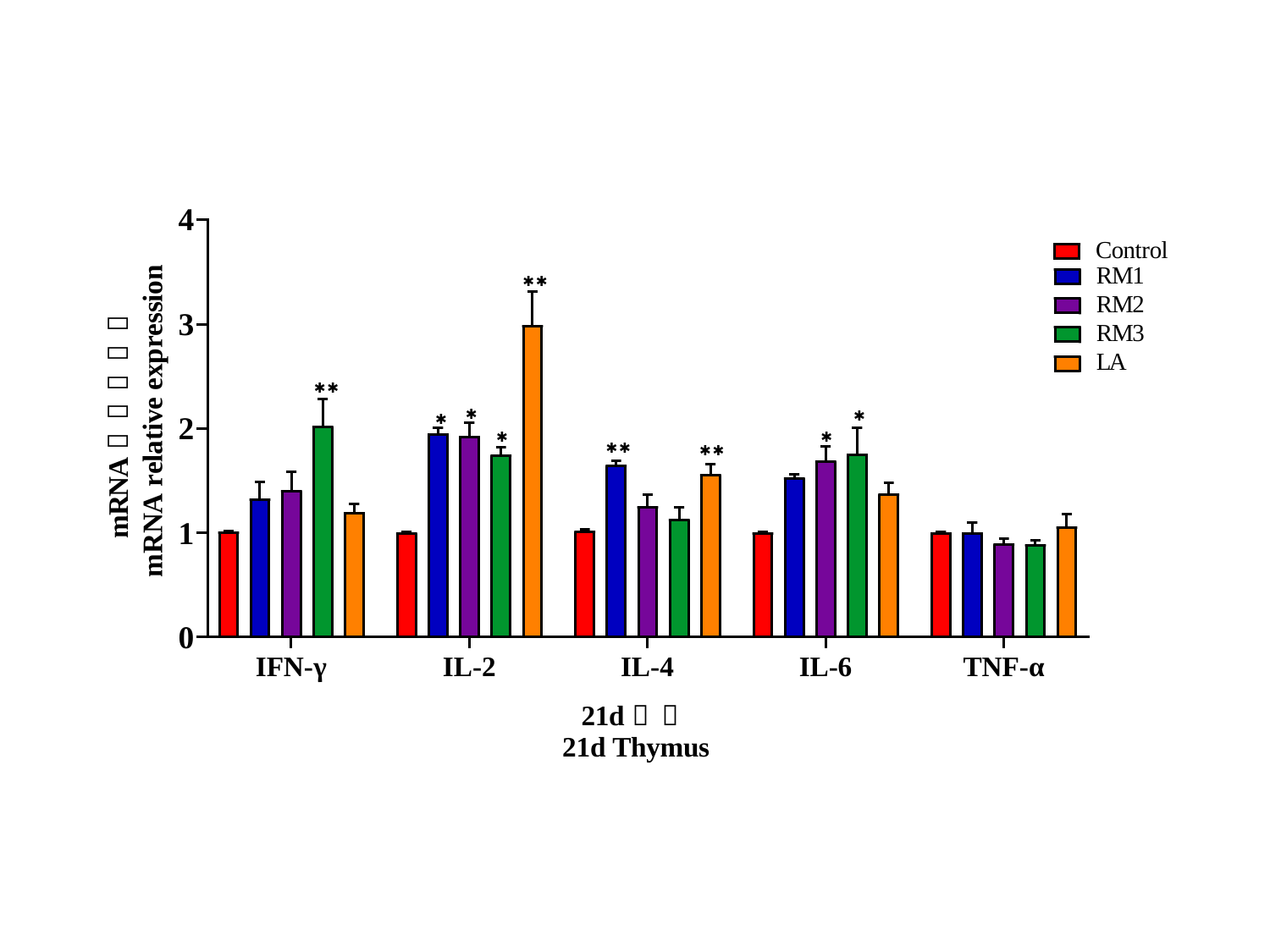

## Slide 3
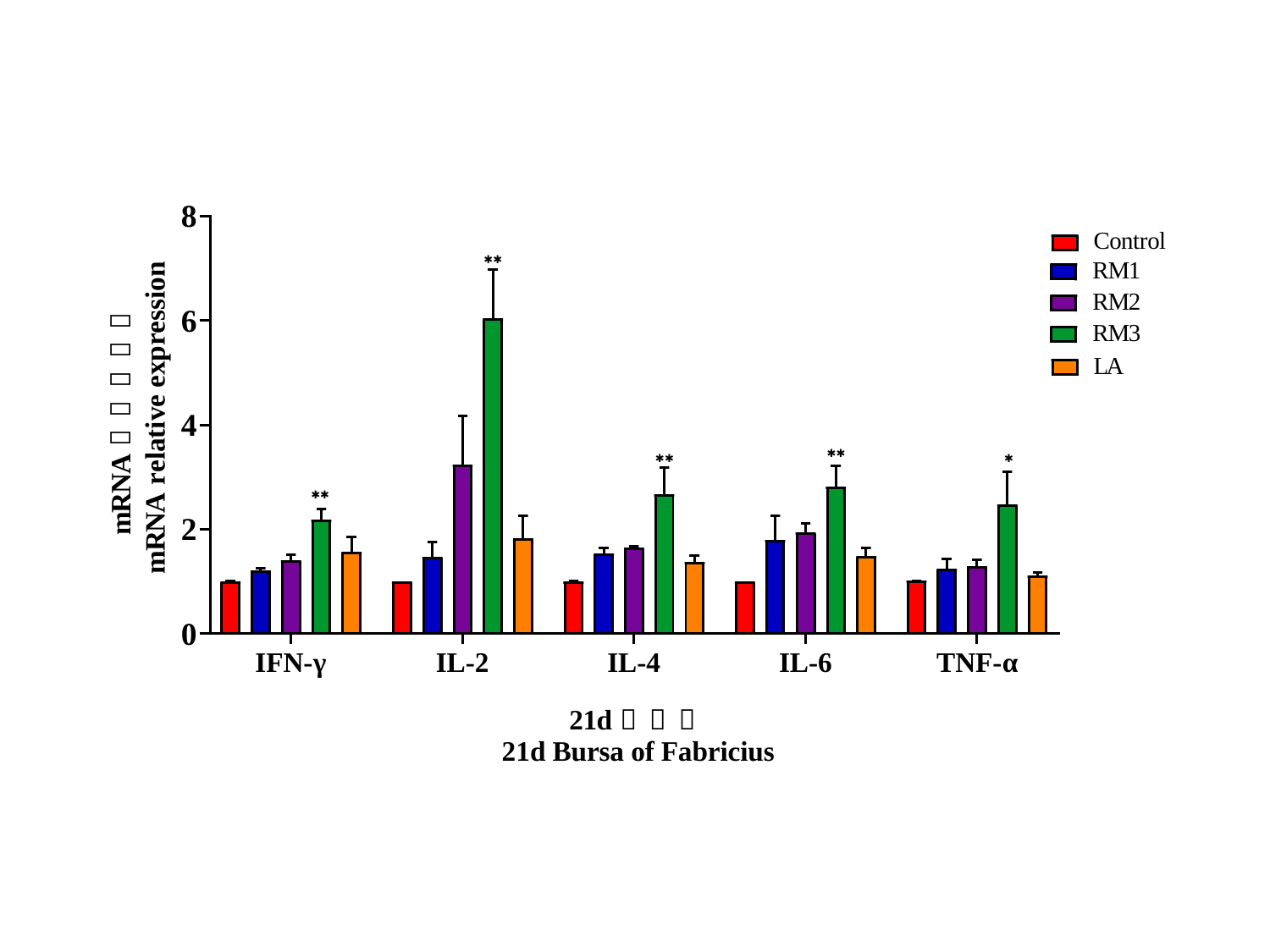

Supplement: Supplementary file 1 [file Data_Sheet_1.ZIP › the Article raw data/mRNA expression levels of antioxidant and immune factors in immune organs by qpcr/21d-免疫因子基因qpcr/21d免疫基因qpcr(带数据源).pptx]

## Slide 1
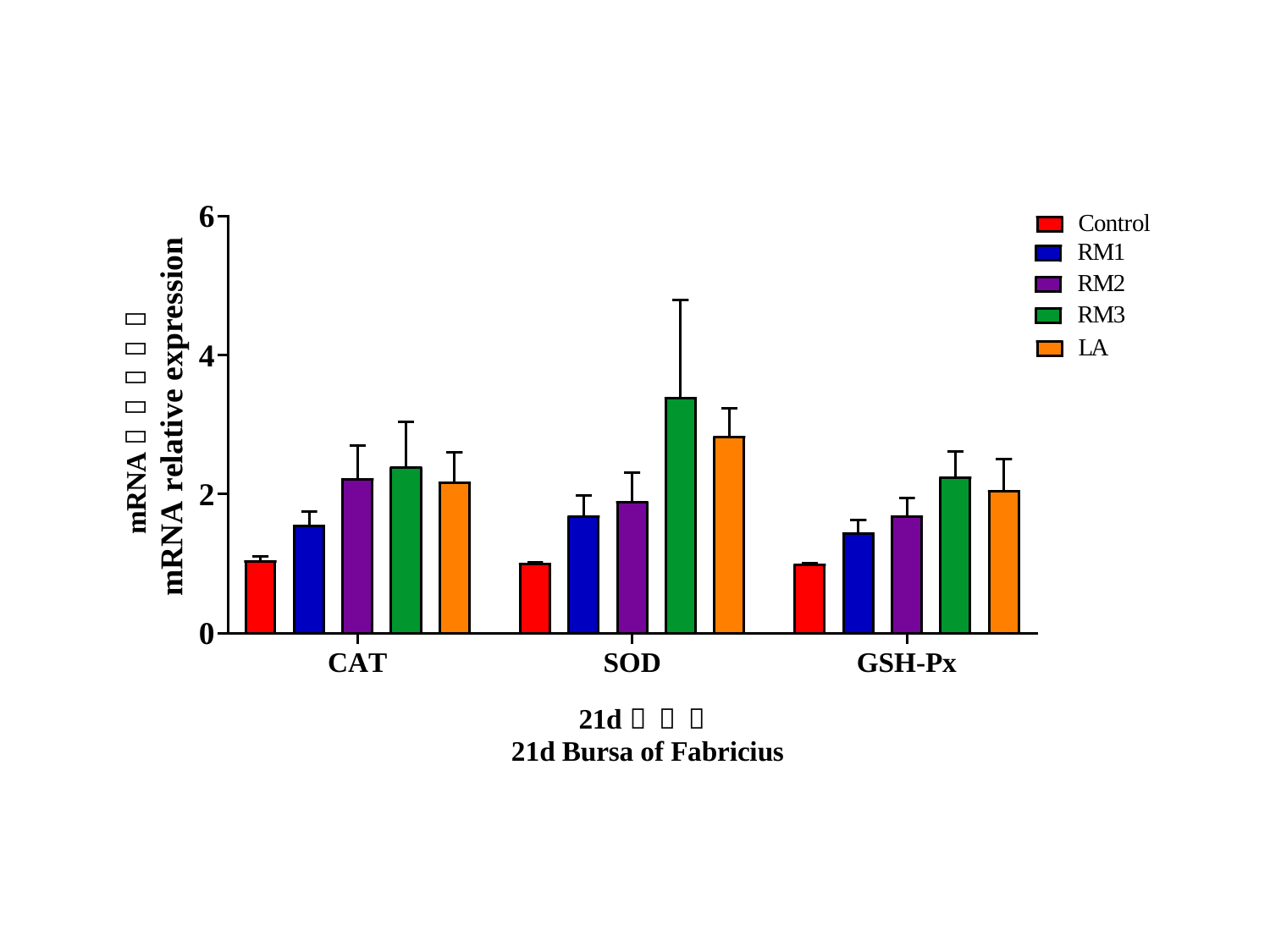

## Slide 2
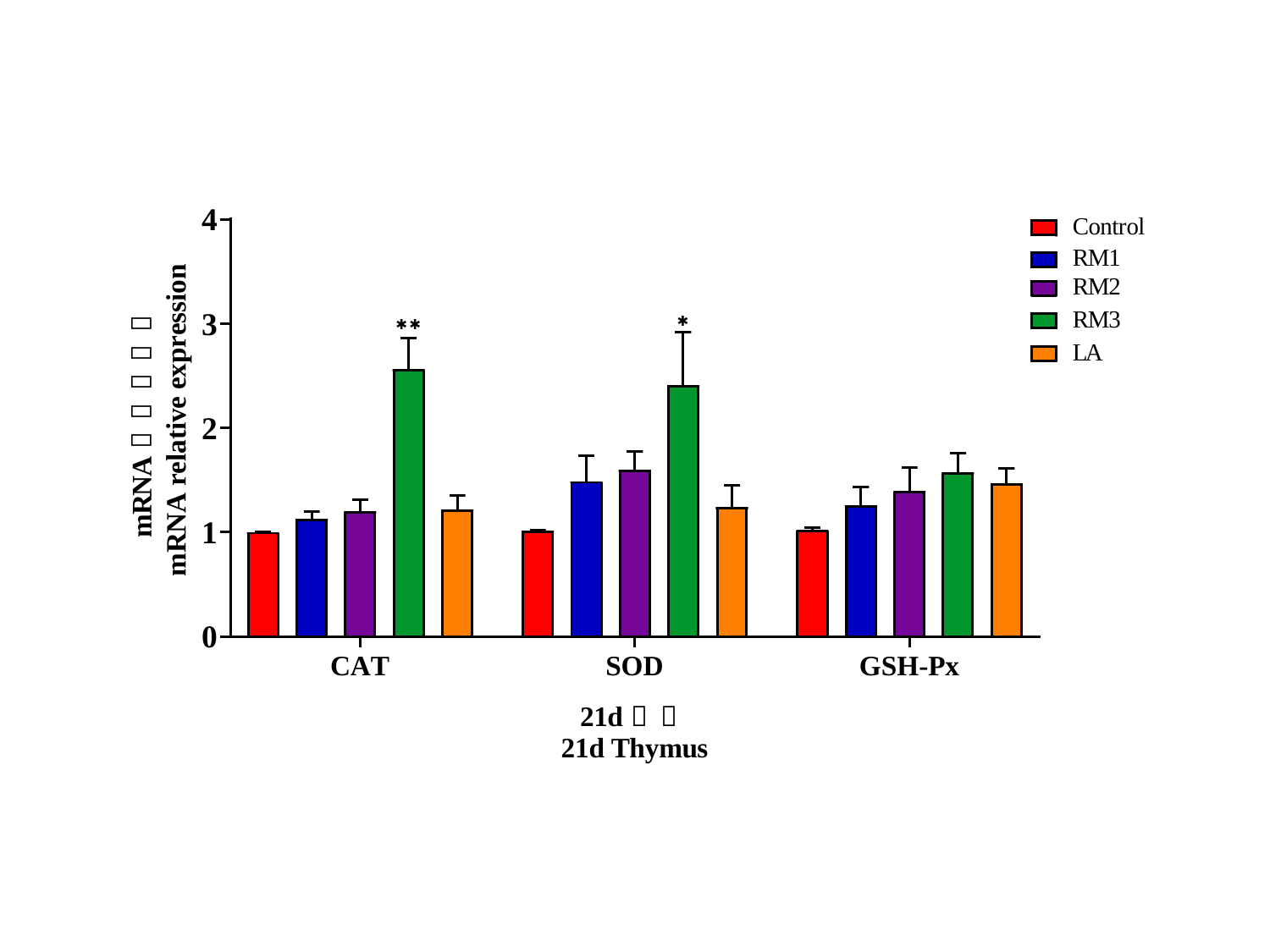

## Slide 3
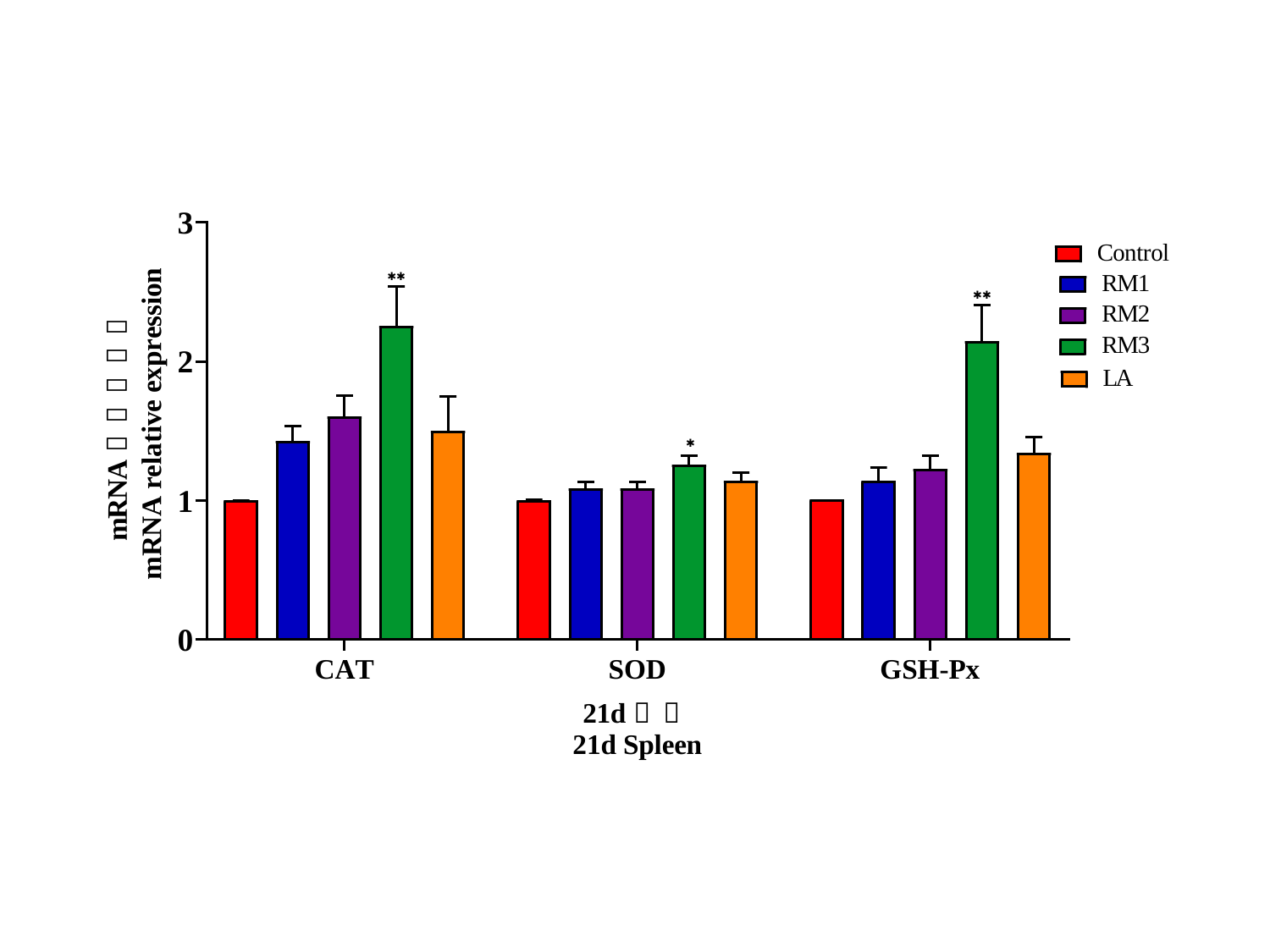

Supplement: Supplementary file 1 [file Data_Sheet_1.ZIP › the Article raw data/mRNA expression levels of antioxidant and immune factors in immune organs by qpcr/21d-抗氧化基因qpcr/21d抗氧化基因qpcr(带数据源).pptx]

## Slide 1
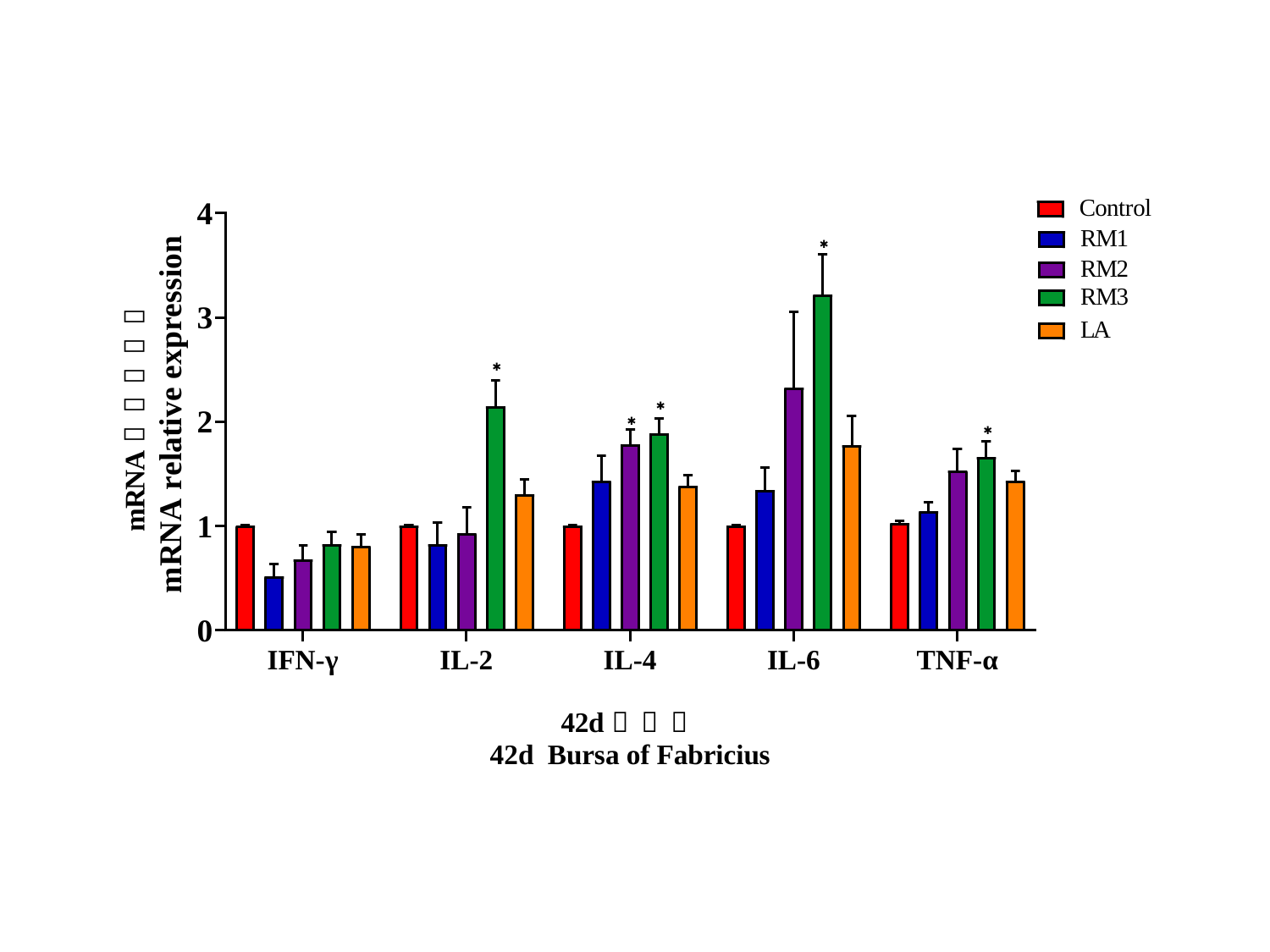

## Slide 2
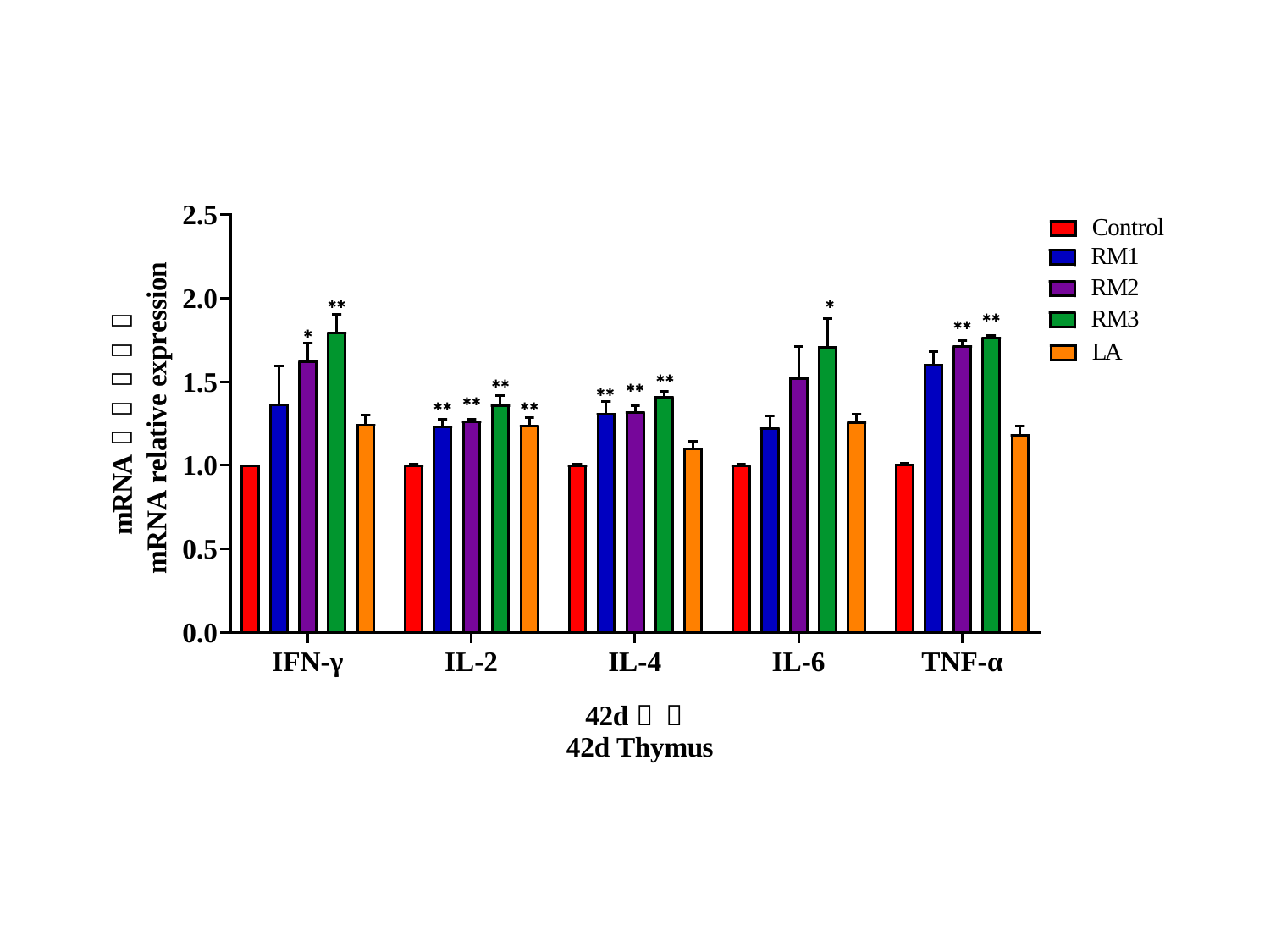

## Slide 3
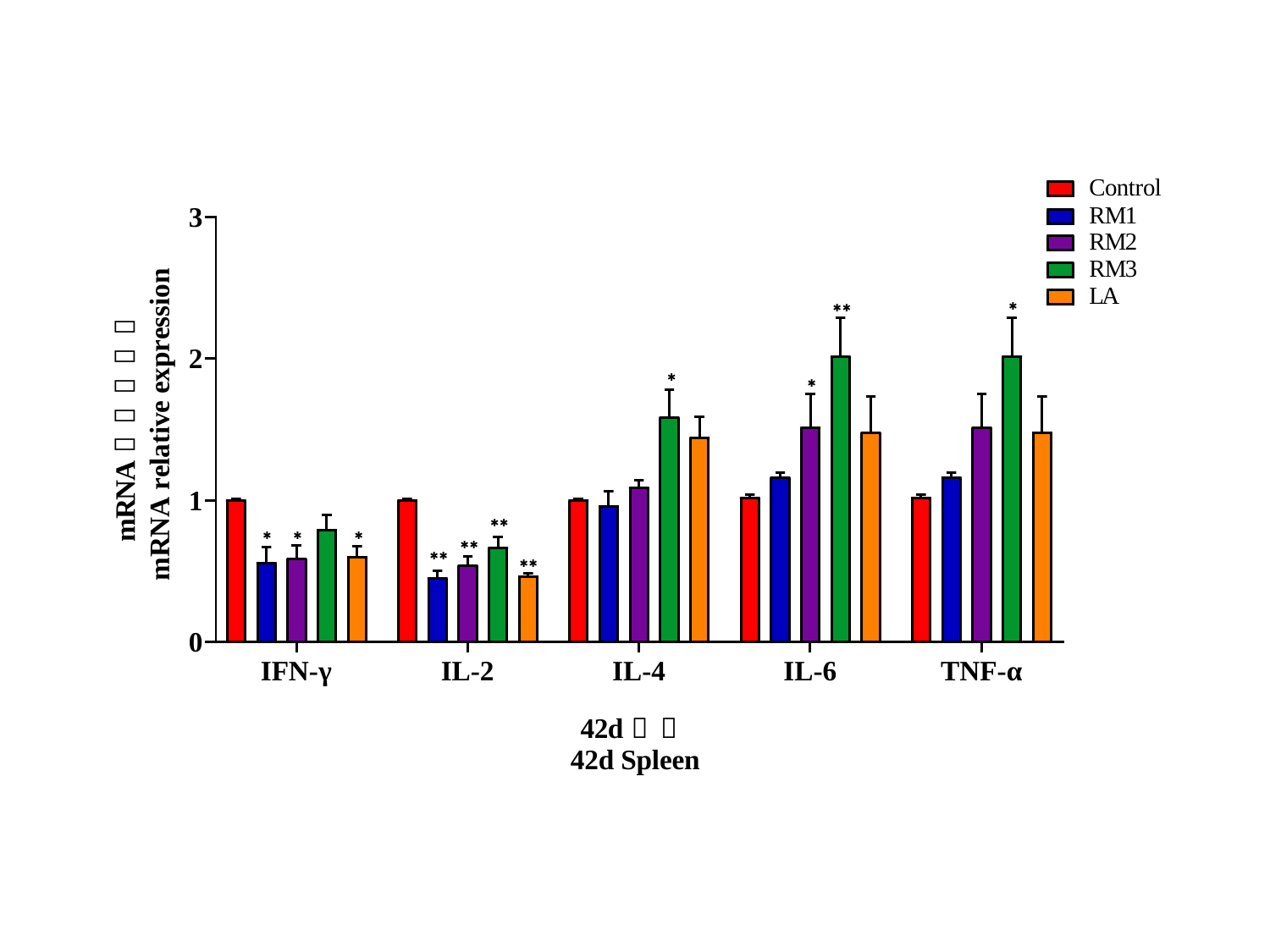

Supplement: Supplementary file 1 [file Data_Sheet_1.ZIP › the Article raw data/mRNA expression levels of antioxidant and immune factors in immune organs by qpcr/42d-免疫因子基因qpcr/42d免疫基因qpcr(带数据源).pptx]

## Slide 1
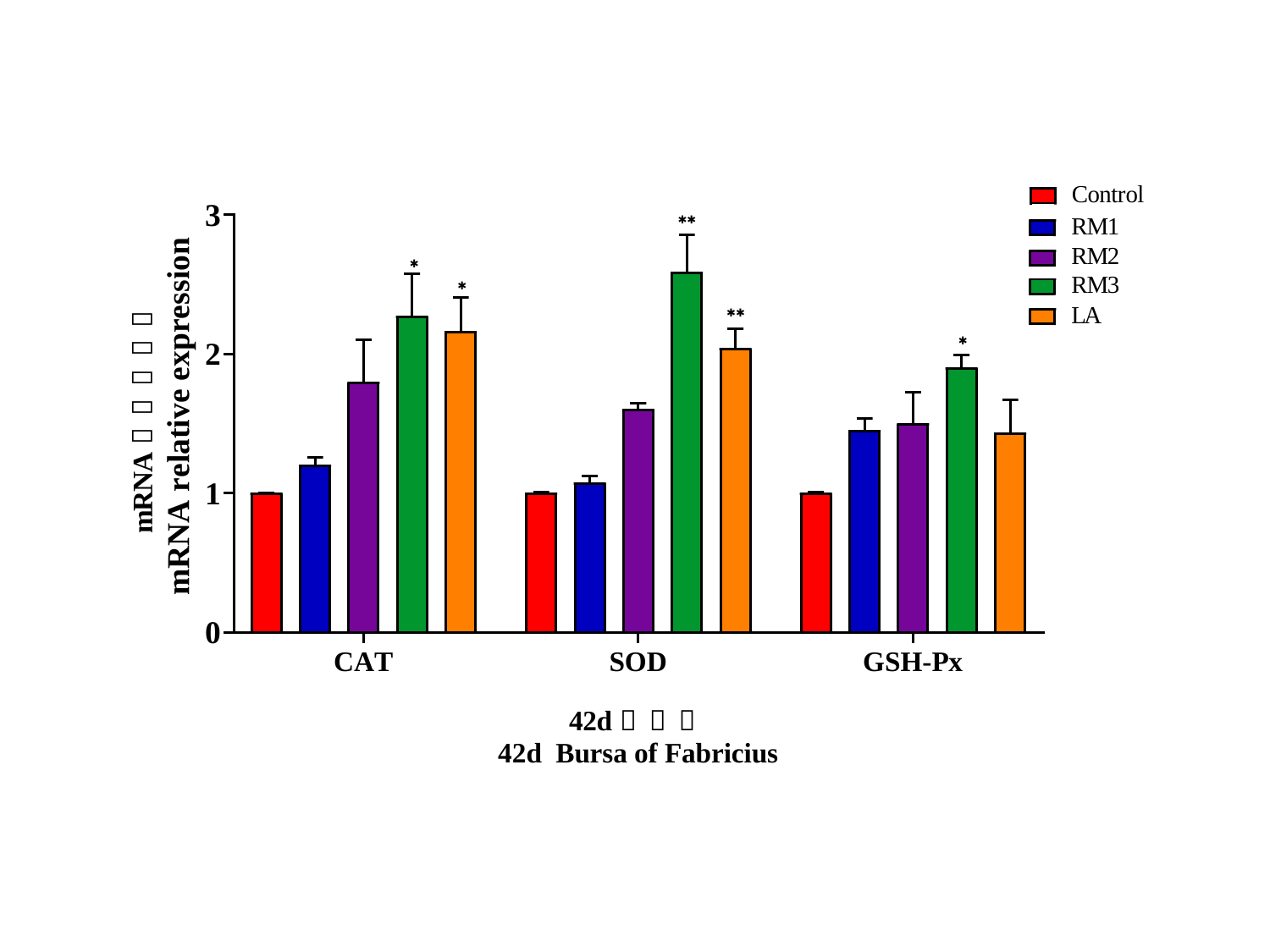

## Slide 2
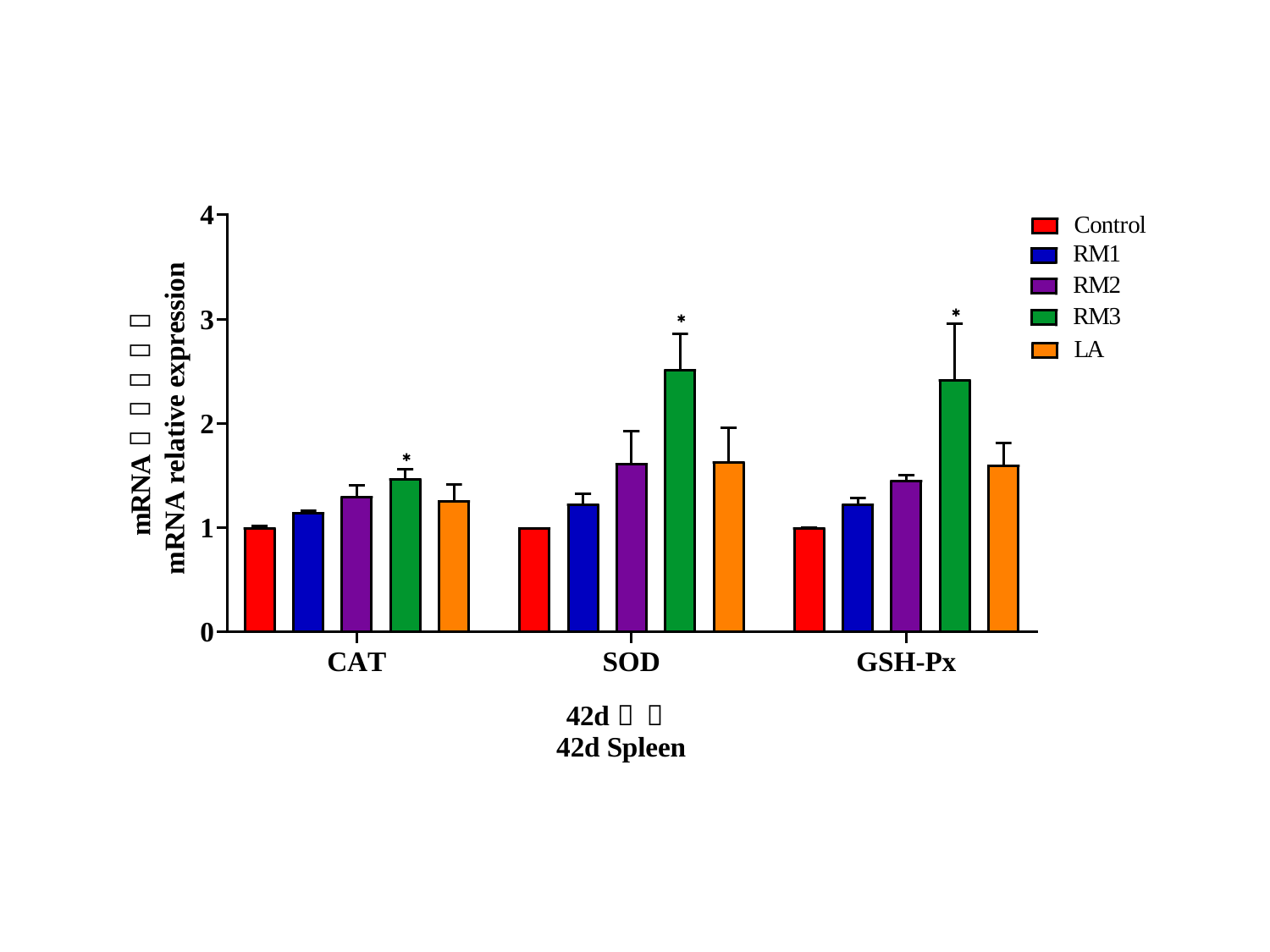

## Slide 3
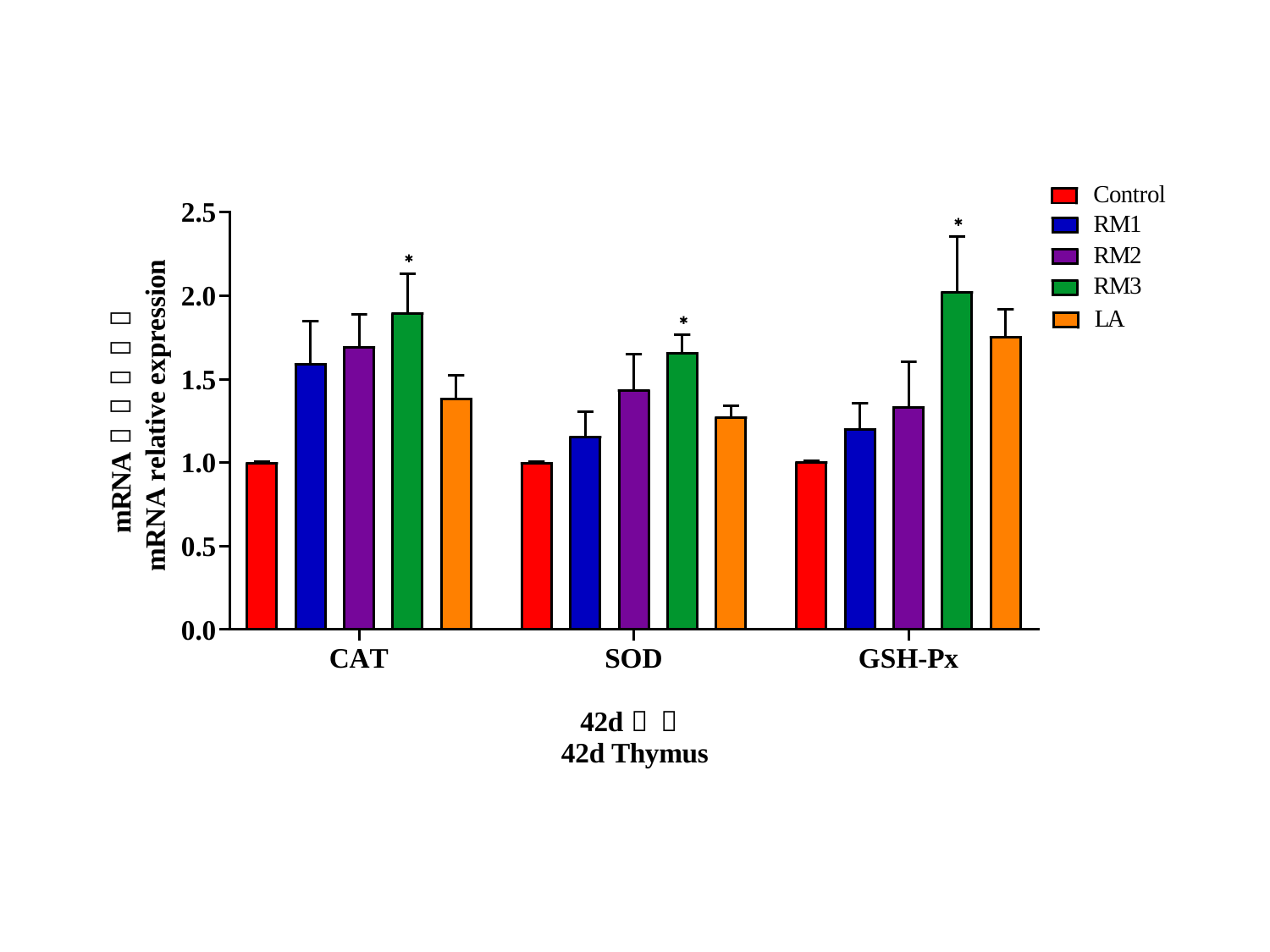

Supplement: Supplementary file 1 [file Data_Sheet_1.ZIP › the Article raw data/mRNA expression levels of antioxidant and immune factors in immune organs by qpcr/42d-抗氧化基因qpcr/42d抗氧化基因qpcr(带数据源).pptx]

## Slide 1
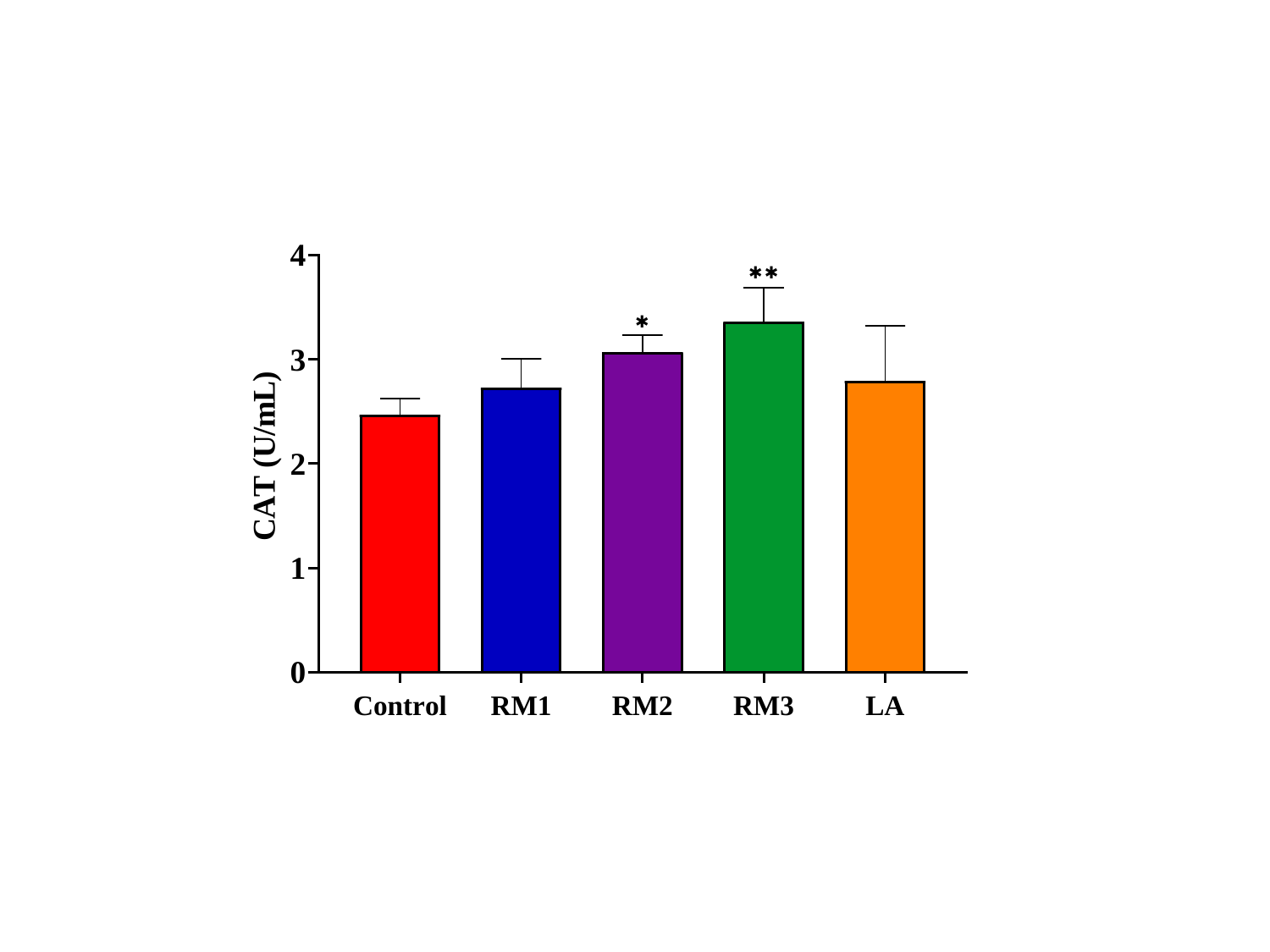

## Slide 2
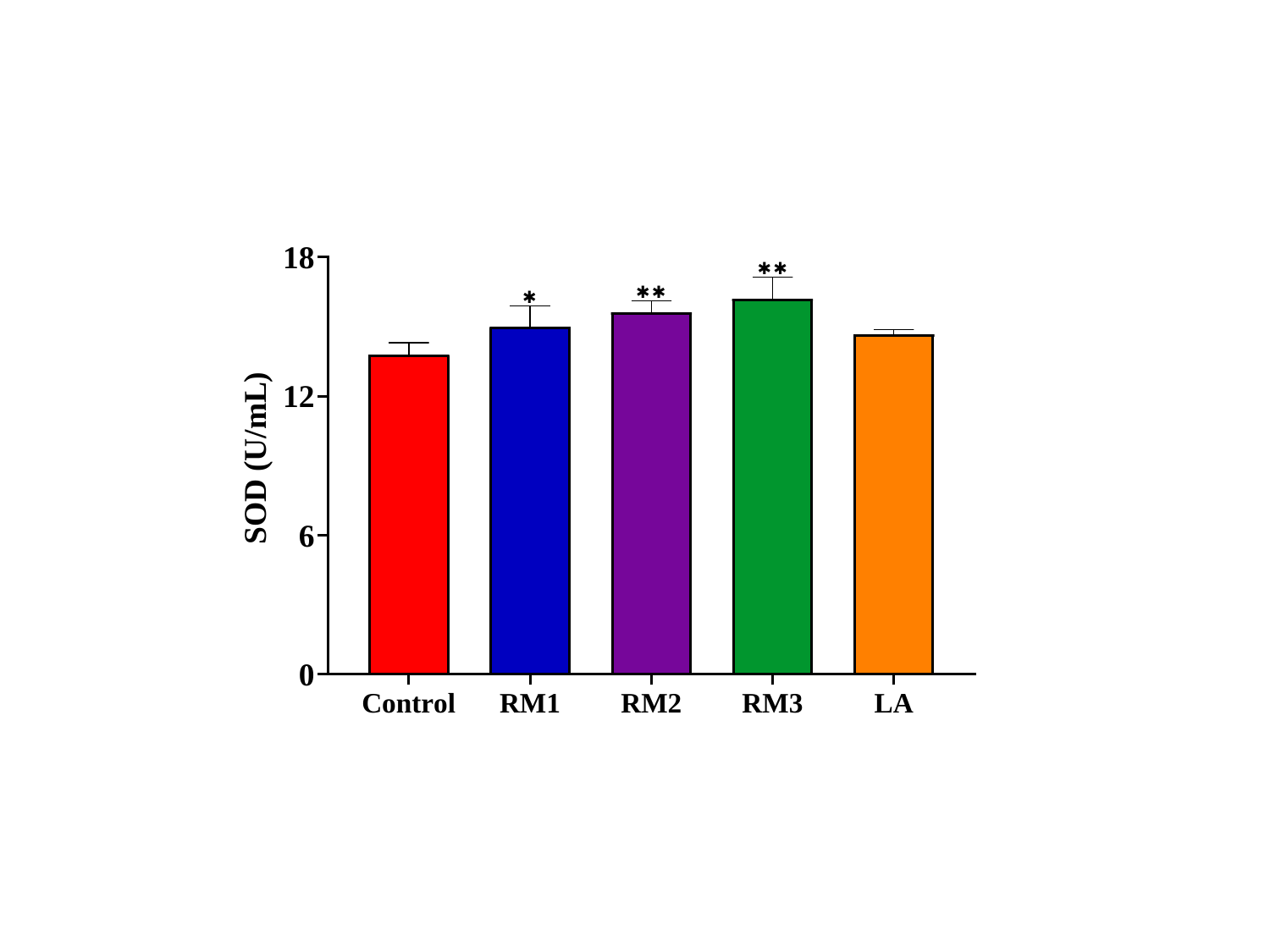

## Slide 3
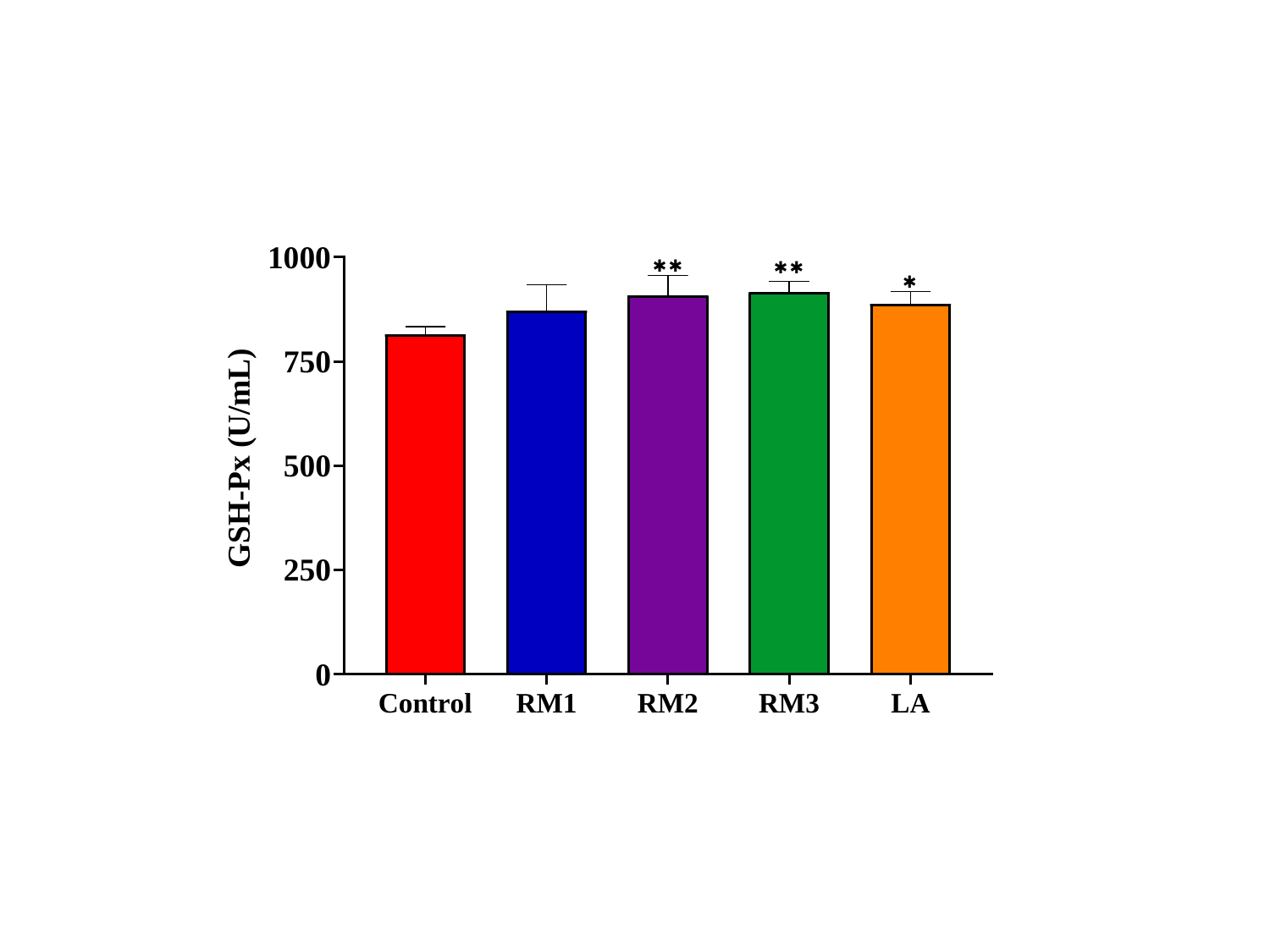

## Slide 4
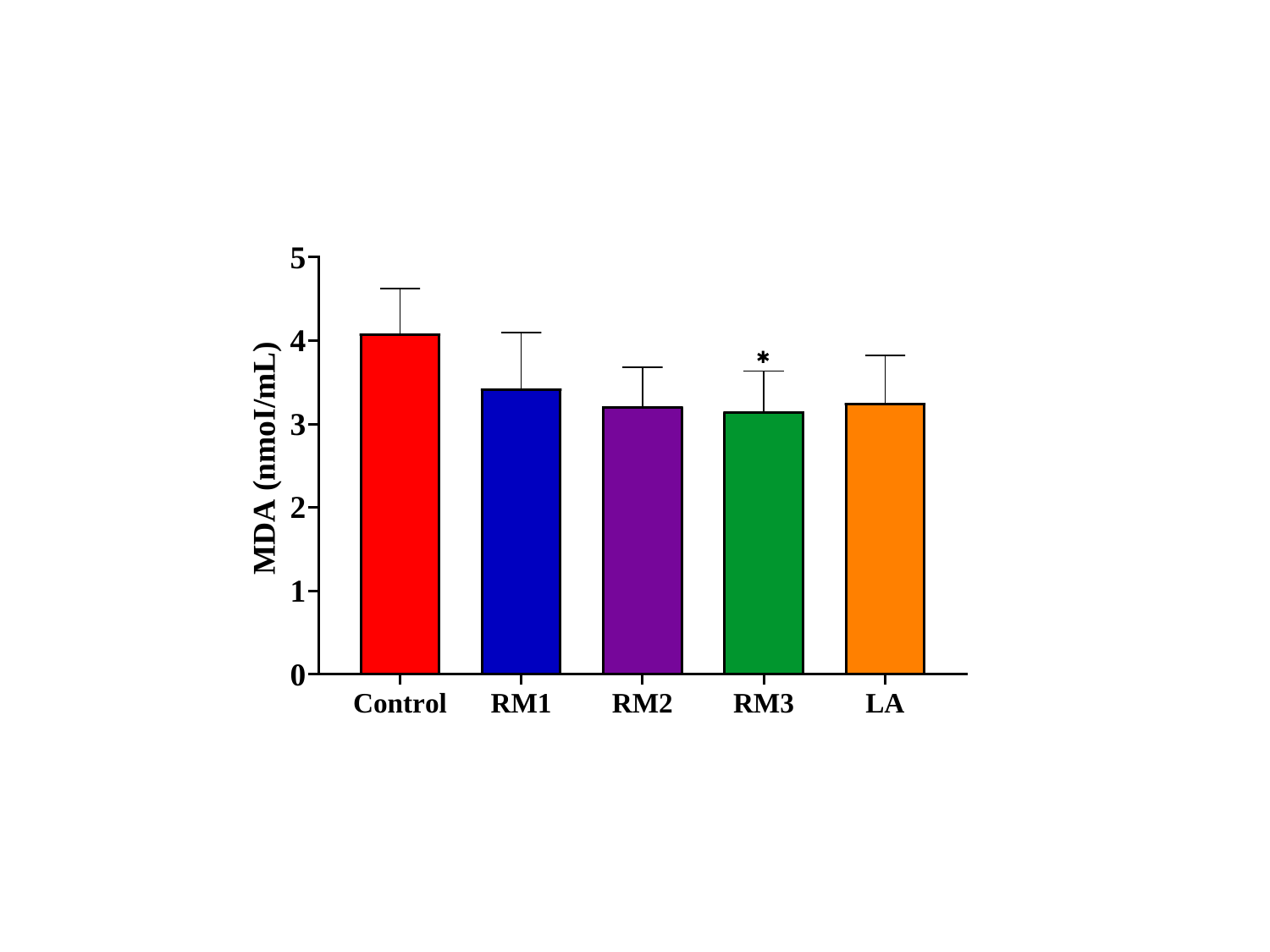

Supplement: Supplementary file 1 [file Data_Sheet_1.ZIP › the Article raw data/Serum antioxidant index by elisa/21d试剂盒/21d抗氧化(带数据源).pptx]

## Slide 1
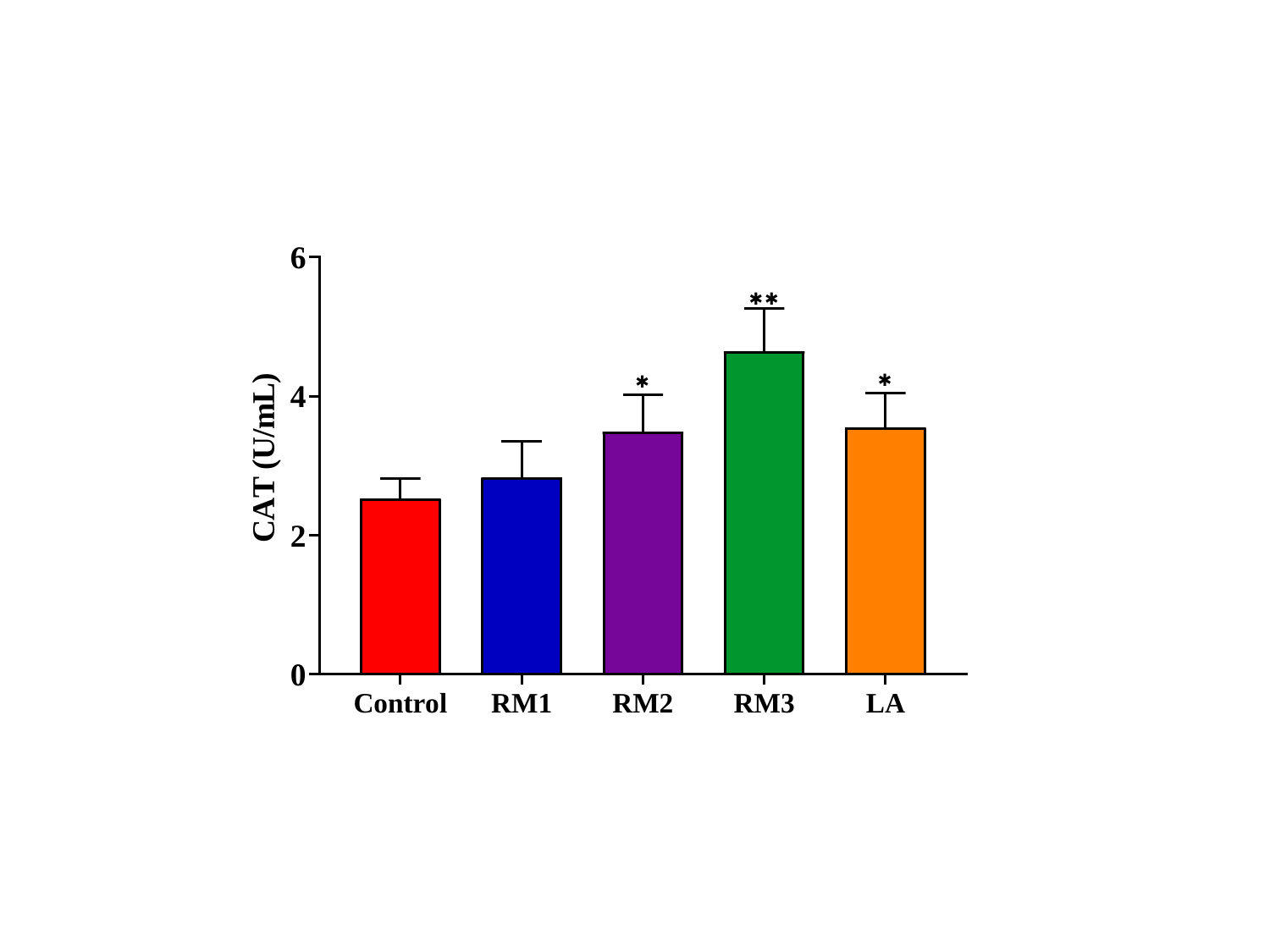

## Slide 2
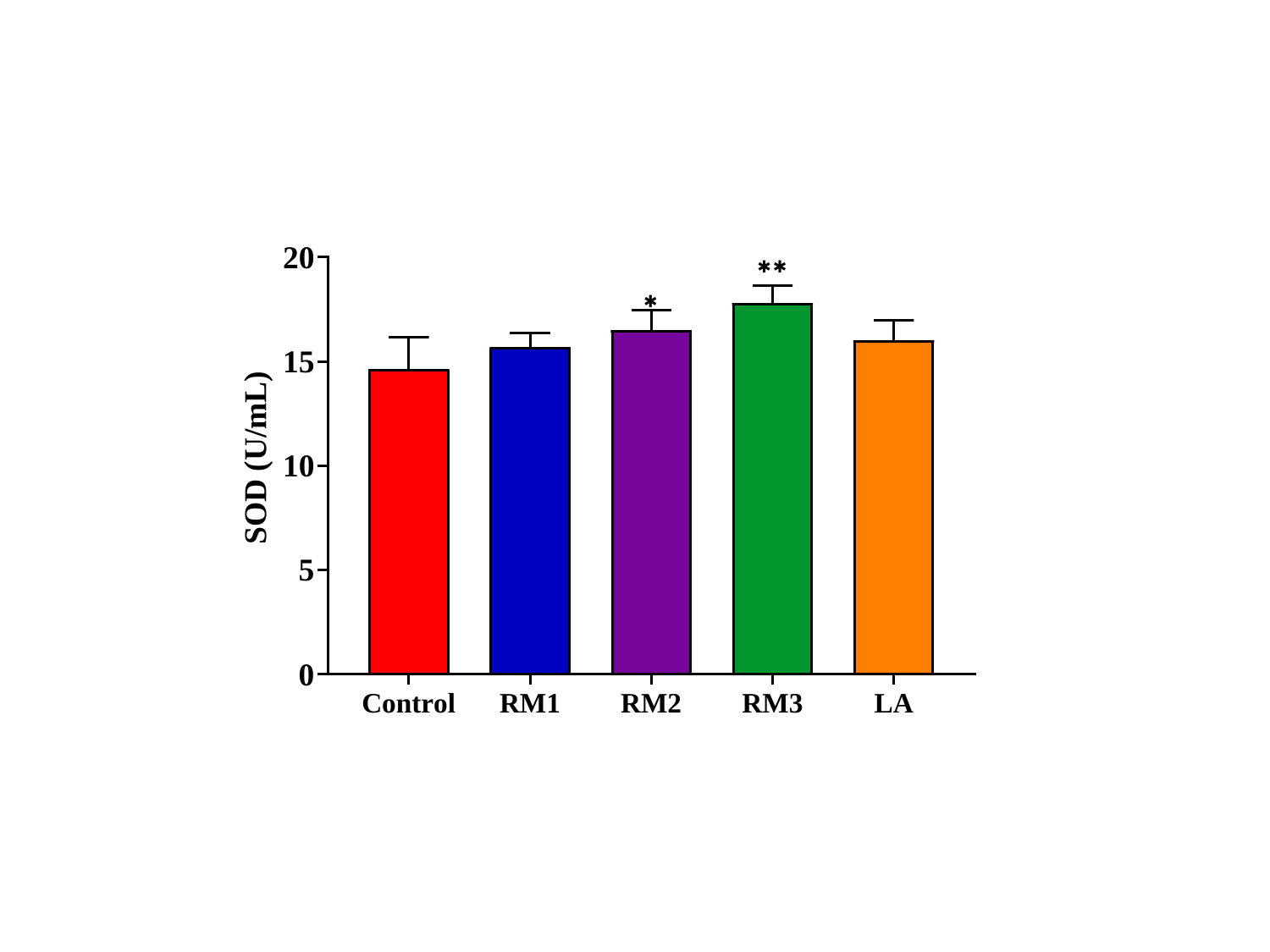

## Slide 3
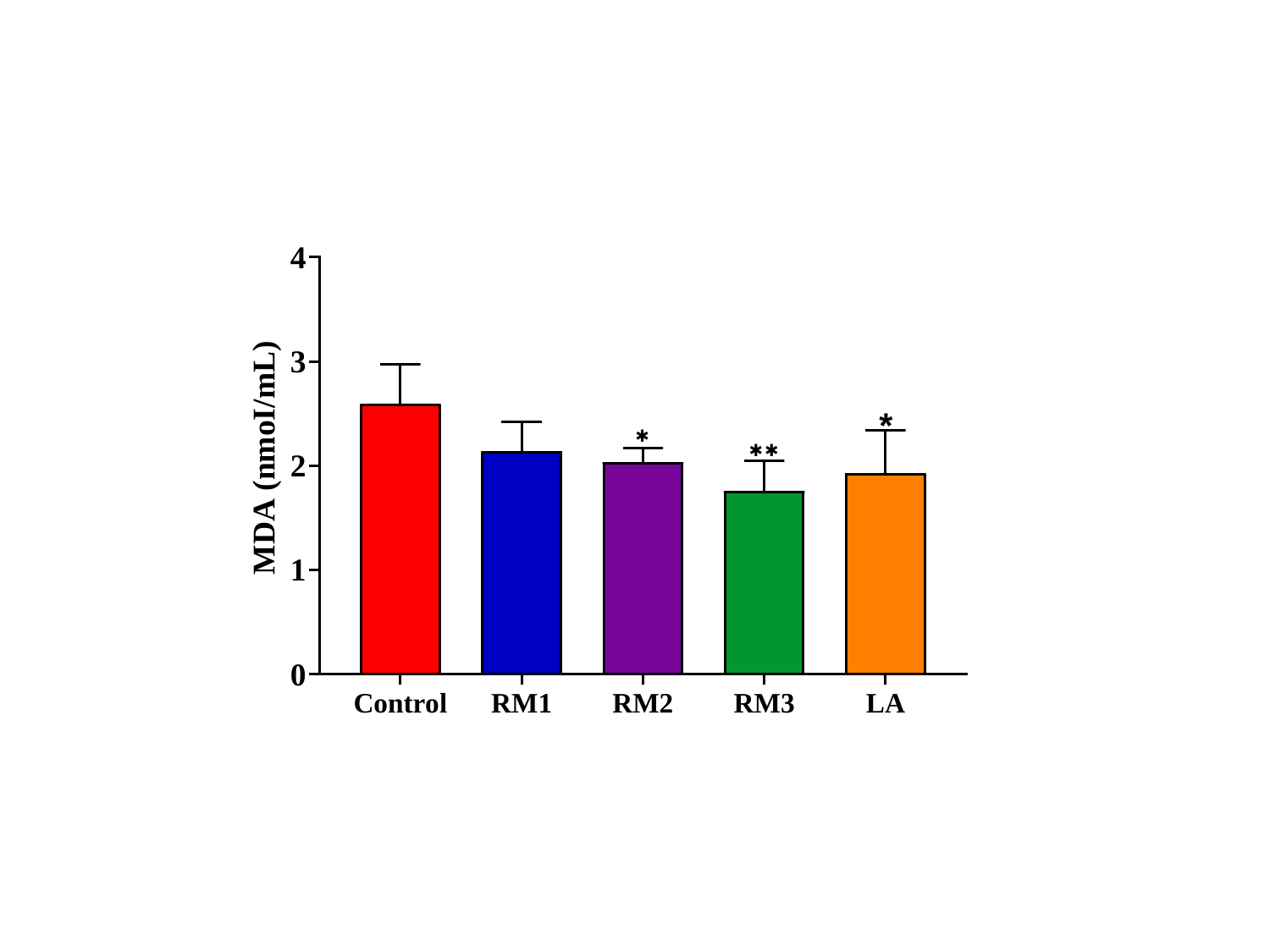

## Slide 4
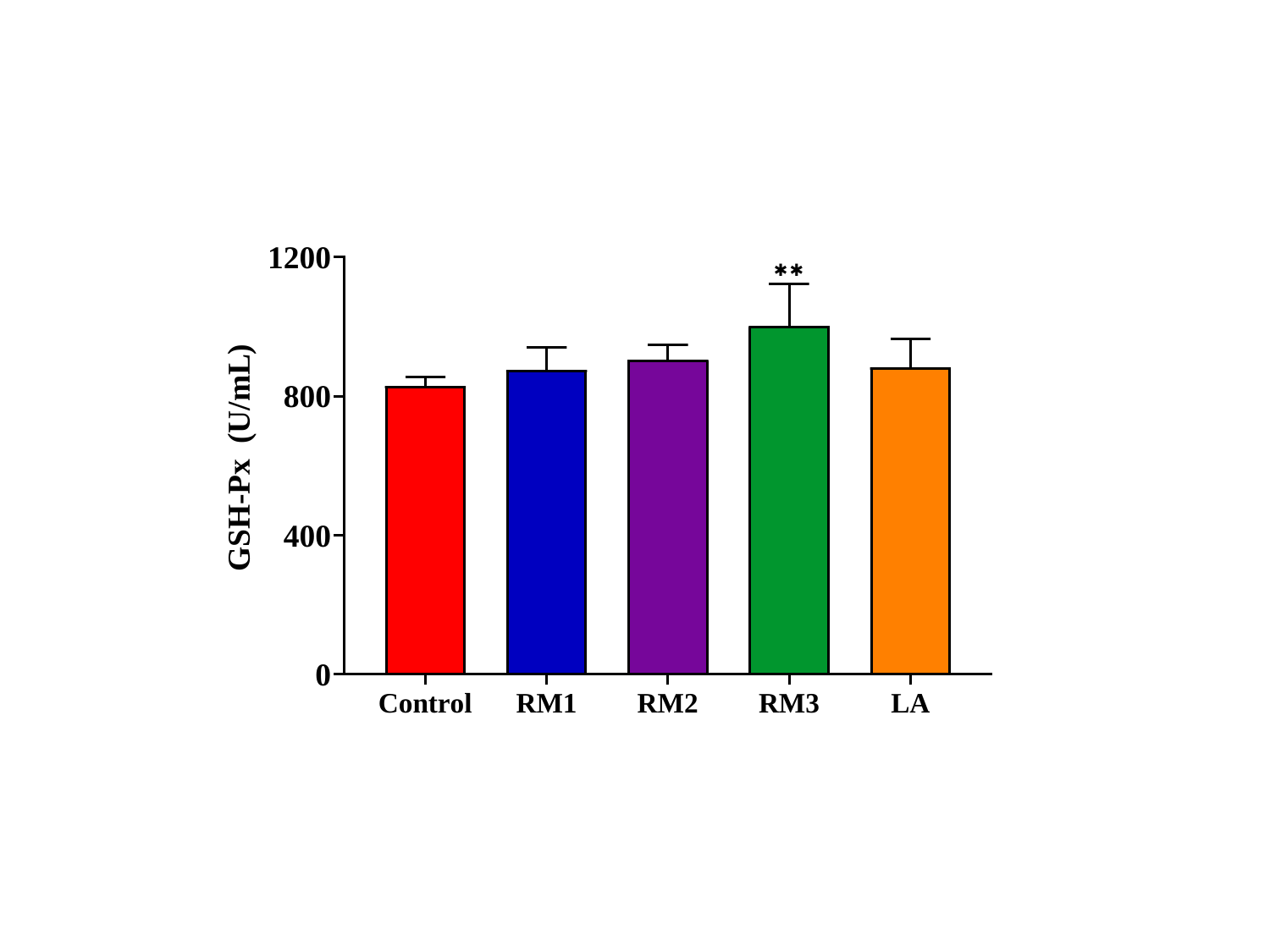

Supplement: Supplementary file 1 [file Data_Sheet_1.ZIP › the Article raw data/Serum antioxidant index by elisa/42d试剂盒/42d抗氧化(带数据源).pptx]

## Slide 1
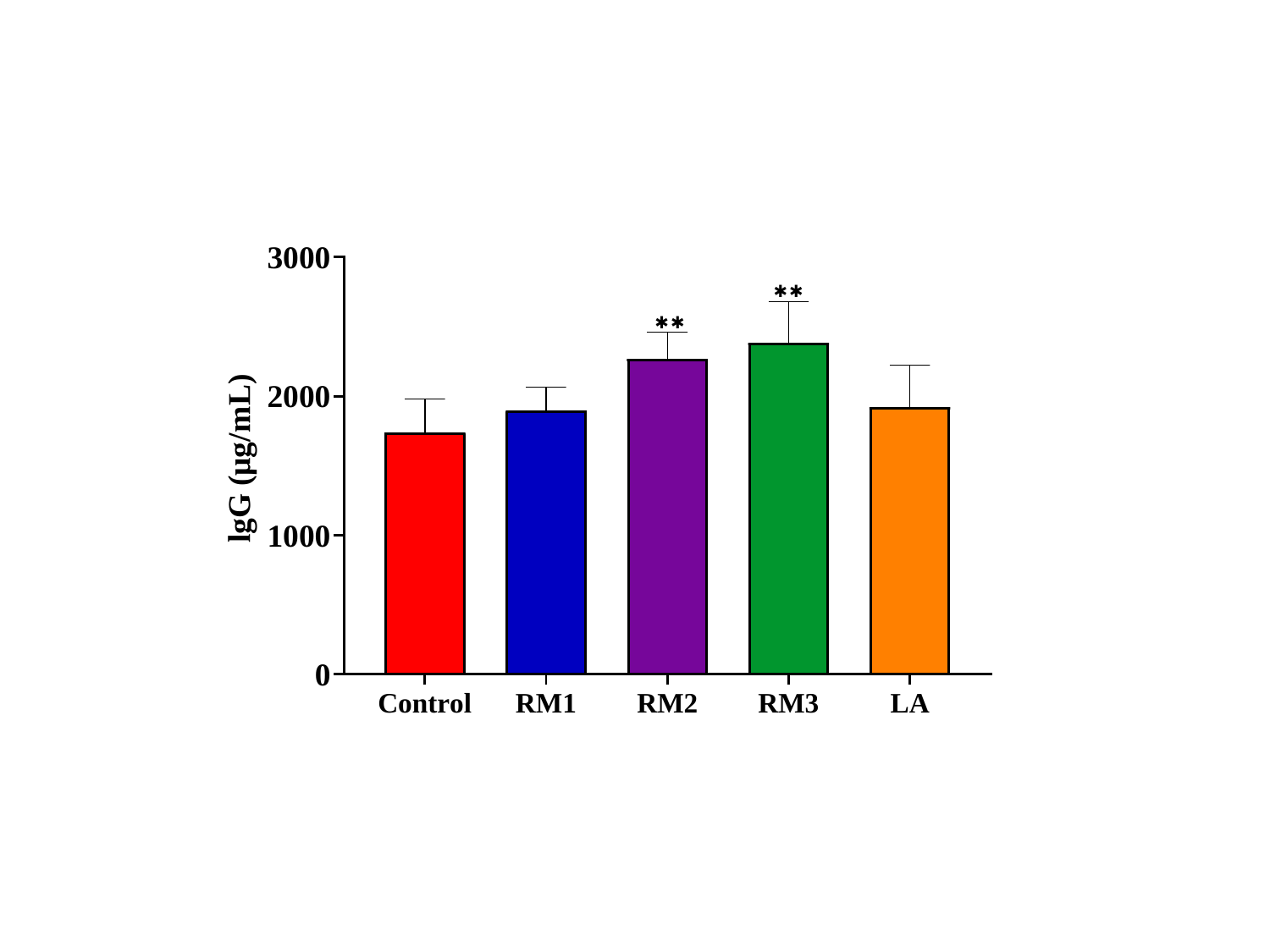

## Slide 2
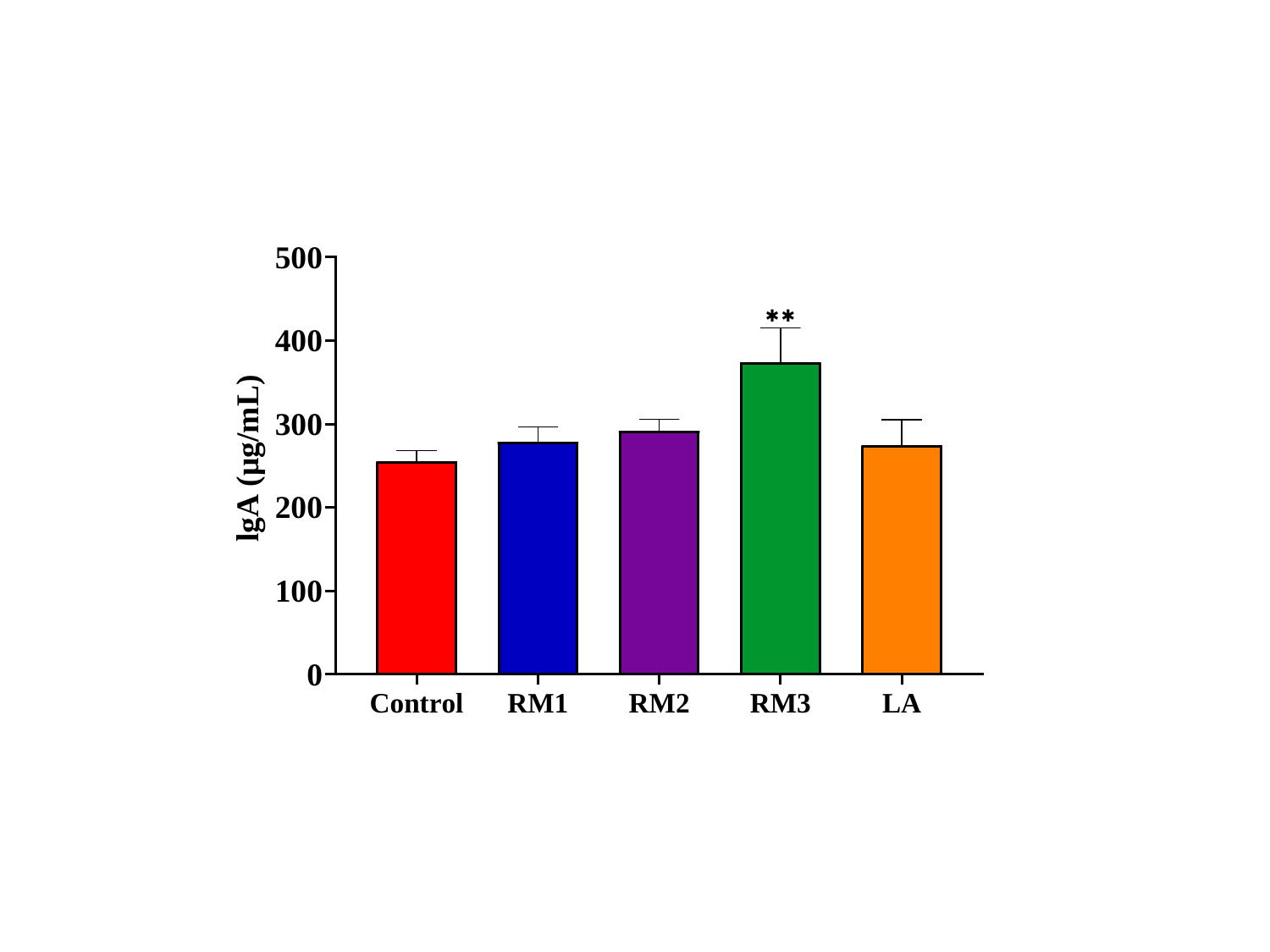

## Slide 3
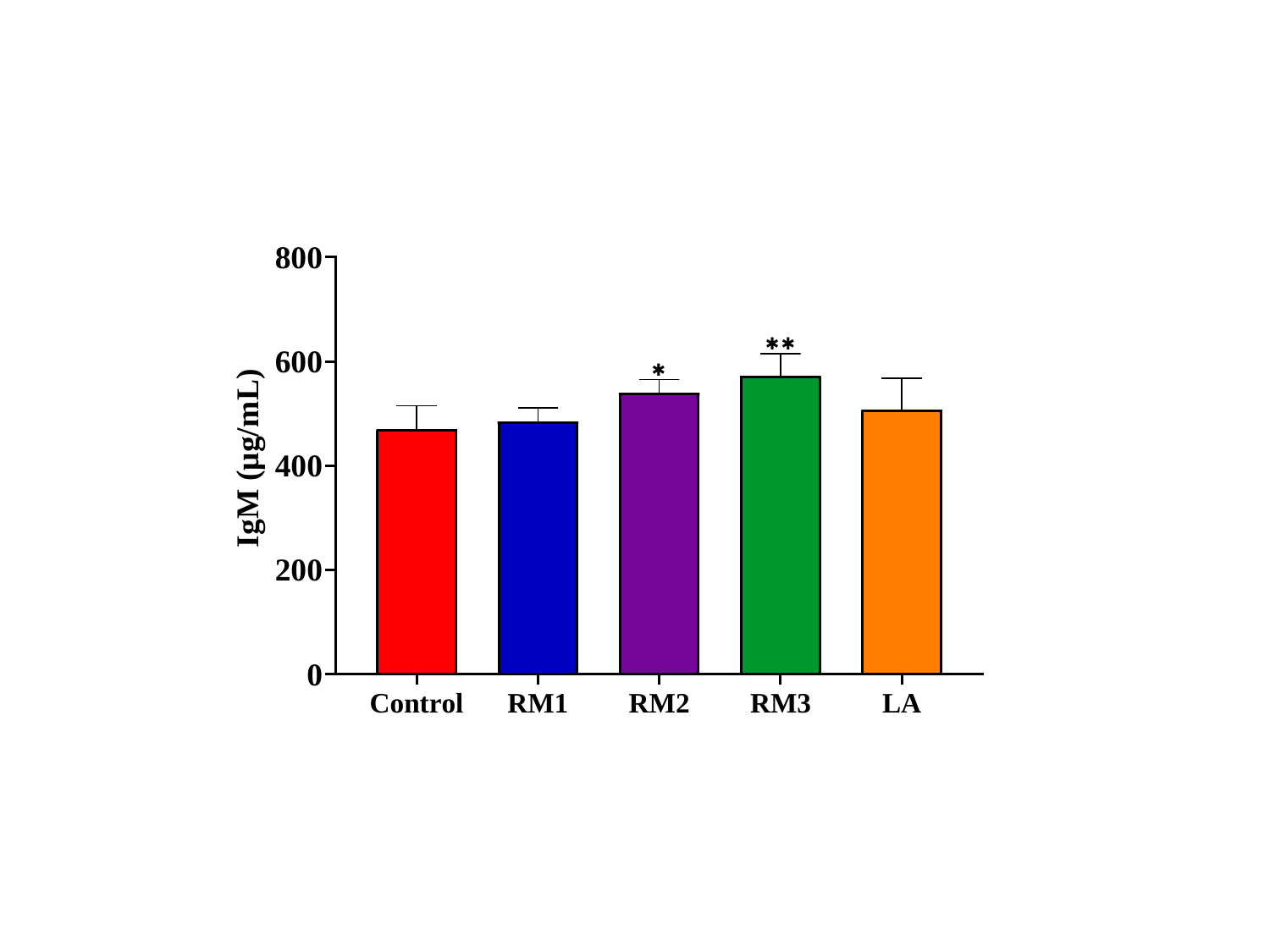

## Slide 4
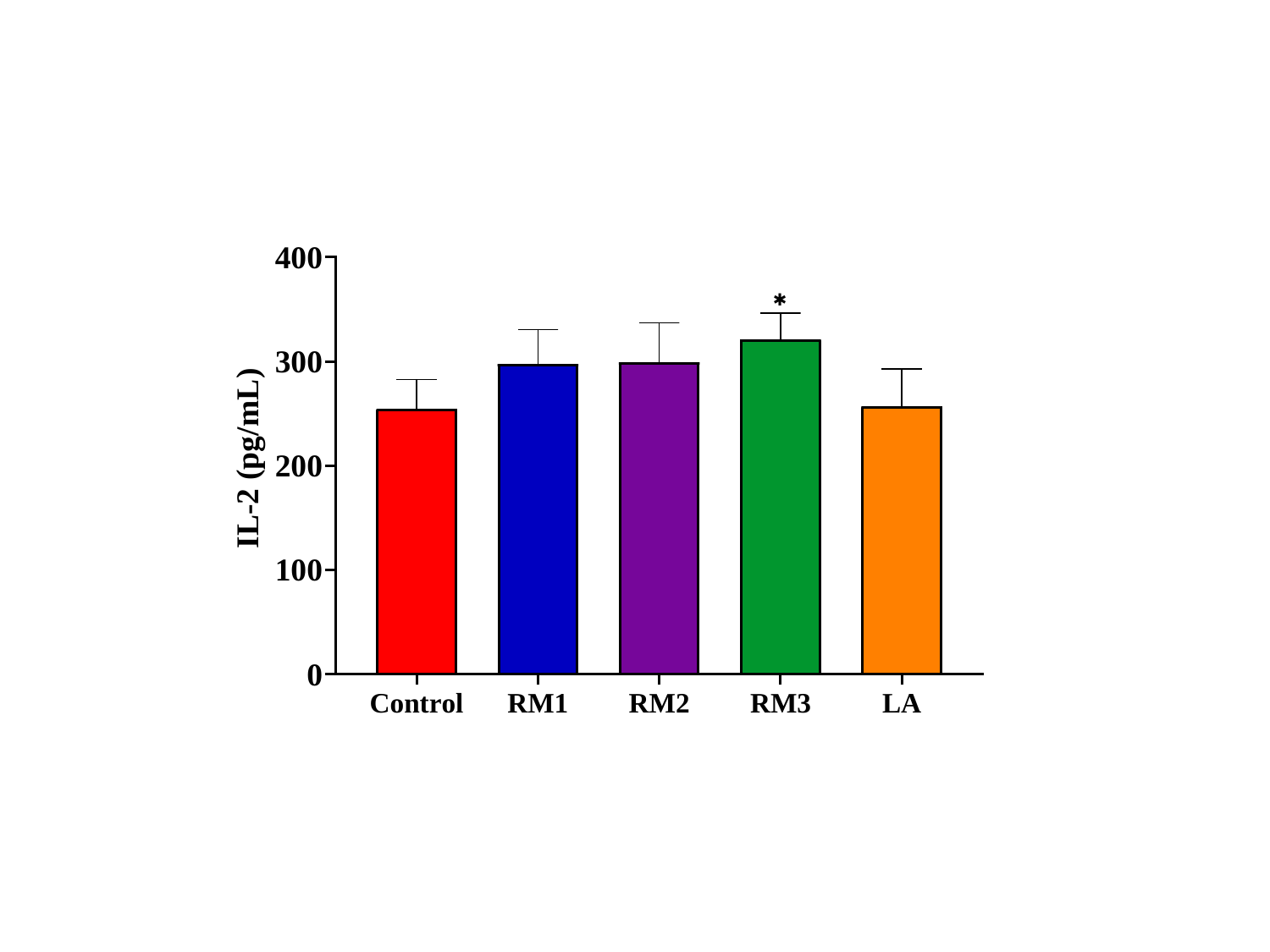

## Slide 5
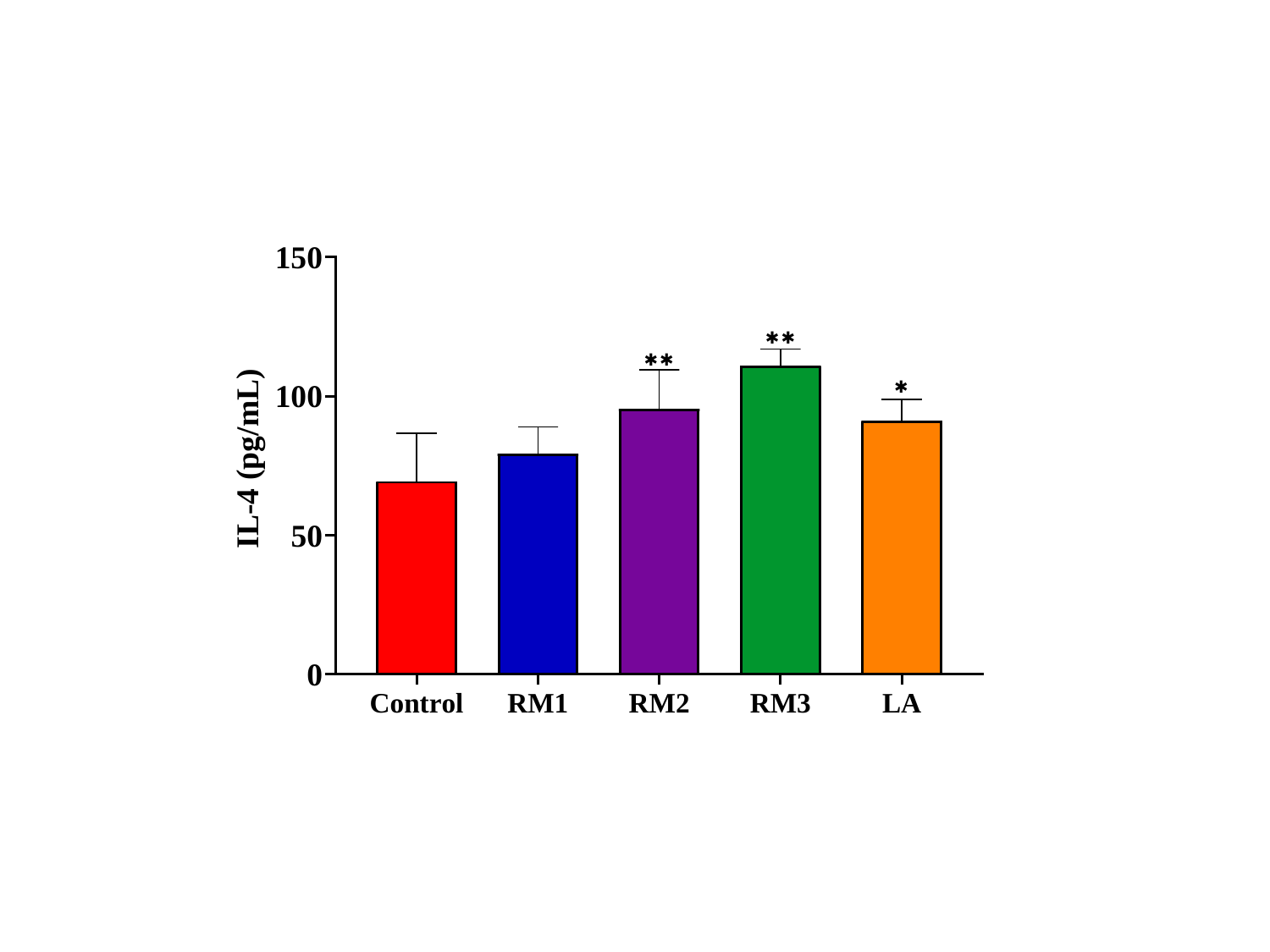

## Slide 6
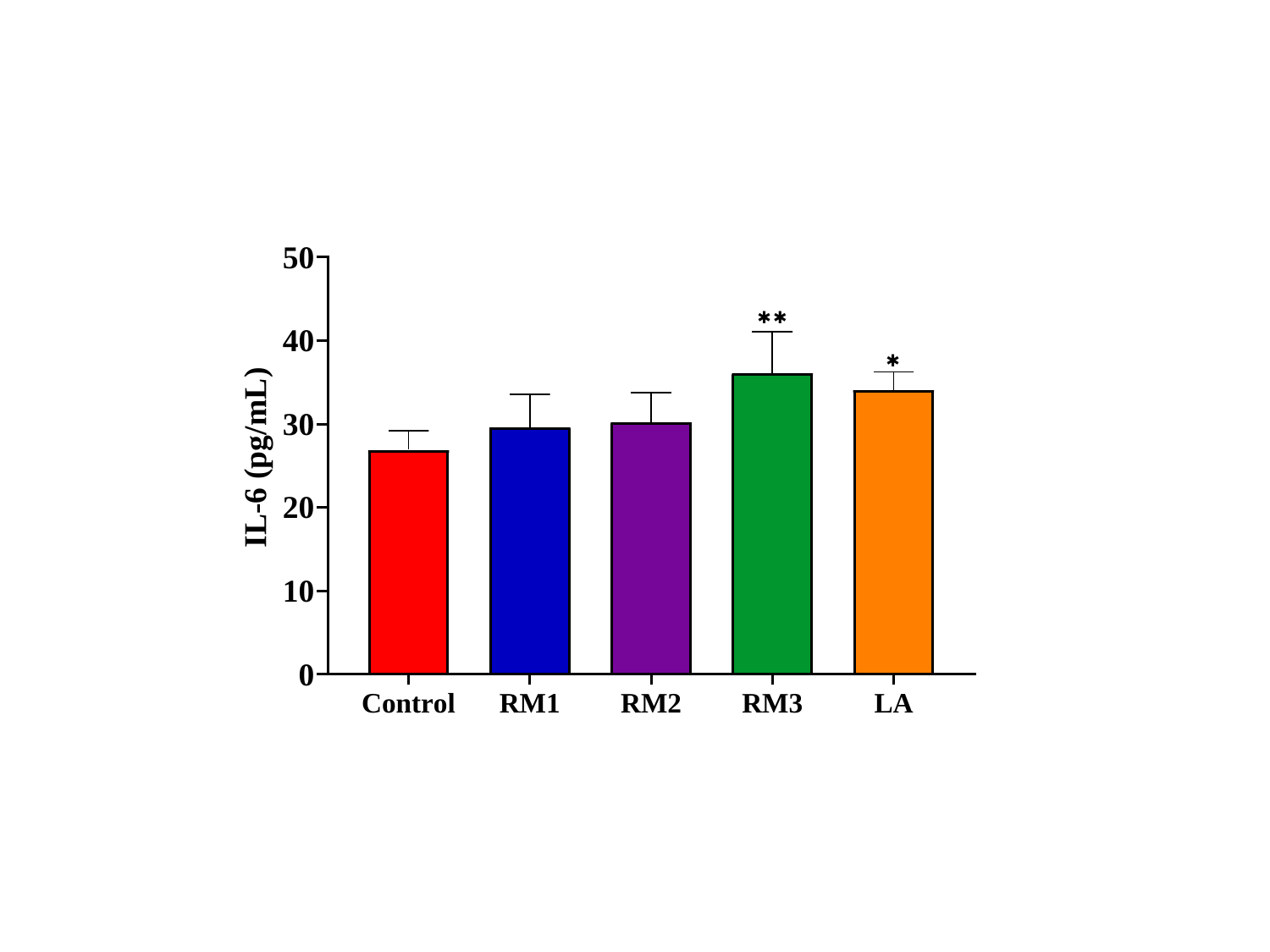

## Slide 7
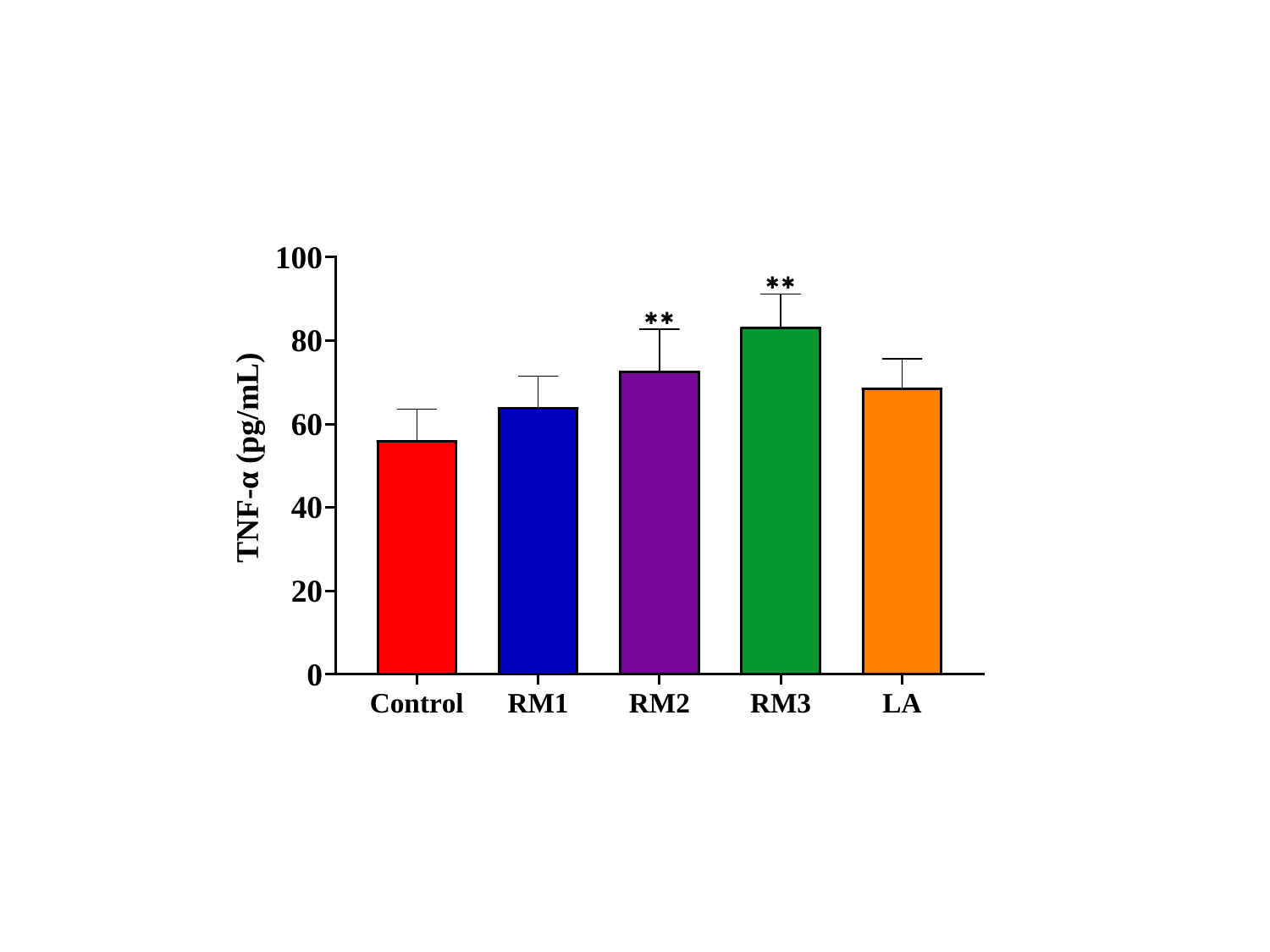

## Slide 8
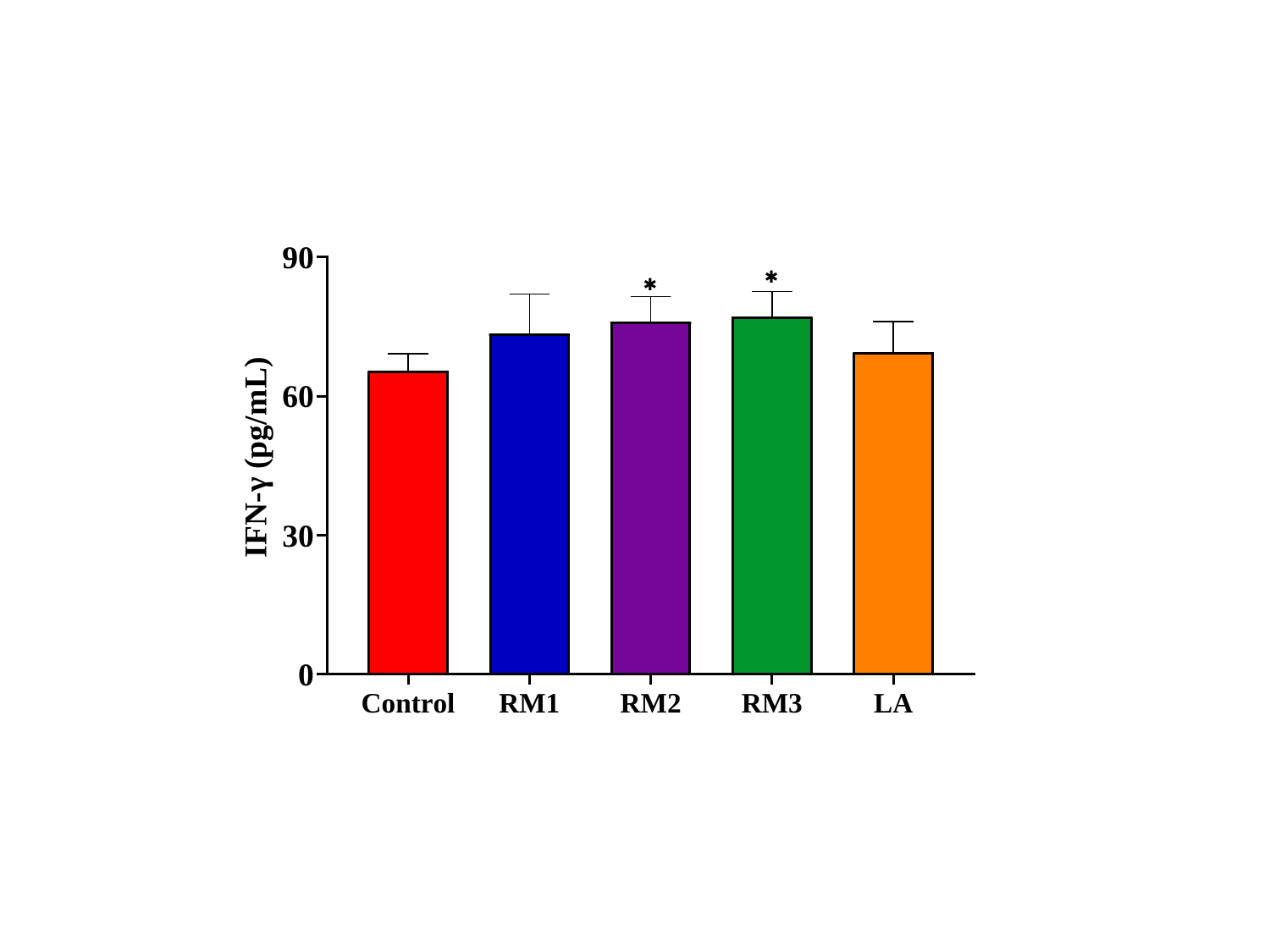

## Slide 9
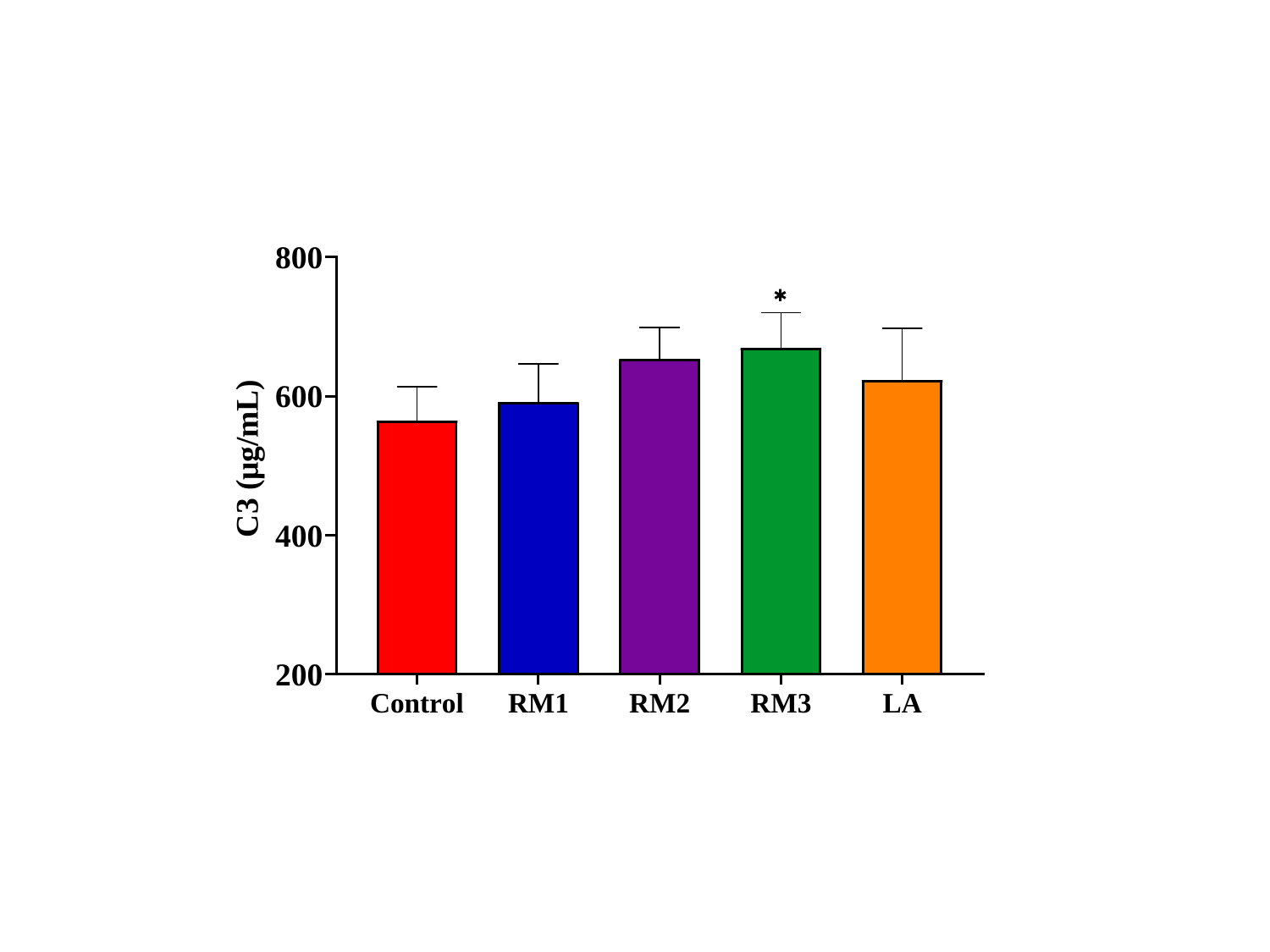

## Slide 10
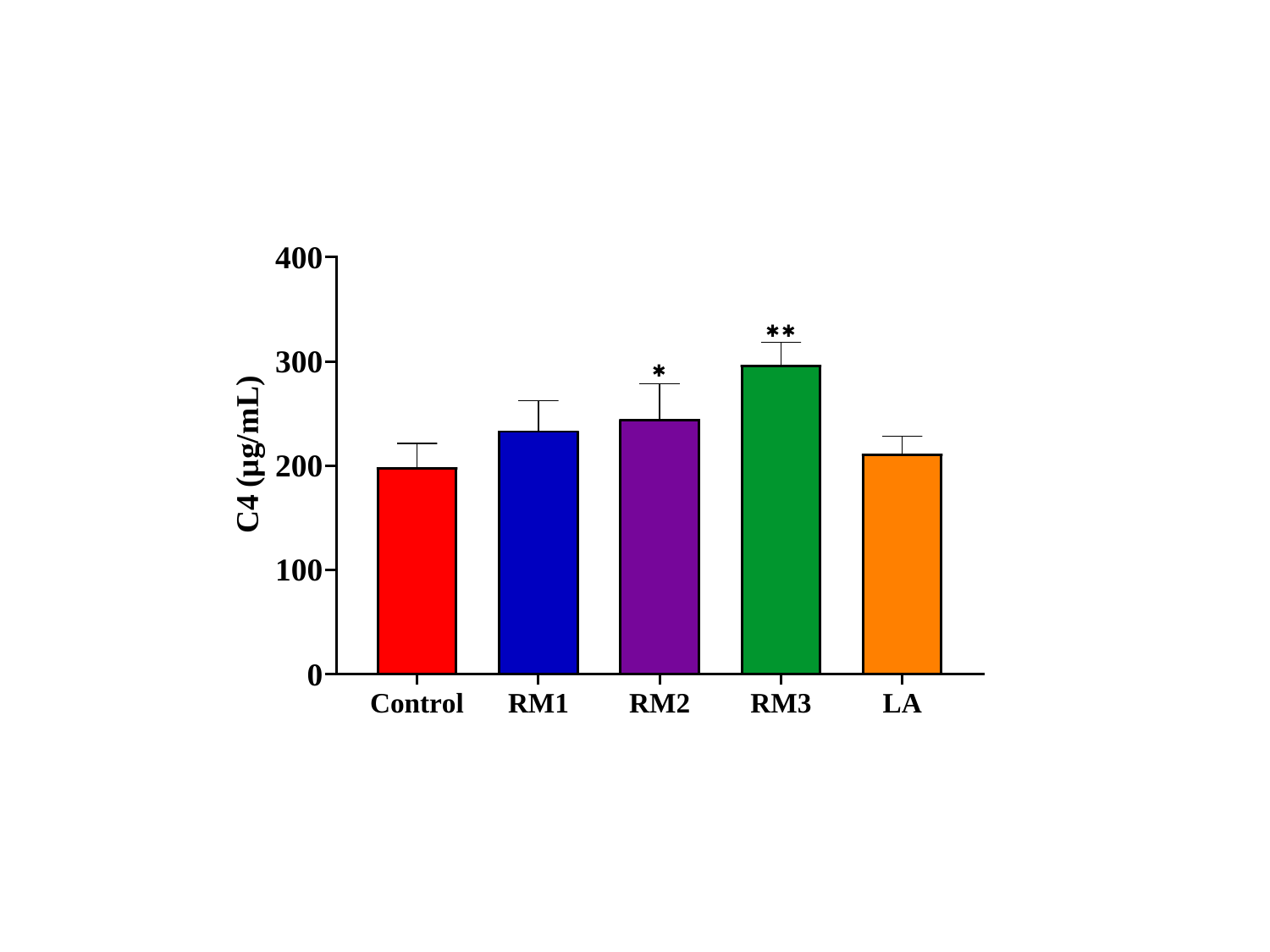

Supplement: Supplementary file 1 [file Data_Sheet_1.ZIP › the Article raw data/Serum cytokine, Complement and immunoglobulin by elisa/21d elisa试剂盒/21d免疫试剂盒(带数据源).pptx]

## Slide 1
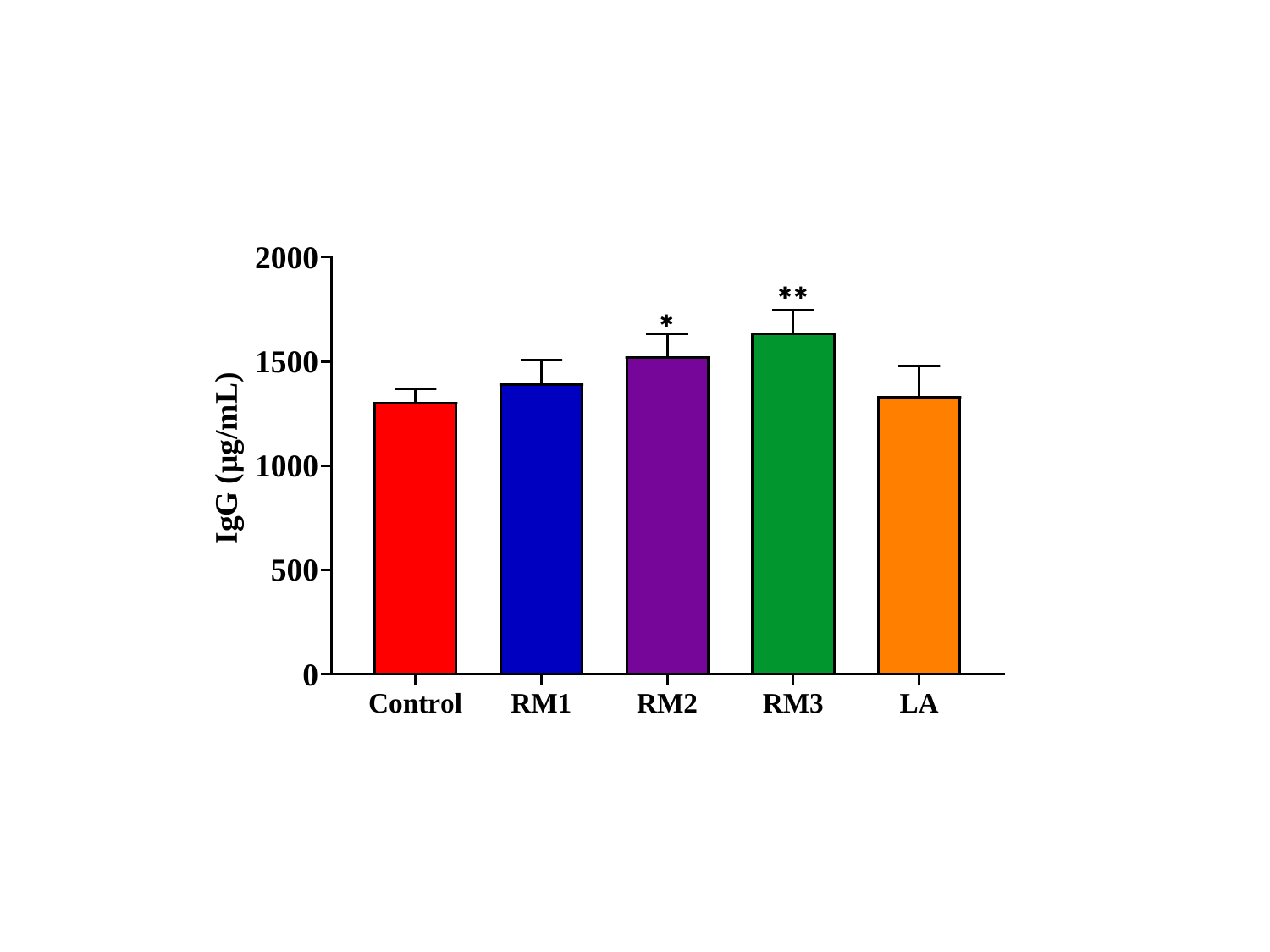

## Slide 2
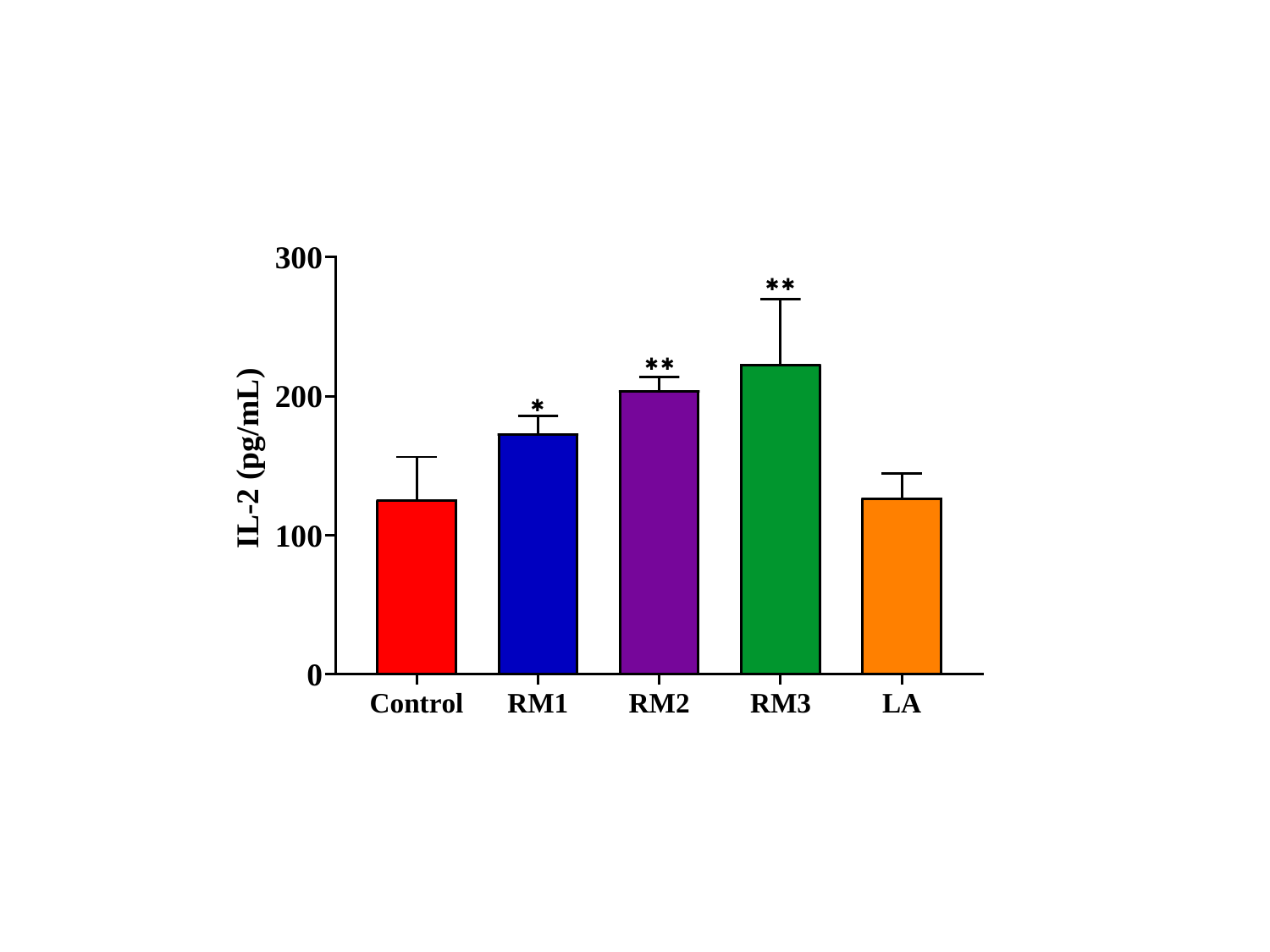

## Slide 3
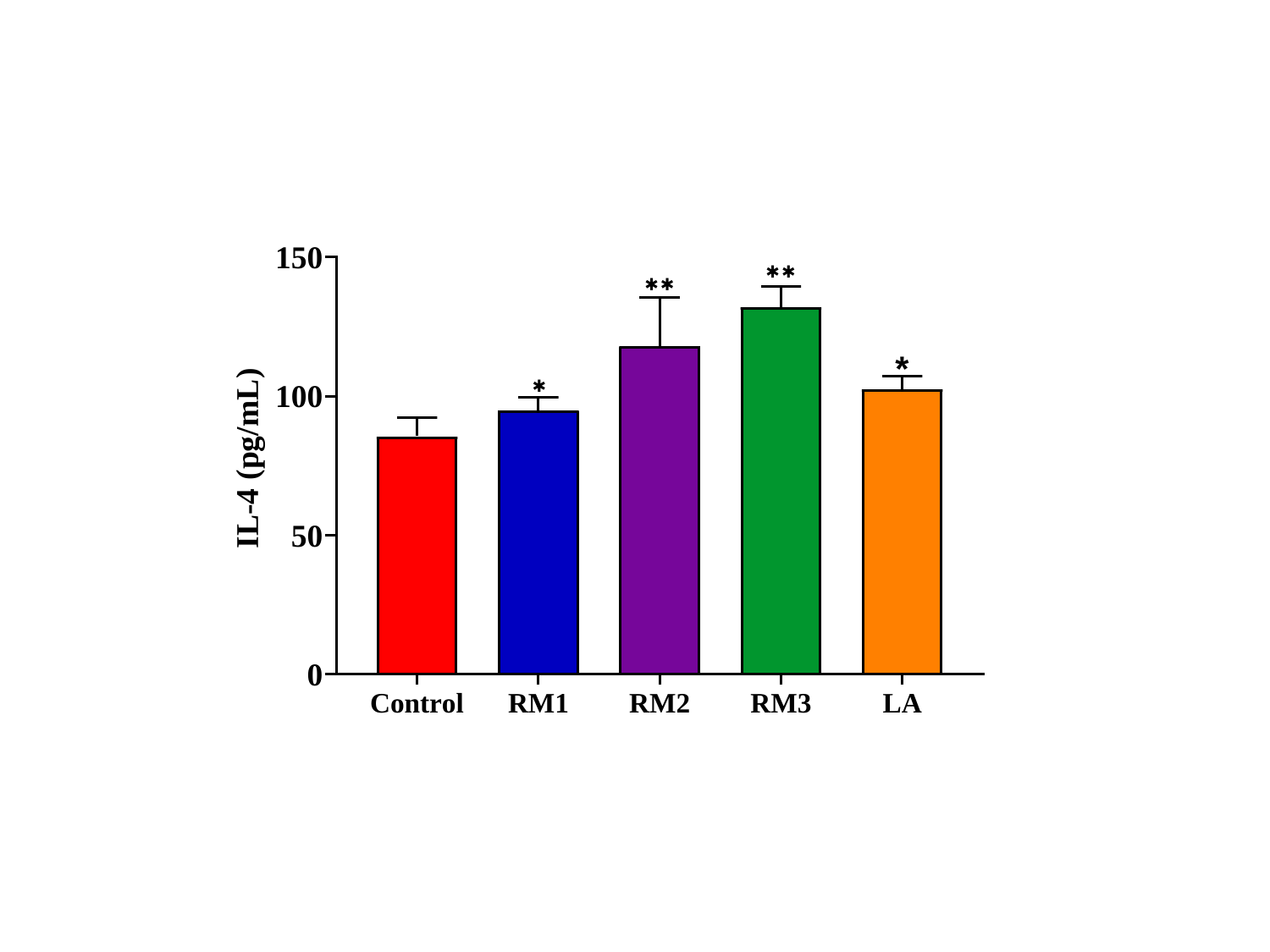

## Slide 4
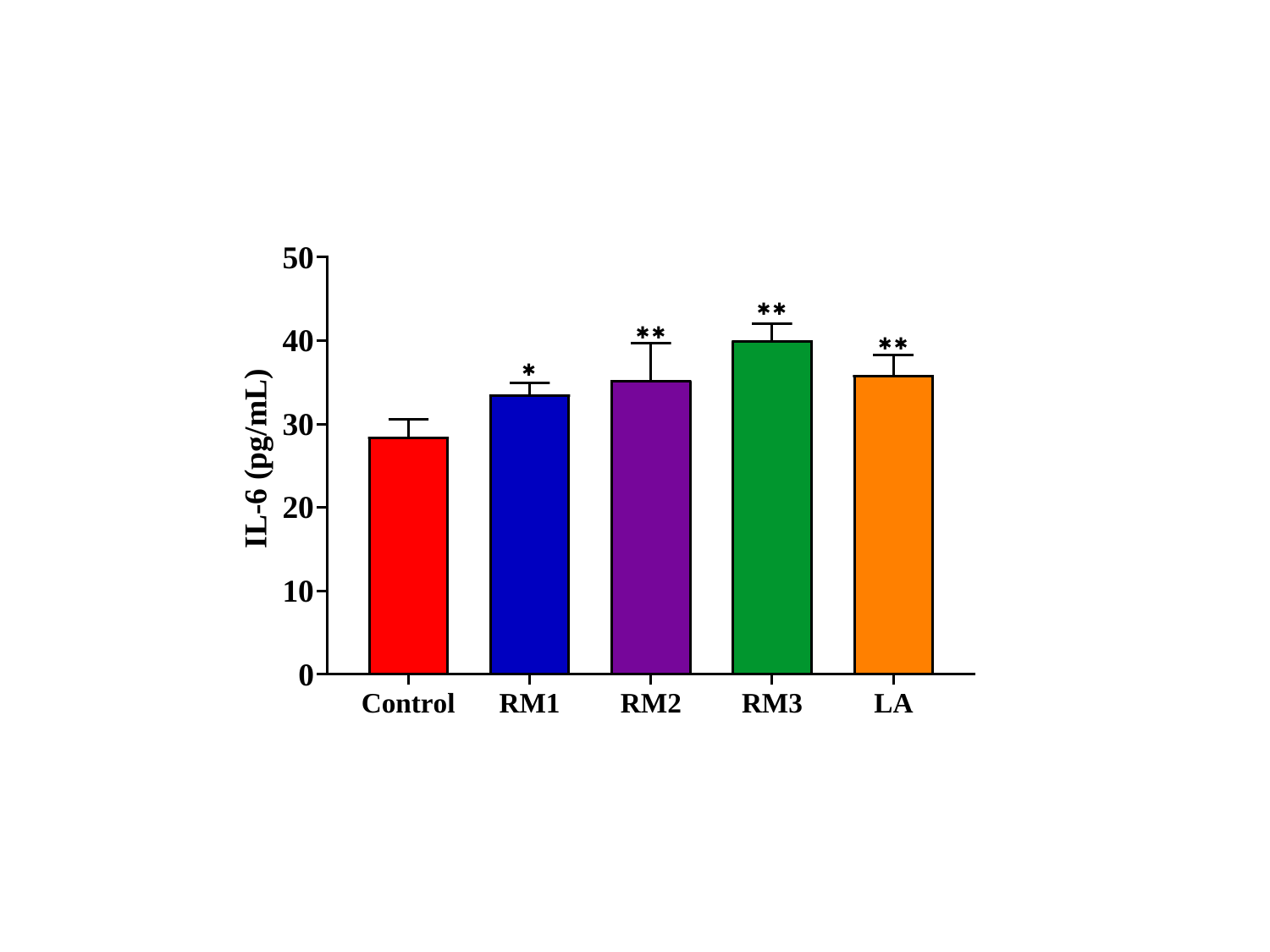

## Slide 5
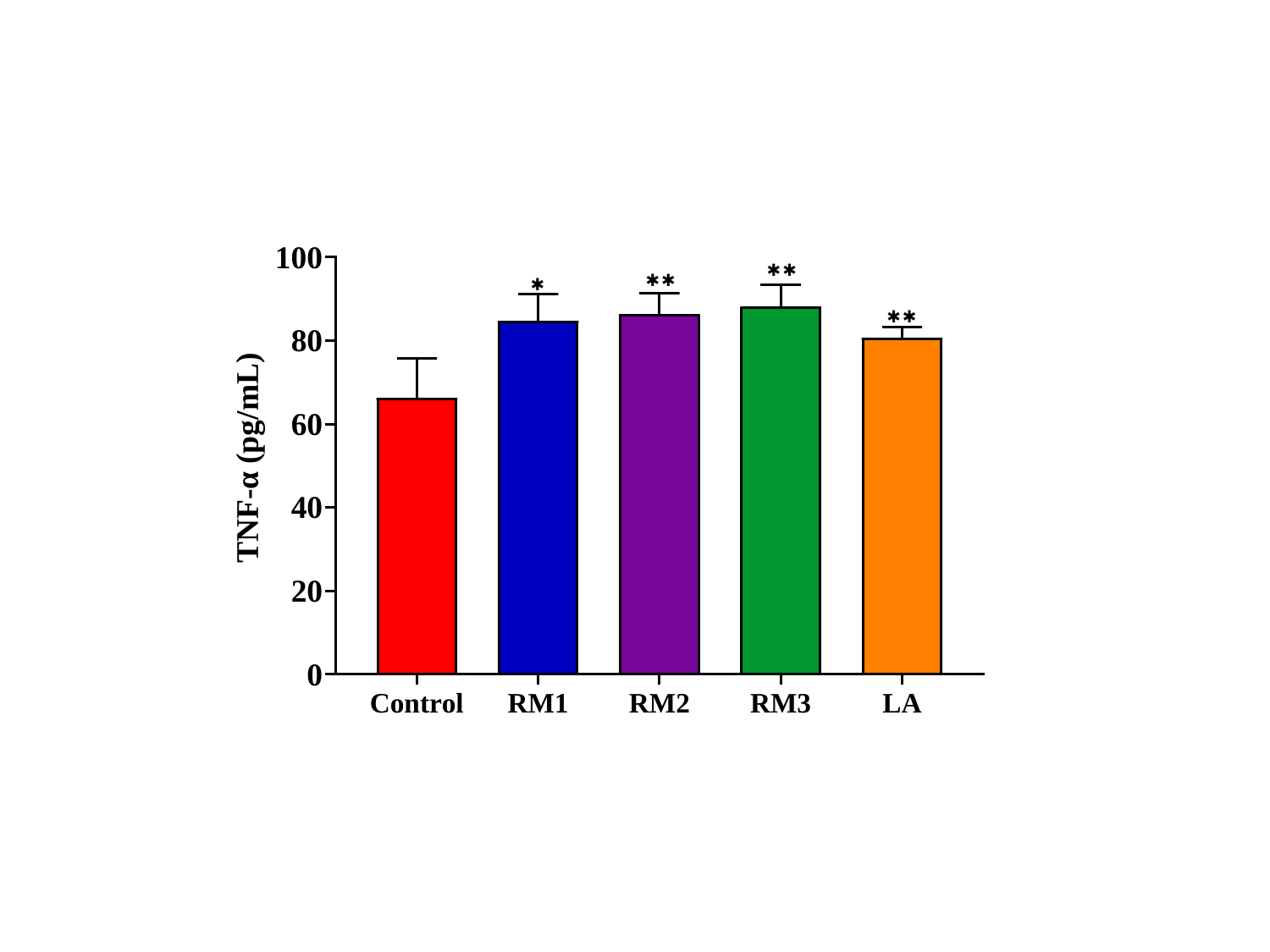

## Slide 6
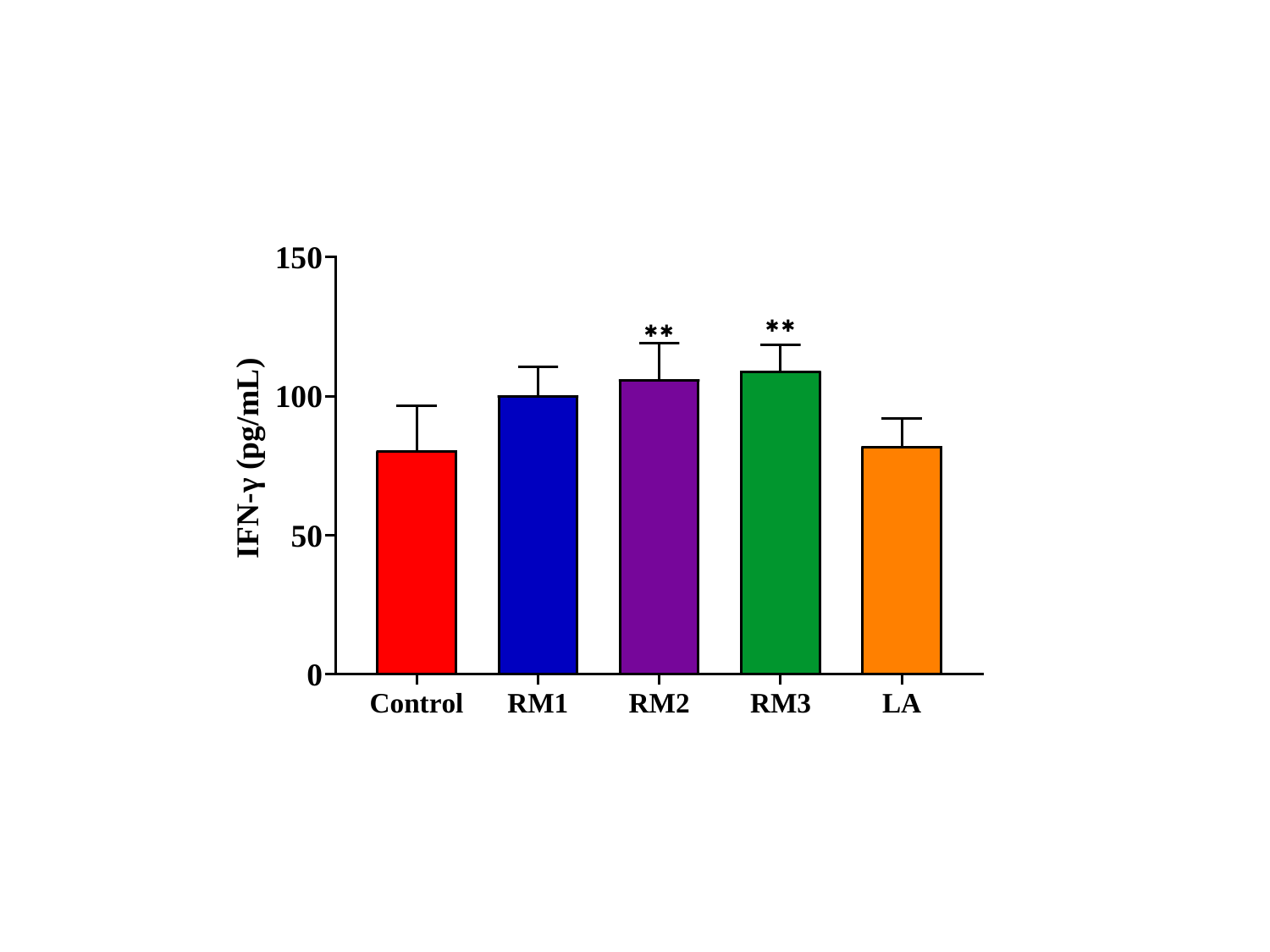

## Slide 7
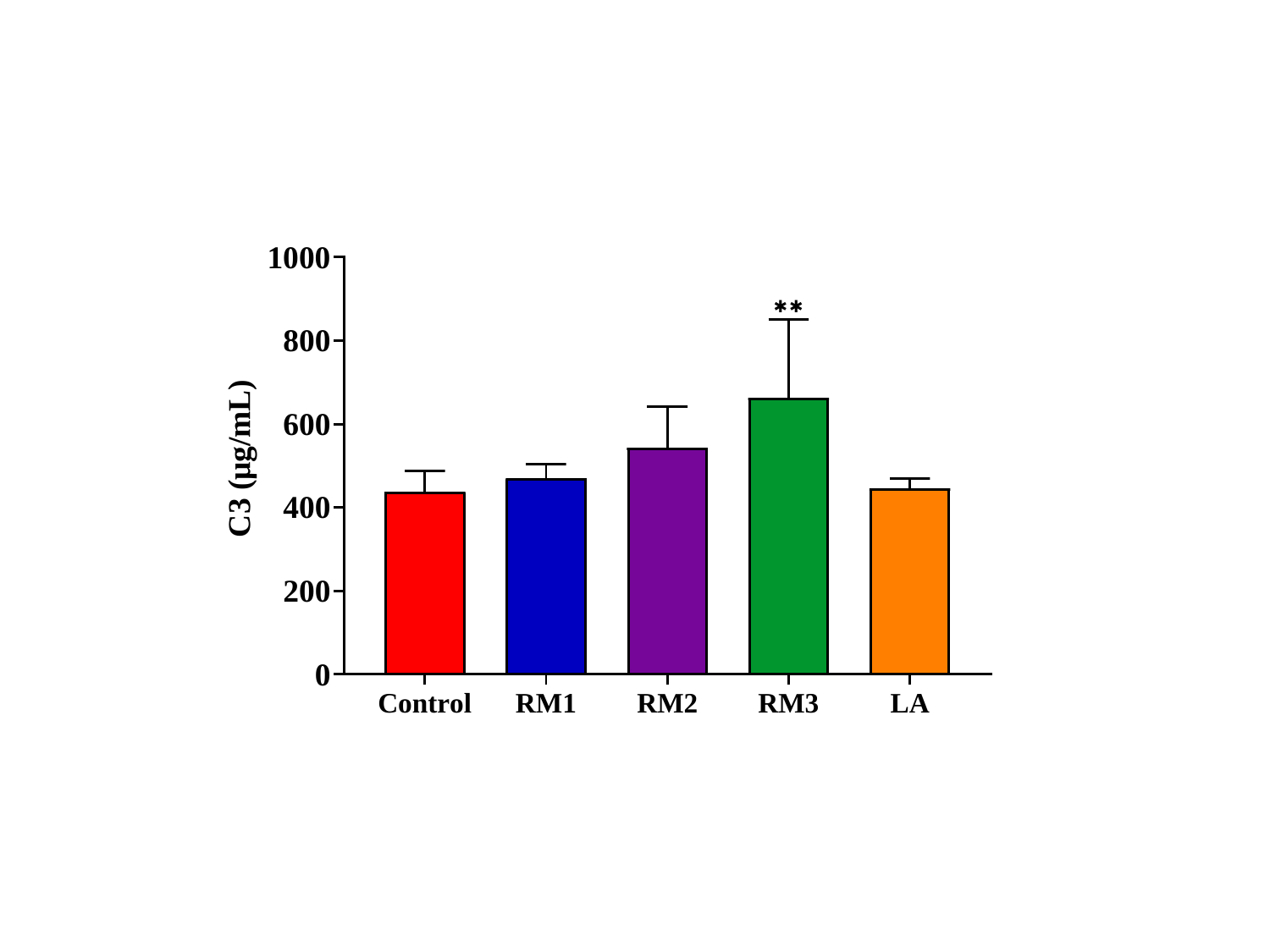

## Slide 8
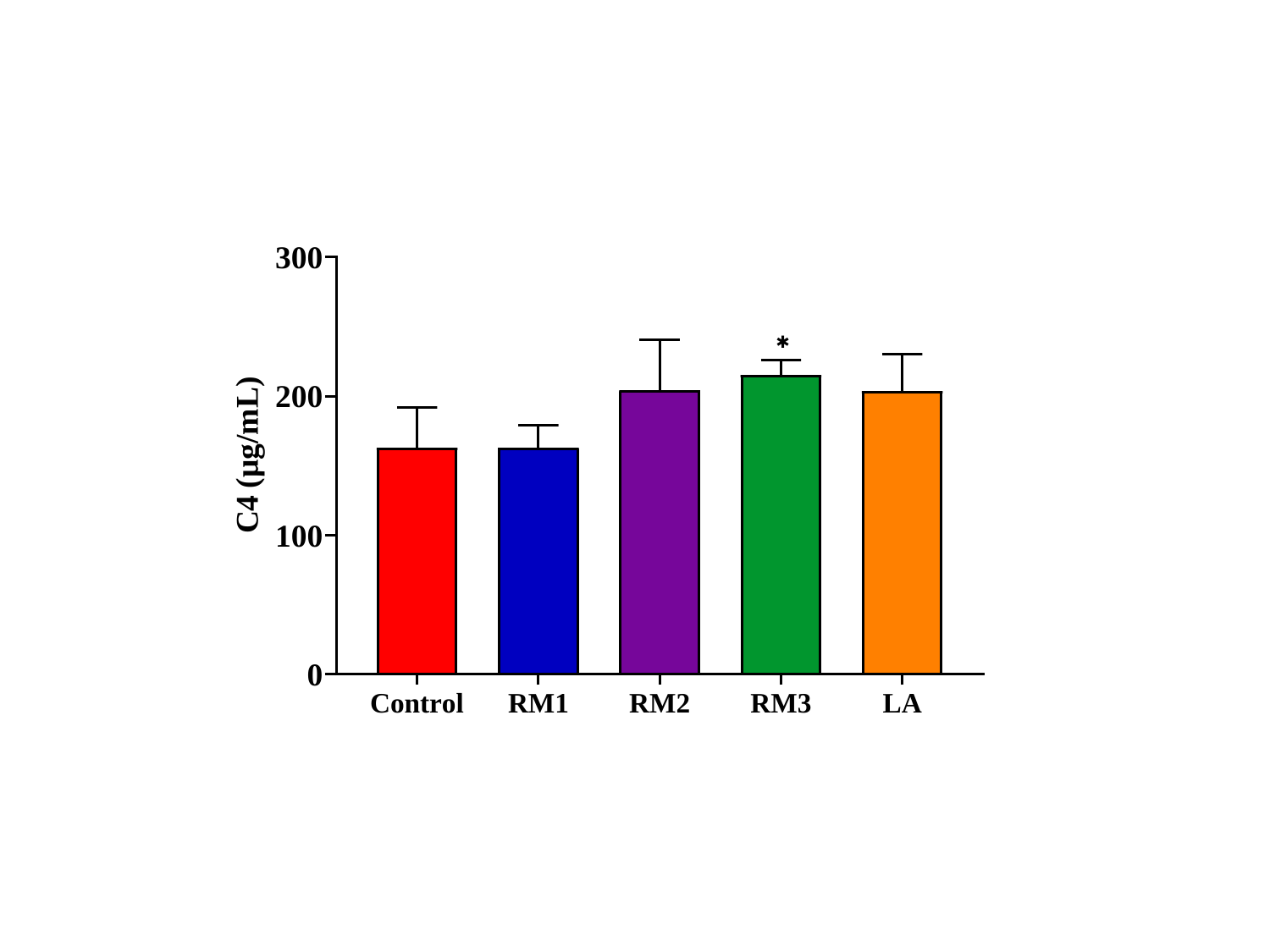

Supplement: Supplementary file 1 [file Data_Sheet_1.ZIP › the Article raw data/Serum cytokine, Complement and immunoglobulin by elisa/42d elisa试剂盒/42d免疫(带数据源).pptx]

## Slide 1
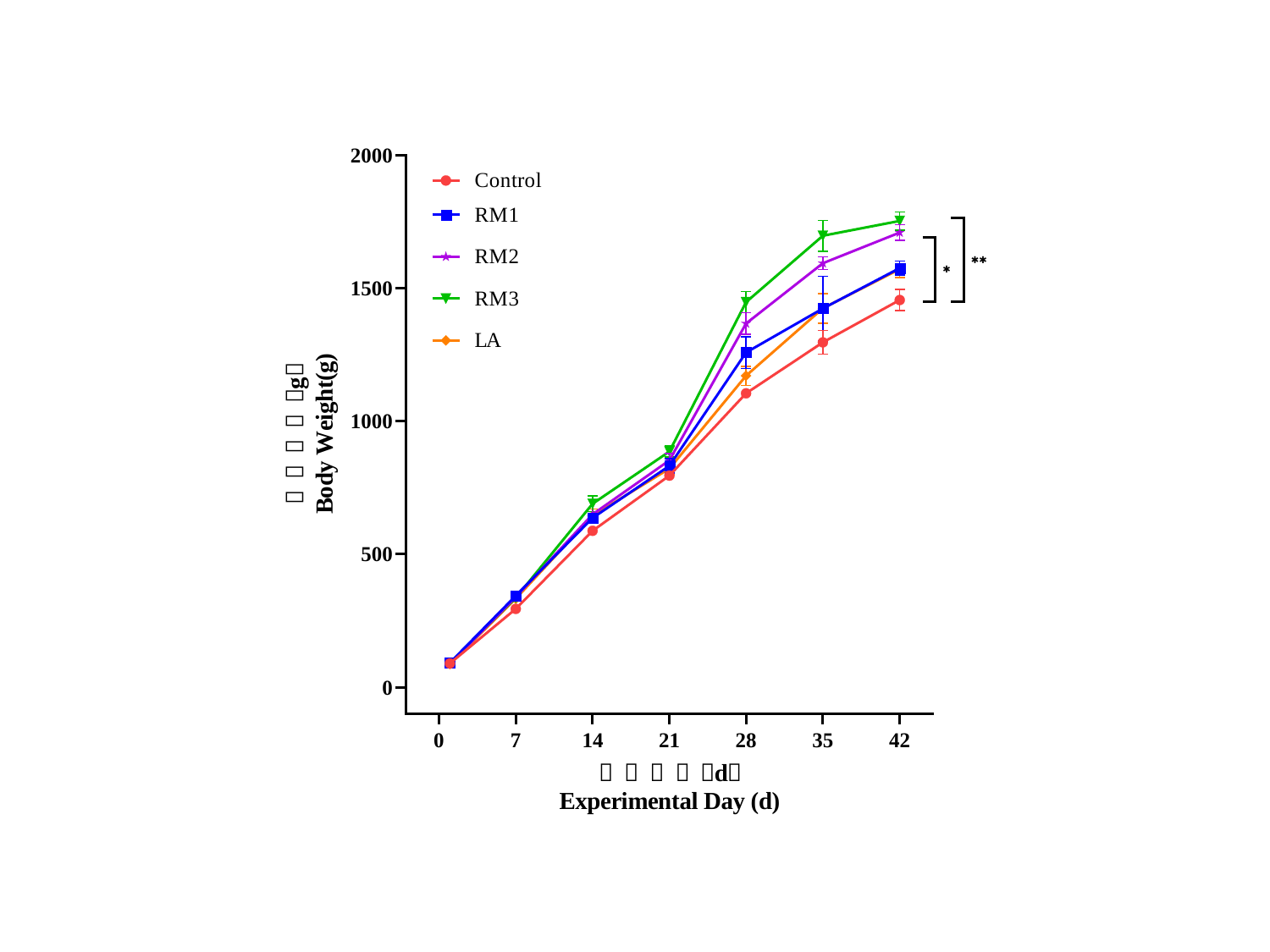

Supplement: Supplementary file 1 [file Data_Sheet_1.ZIP › the Article raw data/the growth performance of Leizhou black ducks(with raw data).pptx]
